# Supplementary material for: Inhibiting cholesterol synthesis halts rhabdomyosarcoma growth via ER stress and cell cycle arrest
Source: EMBO Mol Med. 2025 Nov 17;17(12):3586–606. doi: 10.1038/s44321-025-00336-x (PMC12686467; doi:10.1038/s44321-025-00336-x)
Supplement: Supplementary file 1 — Table EV1 [file 44321_2025_336_MOESM1_ESM.pdf]

Table EV1. Human primers used for RT-qPCR analysis.

	PRIMER (5'-3')
DHCR7 forward	TCCACAGCCATGTGACCAATGC
DHCR7 reverse	CGAAGTGGTCATGGCAGATGTC
HMGCR forward	ACTTCGTGTTCATGACTTTC
HMGCR reverse	GACATAATCATCTTGACCCTC
MVK forward	GGAAAGTGGACCTCAGCTTACC
MVK reverse	GCTTCTCCACTTGCTCTGAGGT
LSS forward	GACGACCGATTCACCAAGAGCA
LSS reverse	AGACATGCTCCTGGAAGGCAGT
MSMO1 forward	GCTGCCTTTGATTTGTGGAACCT
MSMO1 reverse	CTGCACAACCAAAGCATCTTGCC
hSQLE forward	CTCCAAGTTCAGGAAAAGCCTGG
hSQLE reverse	GAGAACTGGACTCGGGTTAGCT
hACAT2 forward	TGGTGCCTTAGCTGCTGTTCCT
hACAT2 reverse	GGCTTGTCTAACAGGATTCTGCC
IDI forward	GCCGCAGACTGTGCTCAAAGC
IDI reverse	CCTGTTGCTTGTCGAGGTGGTT
FDFT1 forward	TGTGACCTCTGAACAGGAGTGG
FDFT1 reverse	GCCCATAGAGTTGGCACGTTCT
MVD forward	AAGCGCGATGAAGAGCTGGTTC
MVD reverse	TCCTCGGTGAAGTCCTTGCTGA
PERK forward	GTCCCAAGGCTTTGGAATCTGTC
PERK reverse	CCTACCAAGACAGGAGTTCTGG
ATF4 forward	TTCTCCAGCGACAAGGCTAAGG
ATF4 reverse	CTCCAACATCCAATCTGTCCCG
CHOP forward	GGTATGAGGACCTGCAAGAGGT
CHOP reverse	CTTGTGACCTCTGCTGGTTCTG

GADD34 forward	TCCGACTGCAAAGGCGGCTCA
GADD34 reverse	CAGCCAGGAAATGGACAGTGAC
CCNE1 FWD	TGTGTCCTGGATGTTGACTGCC
CCNE1 REV	CTCTATGTCGCACCACTGATACC
CDC6 FWD	GGAGATGTTCGCAAAGCACTGG
CDC6 REV	GGAATCAGAGGCTCAGAAGGTG
PROX1 forward	CTGAAGACCTACTTCTCCGACG
PROX1 reverse	GATGGCTTGACGTGCGTACTTC
HPRT forward	CTCATGGACTGATTATGGACAGGAC
HPRT reverse	GCAGGTCAGCAAAGAACTTATAGCC
