# Supplementary material for: Inhibiting cholesterol synthesis halts rhabdomyosarcoma growth via ER stress and cell cycle arrest
Source: EMBO Mol Med. 2025 Nov 17;17(12):3586–606. doi: 10.1038/s44321-025-00336-x (PMC12686467; doi:10.1038/s44321-025-00336-x)
Supplement: Supplementary file 2 — Table EV2 [file 44321_2025_336_MOESM2_ESM.pdf]

Table EV2. Summary of statistical tests and P-values

Figure #	Statistical	Multiple comparison	Groups	P value	Summary
**Figure 1**					
Figure 1D	unpaired t-test	N/A	RD shSCR Vs shPROX1	0.034874	*
			KLHEL1 shSCR vs KLHEL1	0.040899	*
Figure 1E	unpaired t-test	N/A	ACAT2 Muscle VS FN-RMS	0.001705	**
			ACAT2 Muscle VS FP-RMS	0.000946	***
			HMGCS1 Muscle VS FN-RMS	0.001456	**
			HMGCS1 Muscle VS FP-RMS	0.015451	*
			HMGCR Muscle VS FN-RMS	5.18E-05	****
			HMGCR Muscle VS FP-RMS	0.00099	***
			MVK Muscle VS FN-RMS	0.006504	**
			MVK Muscle VS FP-RMS	0.002753	**
			MVD Muscle VS FN-RMS	0.051106	ns
			MVD Muscle VS FP-RMS	0.016414	*
			IDI1 Muscle VS FN-RMS	2.88E-07	****
			IDI1 Muscle VS FP-RMS	0.001796	**
			GGPS1	0.002456	**

			Muscle VS FN-RMS		
			GGPS1 Muscle VS FP-RMS	0.004343	**
			FDPS Muscle VS FN-RMS	0.002494	**
			FDPS Muscle VS FP-RMS	0.00062	***
			SQLE Muscle VS FN-RMS	0.000881	***
			SQLE Muscle VS FP-RMS	9.53E-05	****
			LSS Muscle VS FN-RMS	0.030908	*
			LSS Muscle VS FP-RMS	0.021138	*
			MSMO1 Muscle VS FN-RMS	0.014898	*
			MSMO1 Muscle VS FN-RMS	0.007186	**
			DHCR24 Muscle VS FN-RMS	0.546352	ns
			DHCR24 Muscle VS FP-RMS	0.161393	ns
			DHCR7 Muscle VS FN-RMS	0.016406	*
			DHCR7 Muscle VS FN-RMS	0.01288	*
**Figure 2**					
2B	One-way ANOVA	Dunnett's multiple comparison test	RD shSCR vs shDHCR7-1	7.92E-08	****

			RD shSCR vs shDHCR7-2	7.65E-08	****
			RD shSCR vs shDHCR7-3	9.95E-08	****
2C	One-way ANOVA	Dunnett's multiple comparison test	RD shSCR vs shDHCR7-1	3.1E-14	****
			RD shSCR vs shDHCR7-2	3.8E-14	****
			RD shSCR vs shDHCR7-3	5.62E-13	****
2E	One-way ANOVA	Dunnett's multiple comparison test	RD shSCR vs shDHCR7-1	6.67E-09	****
			RD shSCR vs shDHCR7-2	8.15E-09	****
			RD shSCR vs shDHCR7-3	3.77E09	****
2F	unpaired t-test	N/A	RD shSCR vs shDHCR7-2	6,27E-09	****
2G	One-way ANOVA	Dunnett's multiple comparison test	KLHEL1 shSCR vs shDHCR7-1	1.87E-06	****
			KLHEL1 shSCR vs shDHCR7-2	3.35E-06	****
			KLHEL1 shSCR vs shDHCR7-3	6.47E-06	****
2H	One-way ANOVA	Dunnett's multiple comparison test	KLHEL1 shSCR vs shDHCR7-1	4E-15	****
			KLHEL1 shSCR vs shDHCR7-2	4E-15	****
			KLHEL1 shSCR vs shDHCR7-3	4E-15	****

2J	One-way ANOVA	Dunnett's multiple comparison test	KLHEL1 shSCR vs shDHCR7-1	1.11E-08	****
			KLHEL1 shSCR vs shDHCR7-2	6.22E-08	****
			KLHEL1 shSCR vs shDHCR7-2	1.0E08	****
2K	unpaired t-test	N/A	KLHEL1 shSCR vs shDHCR7-2	2.13E-12	****
2L	One-way ANOVA	Dunnett's multiple comparison test	RD DMSO vs AY9944 2uM	0.09	ns
			RD DMSO vs AY9944 5uM	2.55E-08	****
			RD DMSO vs AY9944 10uM	<1E-15	****
			RD DMSO vs AY9944 20uM	<1E-15	****
2M	unpaired t-test	N/A	RD DMSO vs AY9944 10uM	3,97E-07	****
2N	One-way ANOVA	Dunnett's multiple comparison test	KLHEL1 DMSO vs AY9944 2uM	0,018498453	*
			KLHEL1 DMSO vs AY9944 5uM	2,01E-13	****
			KLHEL1 DMSO vs AY9944 10uM	<1E-15	****
			KLHEL1 DMSO vs AY9944 20uM	<1E-15	****
2O	unpaired t-test	N/A	KLHEL1	7.16E-08	****

			DMSO vs AY9944 10uM		
2P	unpaired t-test	N/A	Myoblast DMSO vs AY9944 10uM	0.8624	ns
2Q	unpaired t-test	N/A	Myoblast DMSO vs AY9944 10uM	0.162	ns
2R	unpaired t-test	N/A	Astrocyte DMSO vs AY9944 10uM	0.0981	ns
2S	unpaired t-test	N/A	Astrocyte DMSO vs AY9944 10uM	0.082	ns
**Figure 3**					
3A			RD		
	unpaired t-test	N/A	Day 7	0.0026	**
			Day 14	6.34E-05	****
			Day 21	5.43E-05	****
			Day 28	0.0002	****
			Day 35	6.96E-05	****
3A			KLHEL1		
	unpaired t-test	N/A	Day 7	0.0003	****
			Day 14	7.47E-05	****
			Day 21	6.94E-07	****
			Day 28	8.43E-06	****
3C	unpaired t-test	N/A	RD shSCR VS shDHCR7	7.63E-06	****
			KLHEL1 shSCR VS shDHCR7	9.39E-06	****
3L	unpaired t-test	N/A	RD shSCR VS shDHCR7	1.41E-07	****

3M	unpaired t-test	N/A	RD shSCR VS shDHCR7	1.94E-13	****
3N	unpaired t-test	N/A	RD shSCR VS shDHCR7	5.64E-05	****
3O	unpaired t-test	N/A	RD shSCR VS shDHCR7	0.0431	*
**Figure 4**					
4K	unpaired t-test		Cyclin E		
			RD	0.0022	**
			KLHEL1	0.0004	***
			RH30	0.0007	***
			CDC6		
			RD	0.0066	**
			KLHEL1	0.0036	**
			RH30	0.0027	**
4L	unpaired t-test	N/A	RD G1	0.467	ns
			RD S	0.057	ns
			G2	0.049	*
			RH30 G1	0.0012	**
			RH30 S	0.057	ns
			RH30 G2	0.008	**
**Figure 5**					
5F	unpaired t-test	N/A	PERK	0.0002	***
			ATF4	0.0002	***
			CHOP	1.78E-05	****
			GADD34	8.58E-05	****
5G	unpaired t-test		PERK	0.0002	***
			ATF4	0.0002	***
			CHOP	0.0007	***
			GADD34	3.26E-05	****
5H	unpaired t-test		RD		
			PERK	0.039898	*
			eIF2a	0.001476	**
			P-eIF2a	0.000144	***
			ATF4	1.64E-06	****
			CHOP	0.0002	***
			KLHEL1		
			PERK	0.0011	**

			eIF2a	0.0742	ns
			P-eIF2a	4.85E-05	****
			ATF4	0.0005	***
			CHOP	0.0281	*
**Extended Figures**					
**EV1**					
EV1A	One-way ANOVA	Dunnett's multiple comparison test	RD shSCR vs shHMGCR-1	0.0004	***
			RD shSCR vs shHMGCR-2	0.0036	**
EV1B	One-way ANOVA	Dunnett's multiple comparison test	RD shSCR vs shHMGCR-1	7.56E-05	****
			RD shSCR vs shHMGCR-2	O.0010	***
EV1D	One-way ANOVA	Dunnett's multiple comparison test	RD shSCR vs shHMGCR-1	3.65E-06	****
			RD shSCR vs shHMGCR-2	4.1E-06	****
EV1E	One-way ANOVA	Dunnett's multiple comparison test	KLHEL1 shSCR vs shHMGCR-1	3.24E-06	****
			KLHEL1 shSCR vs shHMGCR-2	0.0018	**
EV1F	One-way ANOVA	Dunnett's multiple comparison test	RD shSCR vs shHMGCR-1	4.45E-06	****
			RD shSCR vs shHMGCR-2	5.62E-05	****
EV1H	One-way ANOVA	Dunnett's multiple comparison test	RD shSCR vs shHMGCR-1	6.3E-06	****
			RD shSCR vs shHMGCR-2	1.69E-05	****

**EV3**					
EV3A	unpaired t-test	N/A	RD shSCR vs shDHCR7-2	7.44E-08	****
			KLHEL1 shSCR vs shDHCR7-2	3.35E-06	****
EV3K	unpaired t-test	N/A	RD shSCR vs shDHCR7-2	0.0011	**
EV3L	unpaired t-test	N/A	RD shSCR VS shDHCR7	3.78E-06	****
EV3M	unpaired t-test	N/A	RD shSCR VS shDHCR7	1.6E-06	****
EV3N	unpaired t-test	N/A	RD shSCR VS shDHCR7	0.005	**
