# Supplementary figures and images for: Inhibiting cholesterol synthesis halts rhabdomyosarcoma growth via ER stress and cell cycle arrest
Source: EMBO Mol Med. 2025 Nov 17;17(12):3586–606. doi: 10.1038/s44321-025-00336-x (PMC12686467; doi:10.1038/s44321-025-00336-x)

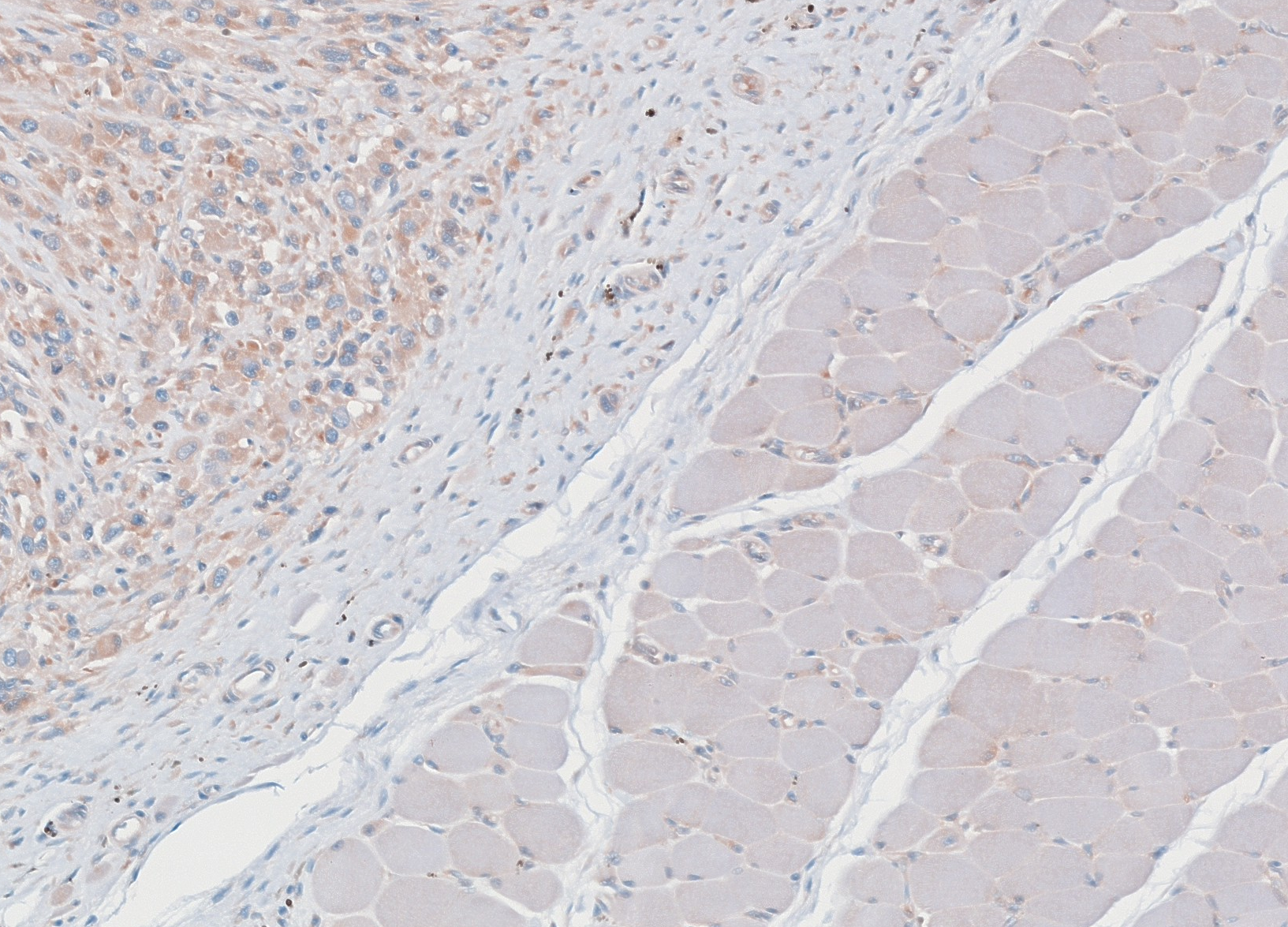

Supplement: Supplementary file 6 — Source data Fig. 1 [file 44321_2025_336_MOESM6_ESM.zip › Figure 1/Figure 1G/YP13-53991 cancer and health muscle DHCR7 .tif]

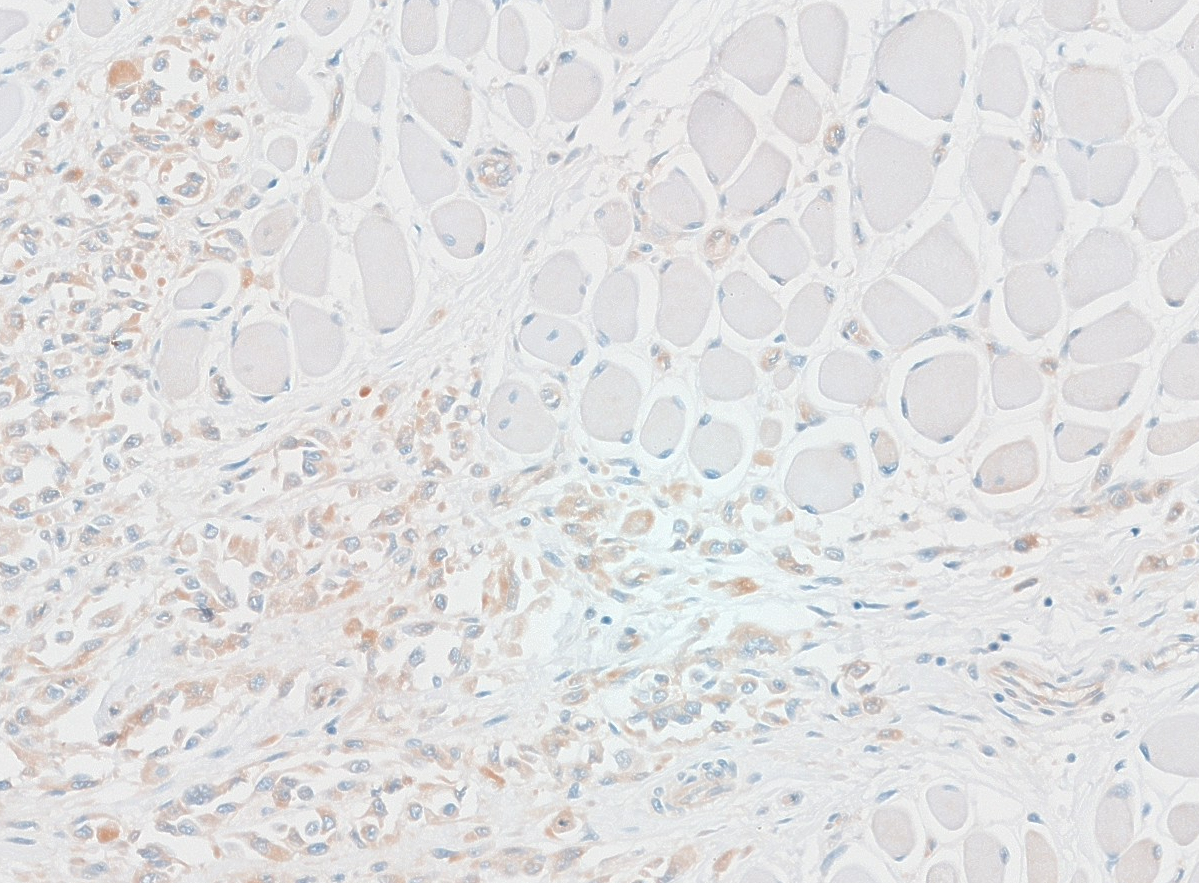

Supplement: Supplementary file 6 — Source data Fig. 1 [file 44321_2025_336_MOESM6_ESM.zip › Figure 1/Figure 1G/LP950609.tif cancer and healthy muscle DHCR7.tif]

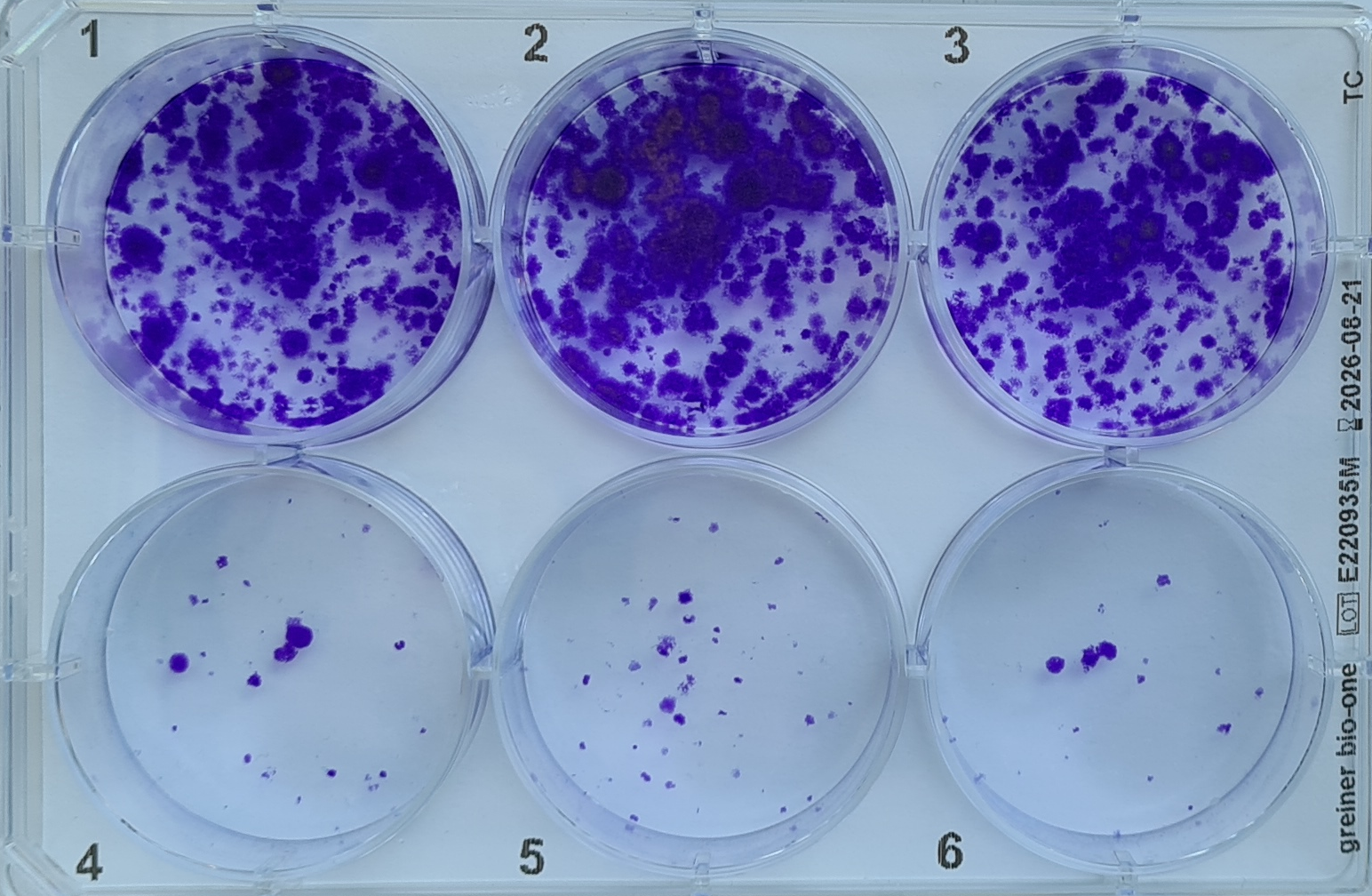

Supplement: Supplementary file 7 — Source data Fig. 2 [file 44321_2025_336_MOESM7_ESM.zip › Figure 2/Fig. 2D RD shSCR shDHCR 00 colony.tif]

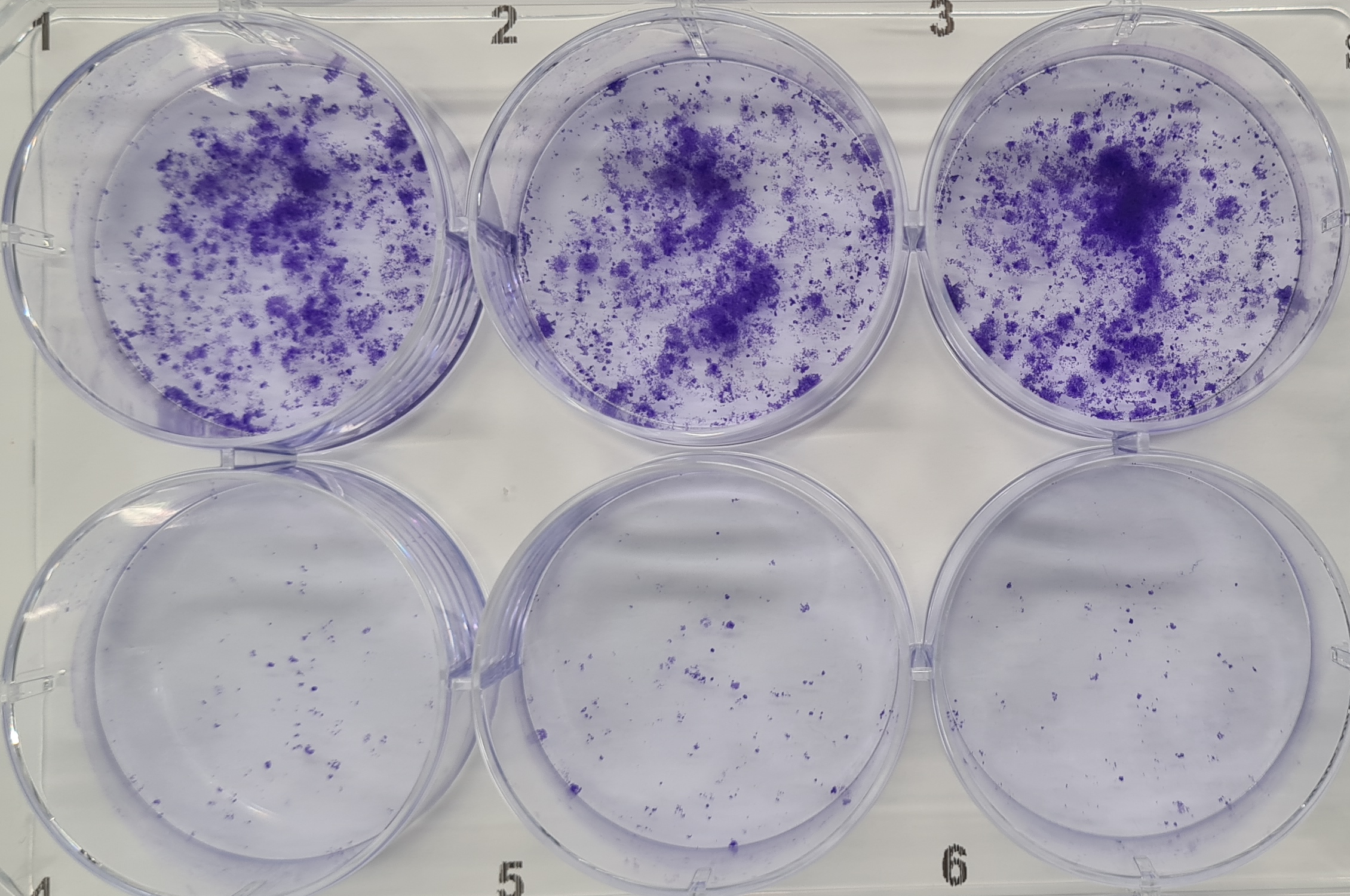

Supplement: Supplementary file 7 — Source data Fig. 2 [file 44321_2025_336_MOESM7_ESM.zip › Figure 2/Fig. 2I KL shSCR shDHCR7 00 colony.tif]

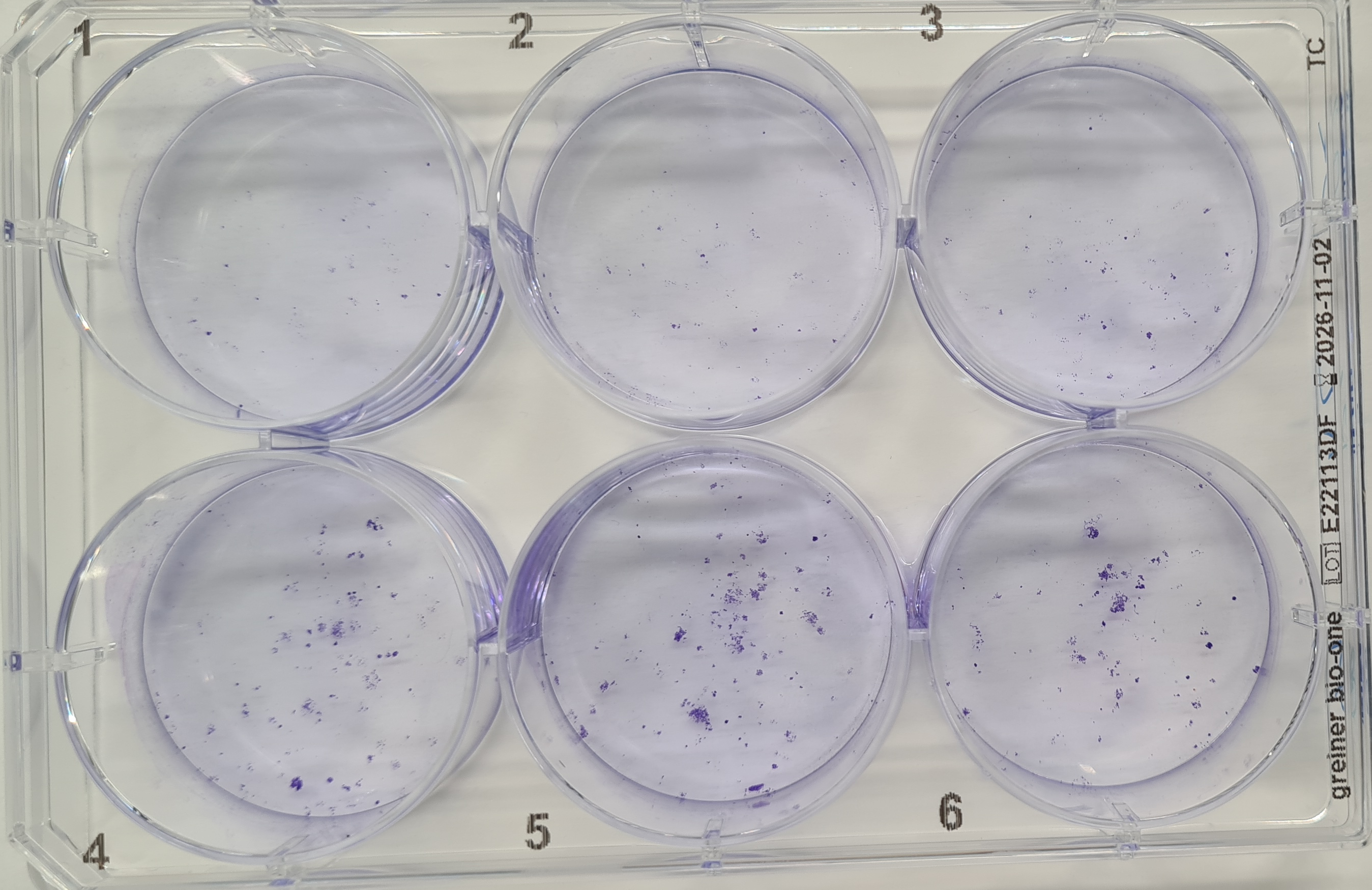

Supplement: Supplementary file 7 — Source data Fig. 2 [file 44321_2025_336_MOESM7_ESM.zip › Figure 2/Fig. 2I KLHEL1 shDHCR7 02 98 colony.tif]

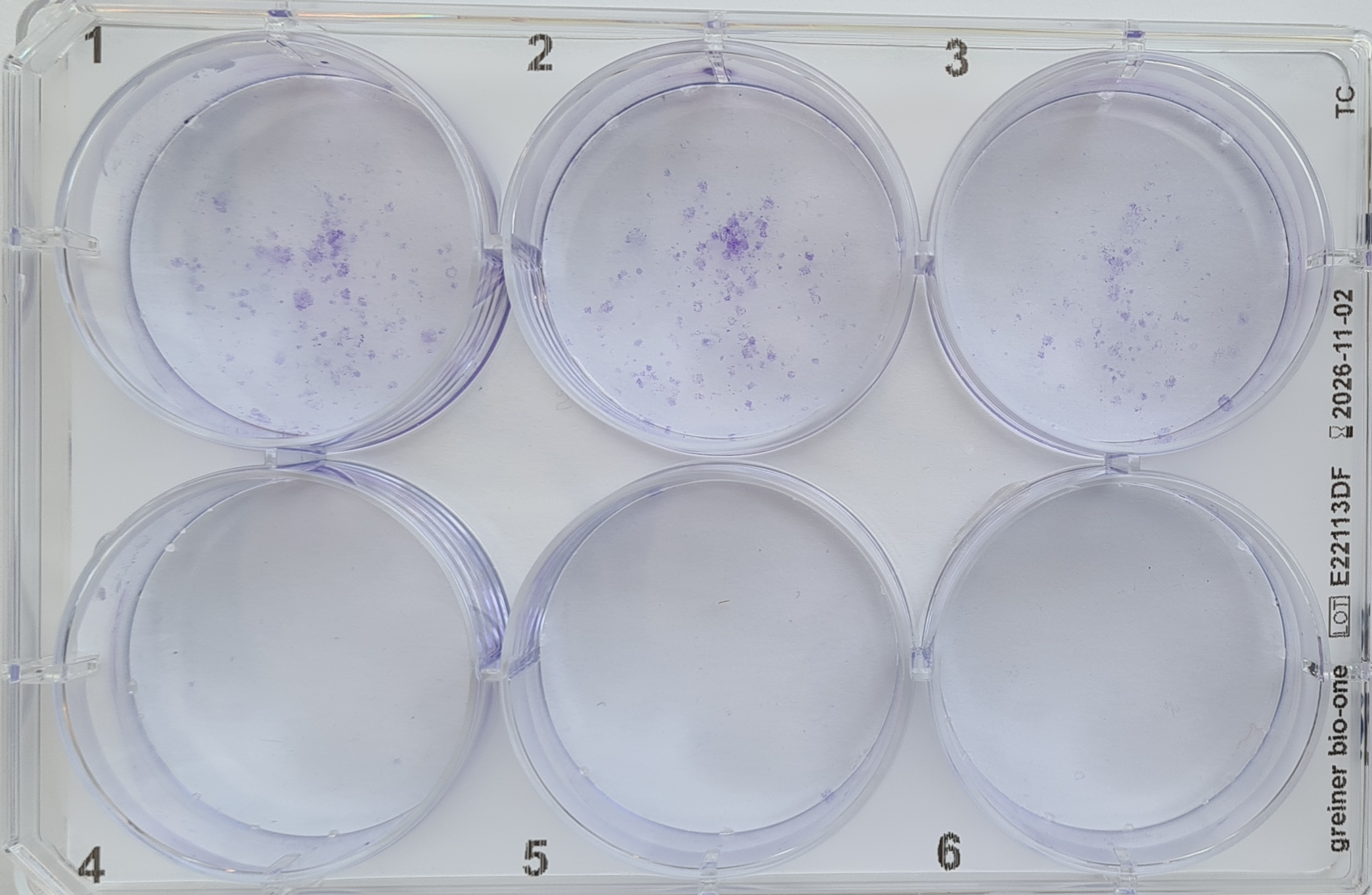

Supplement: Supplementary file 7 — Source data Fig. 2 [file 44321_2025_336_MOESM7_ESM.zip › Figure 2/Fig. 2D RD shDHCR 02 98 colony.tif]

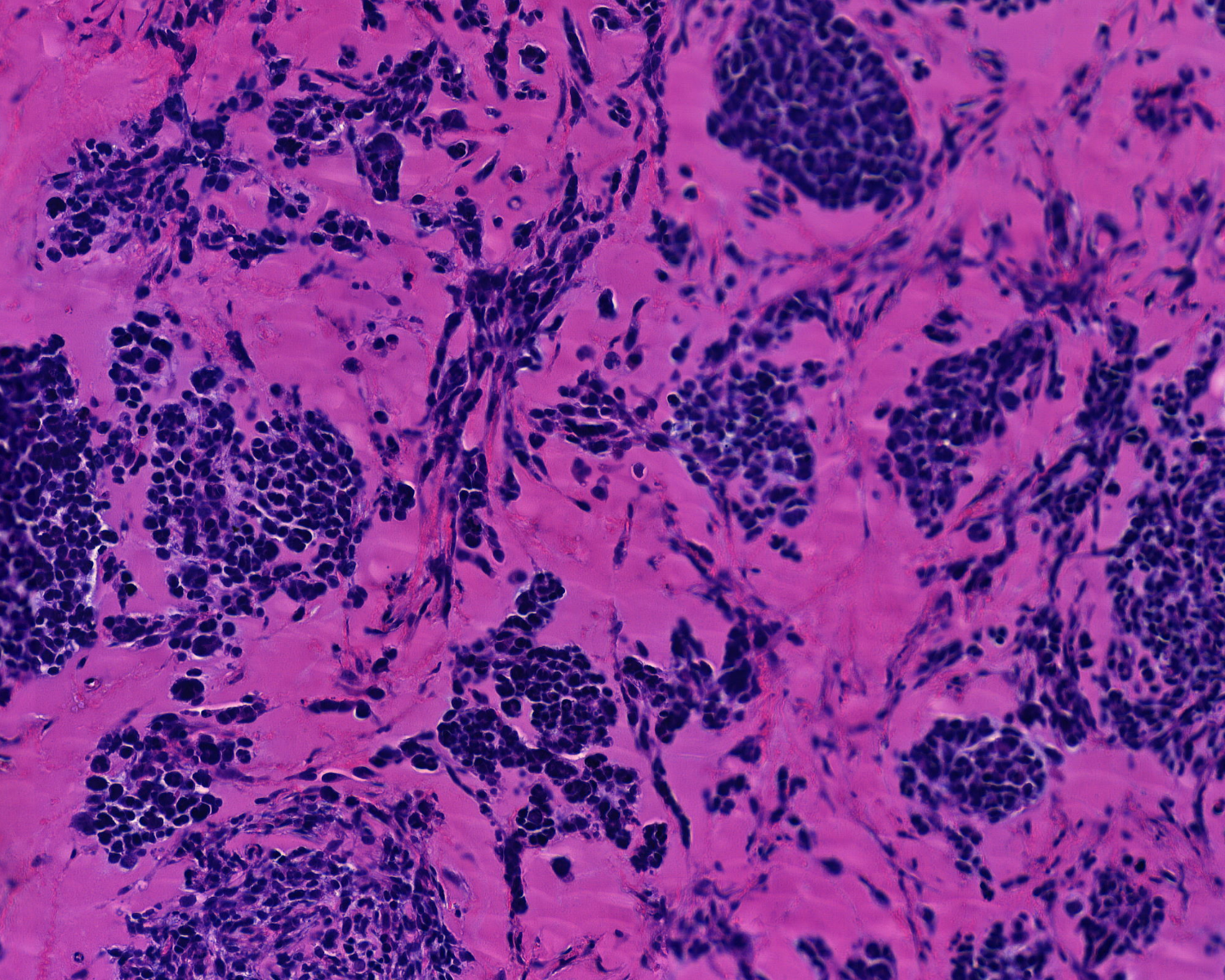

Supplement: Supplementary file 8 — Source data Fig. 3 [file 44321_2025_336_MOESM8_ESM.zip › Figure 3/Fig. 3D, H RD tumor H and E/2352 shDHCR.tif]

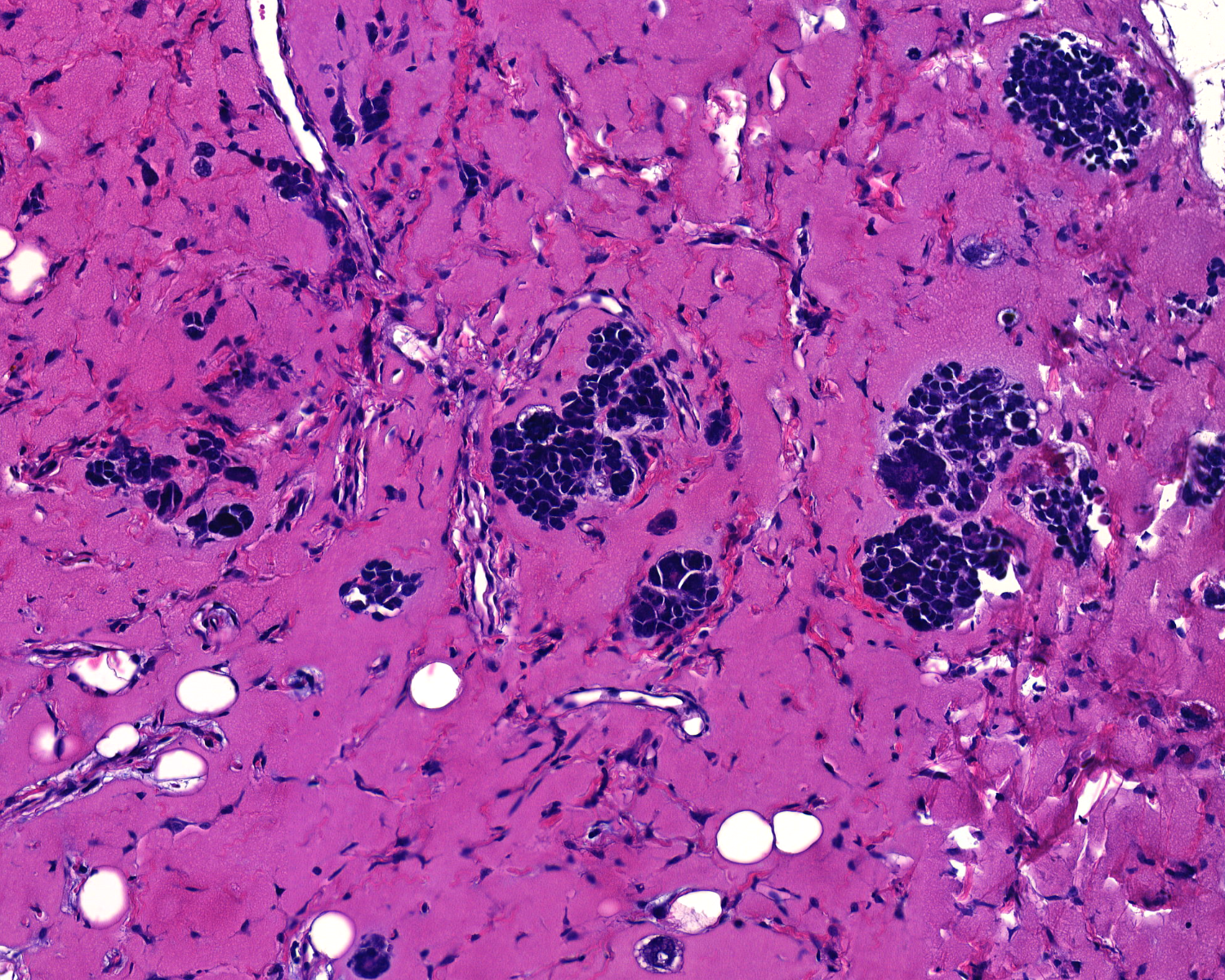

Supplement: Supplementary file 8 — Source data Fig. 3 [file 44321_2025_336_MOESM8_ESM.zip › Figure 3/Fig. 3D, H RD tumor H and E/2353 shDHCR7.tif]

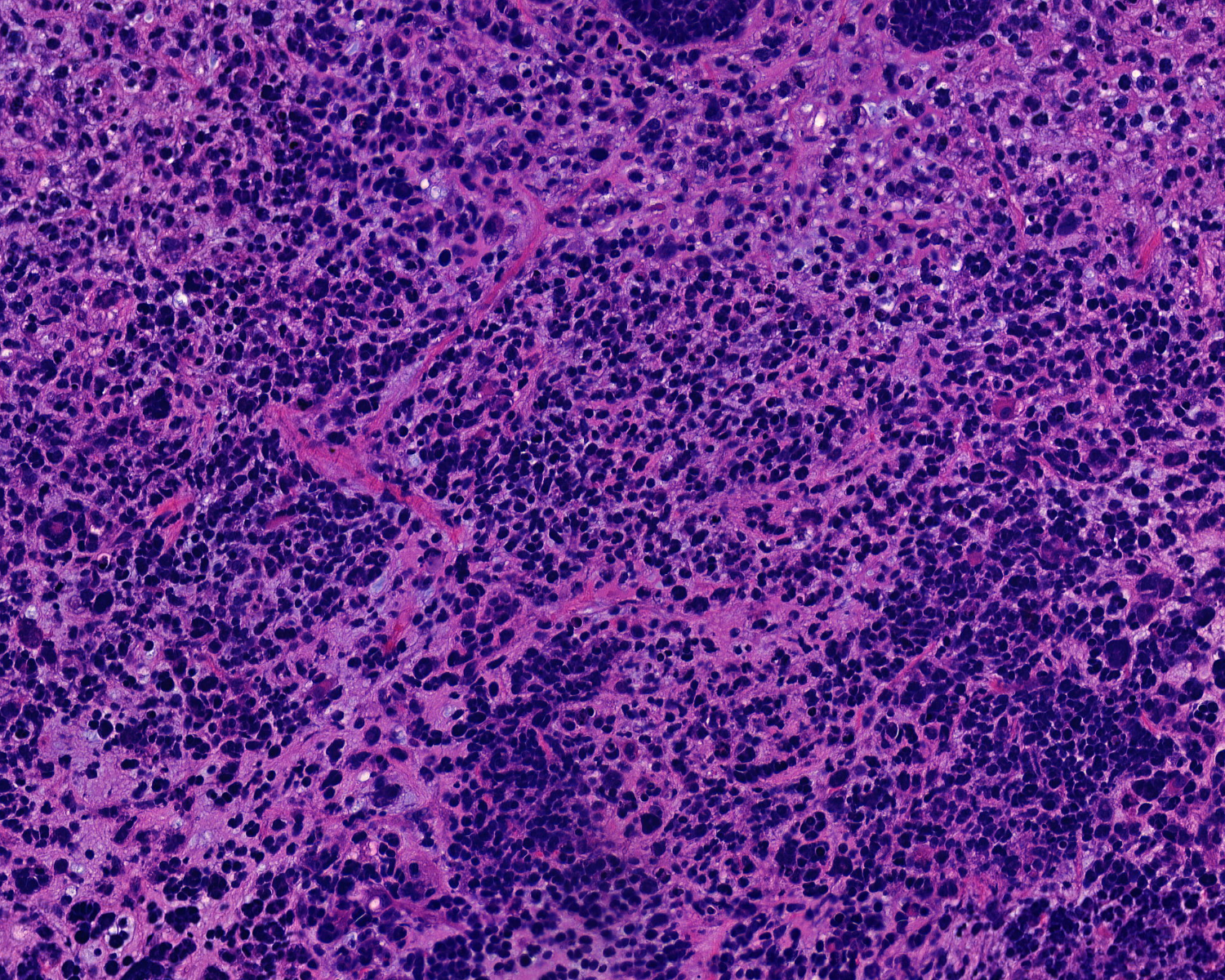

Supplement: Supplementary file 8 — Source data Fig. 3 [file 44321_2025_336_MOESM8_ESM.zip › Figure 3/Fig. 3D, H RD tumor H and E/2356 CTRL.tif]

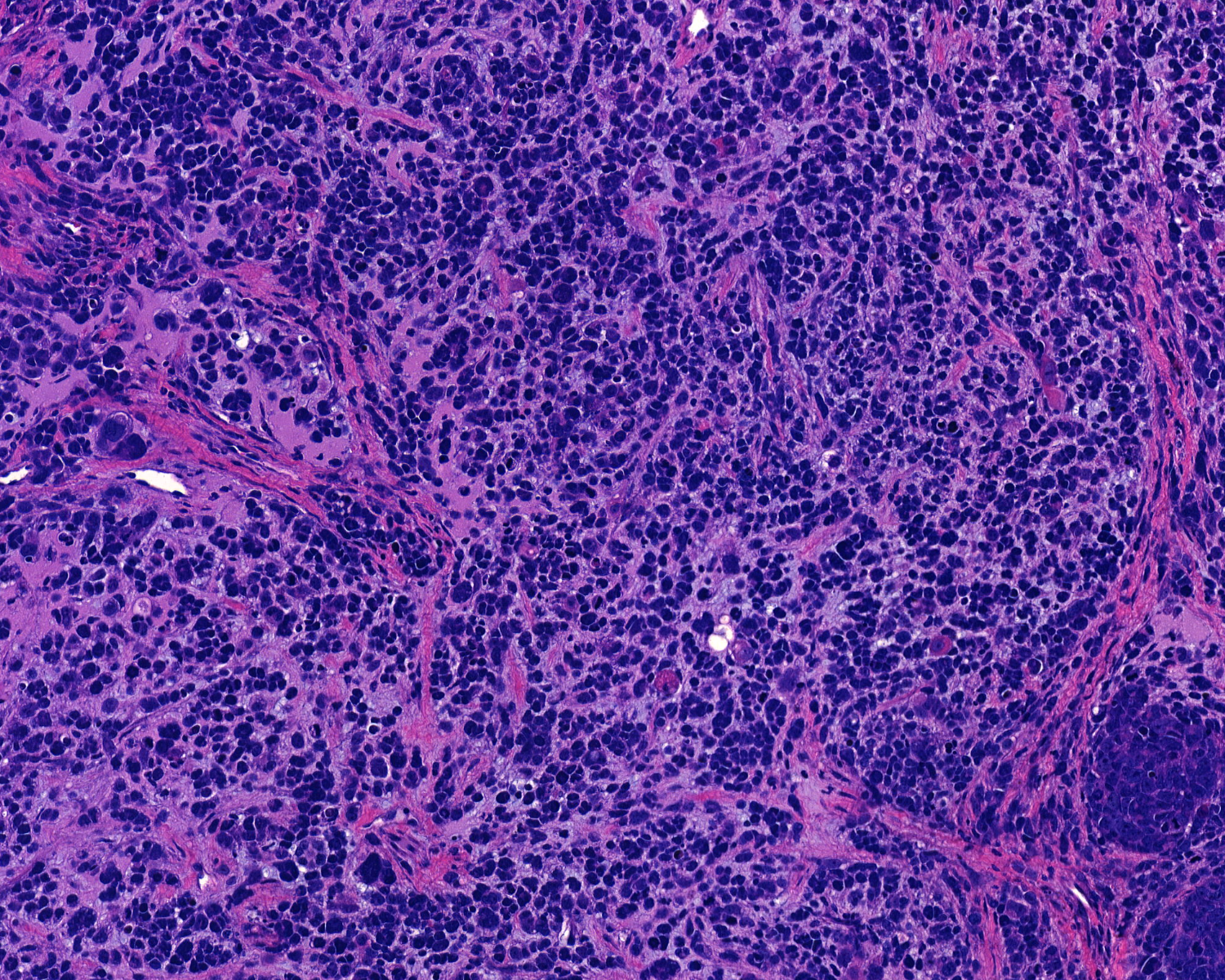

Supplement: Supplementary file 8 — Source data Fig. 3 [file 44321_2025_336_MOESM8_ESM.zip › Figure 3/Fig. 3D, H RD tumor H and E/2369 CTRL.tif]

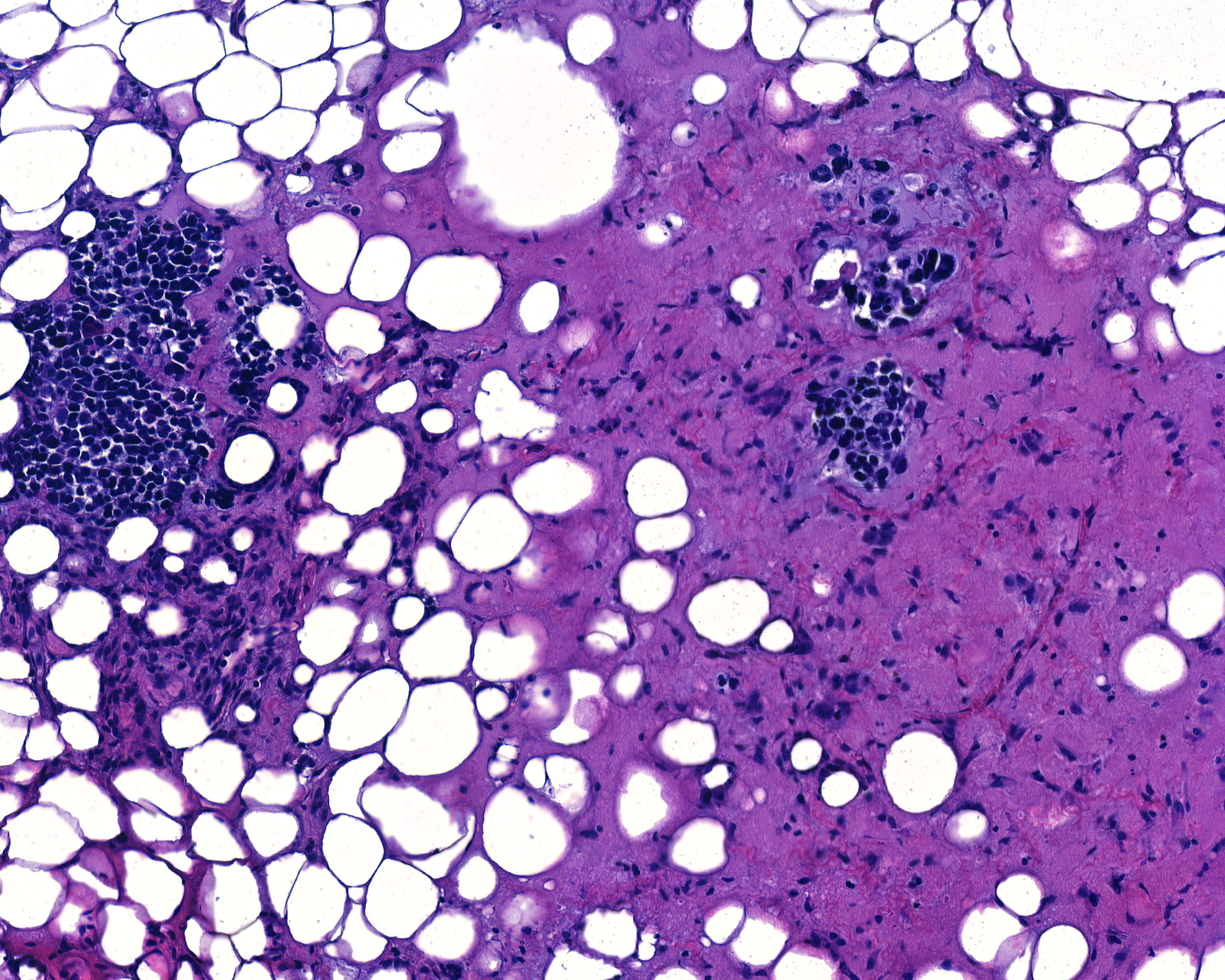

Supplement: Supplementary file 8 — Source data Fig. 3 [file 44321_2025_336_MOESM8_ESM.zip › Figure 3/Fig. 3D, H RD tumor H and E/2356 shDHCR7.tif]

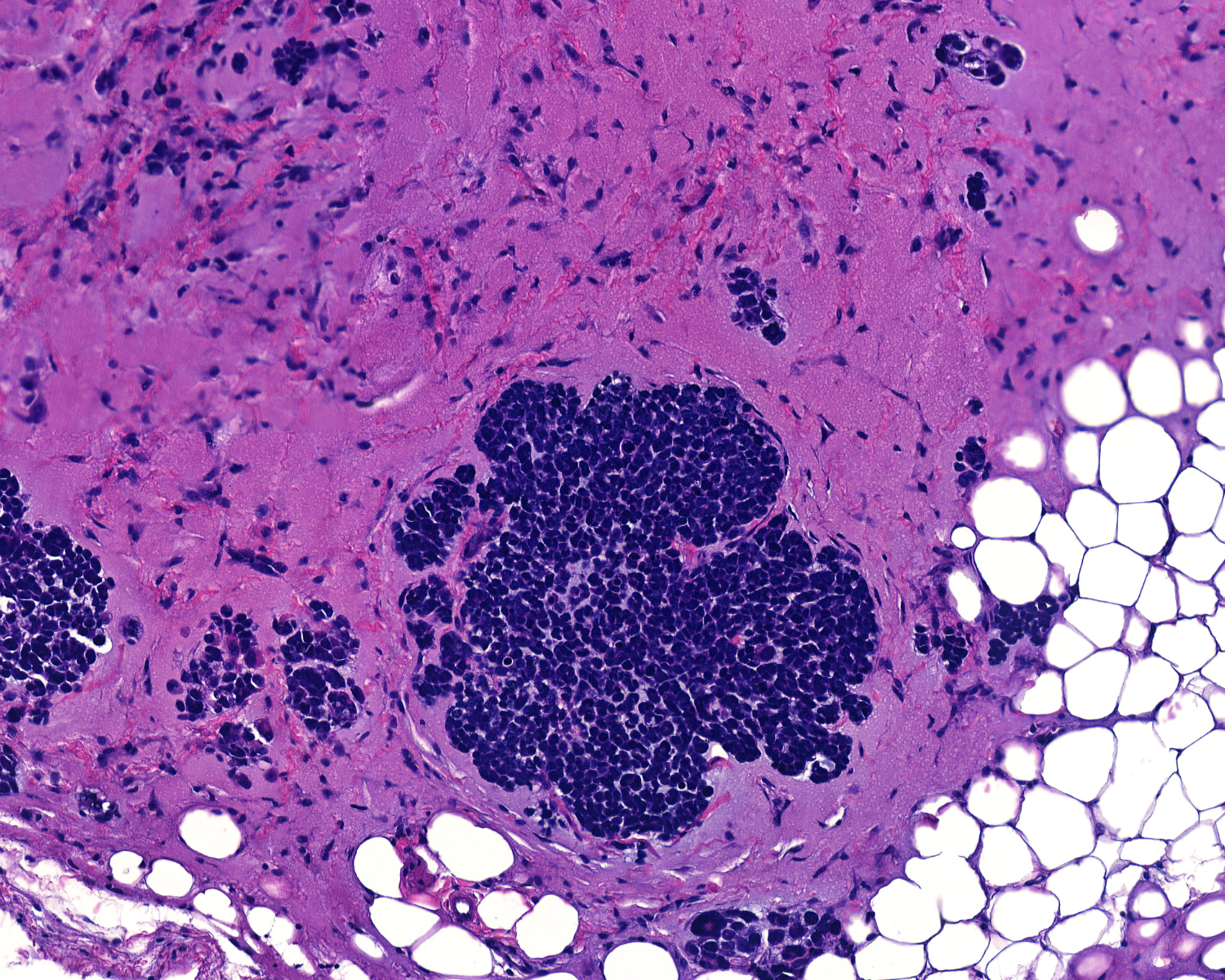

Supplement: Supplementary file 8 — Source data Fig. 3 [file 44321_2025_336_MOESM8_ESM.zip › Figure 3/Fig. 3D, H RD tumor H and E/2355 shDHCR7.tif]

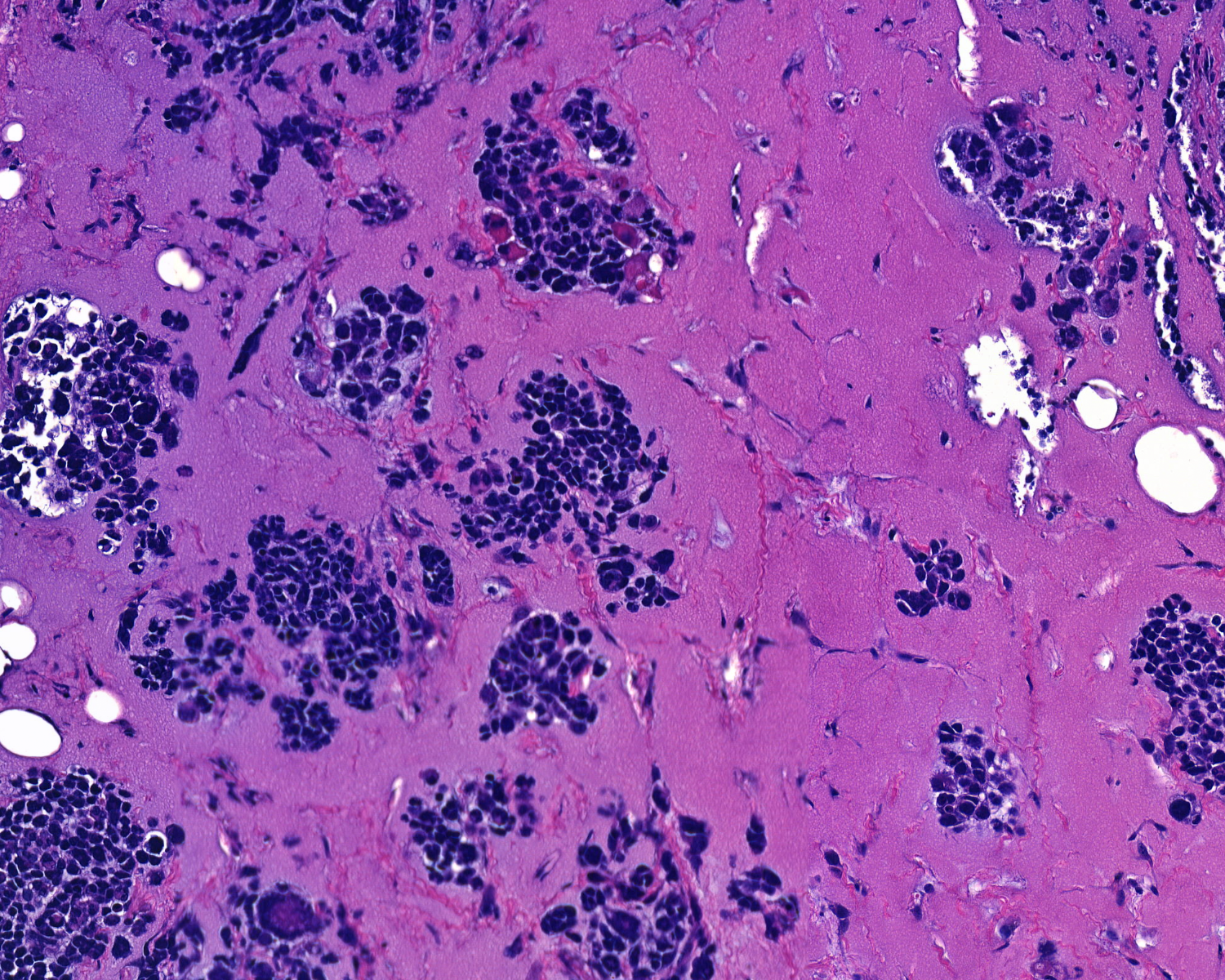

Supplement: Supplementary file 8 — Source data Fig. 3 [file 44321_2025_336_MOESM8_ESM.zip › Figure 3/Fig. 3D, H RD tumor H and E/2359 shDHCR7.tif]

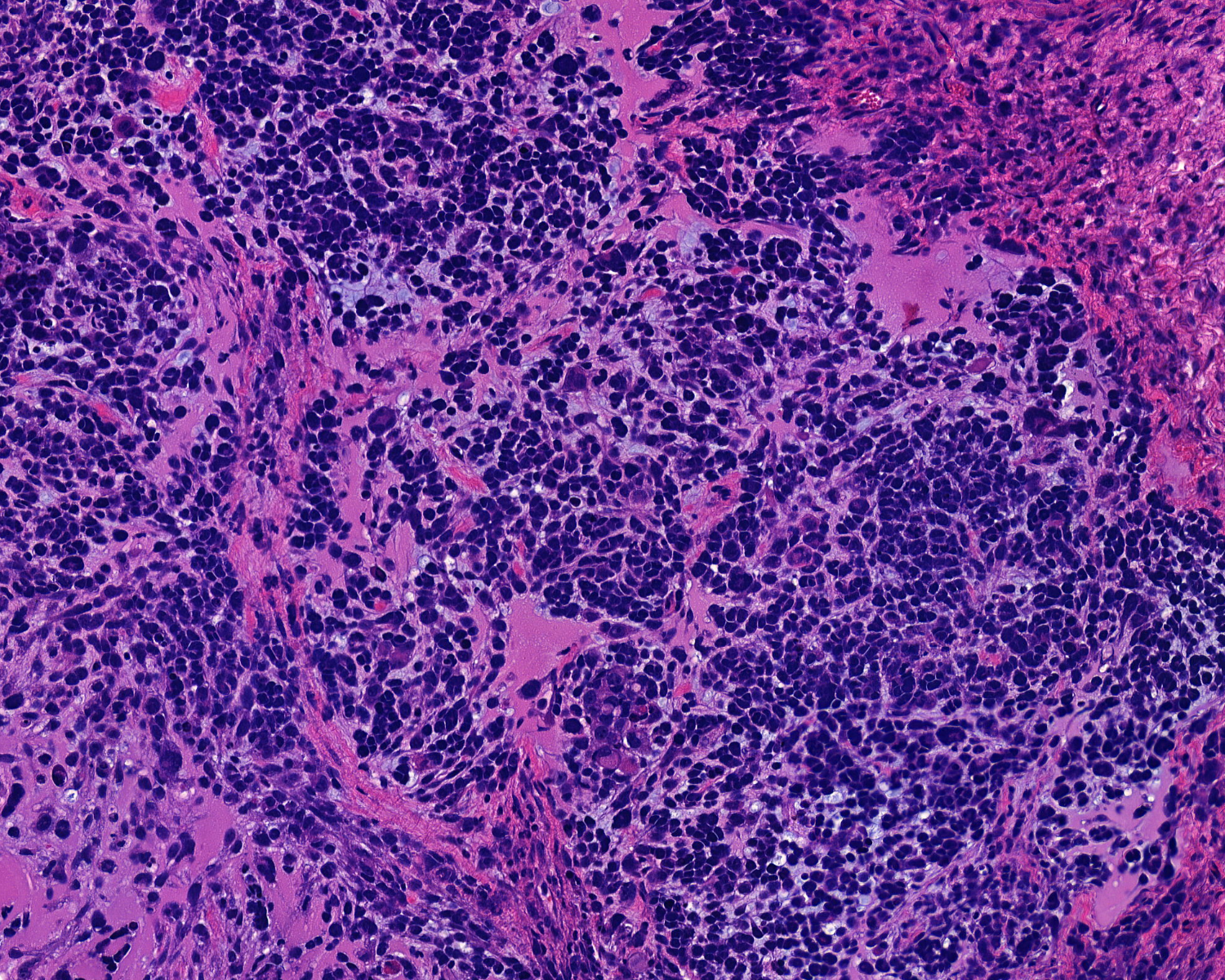

Supplement: Supplementary file 8 — Source data Fig. 3 [file 44321_2025_336_MOESM8_ESM.zip › Figure 3/Fig. 3D, H RD tumor H and E/2355 CTRL.tif]

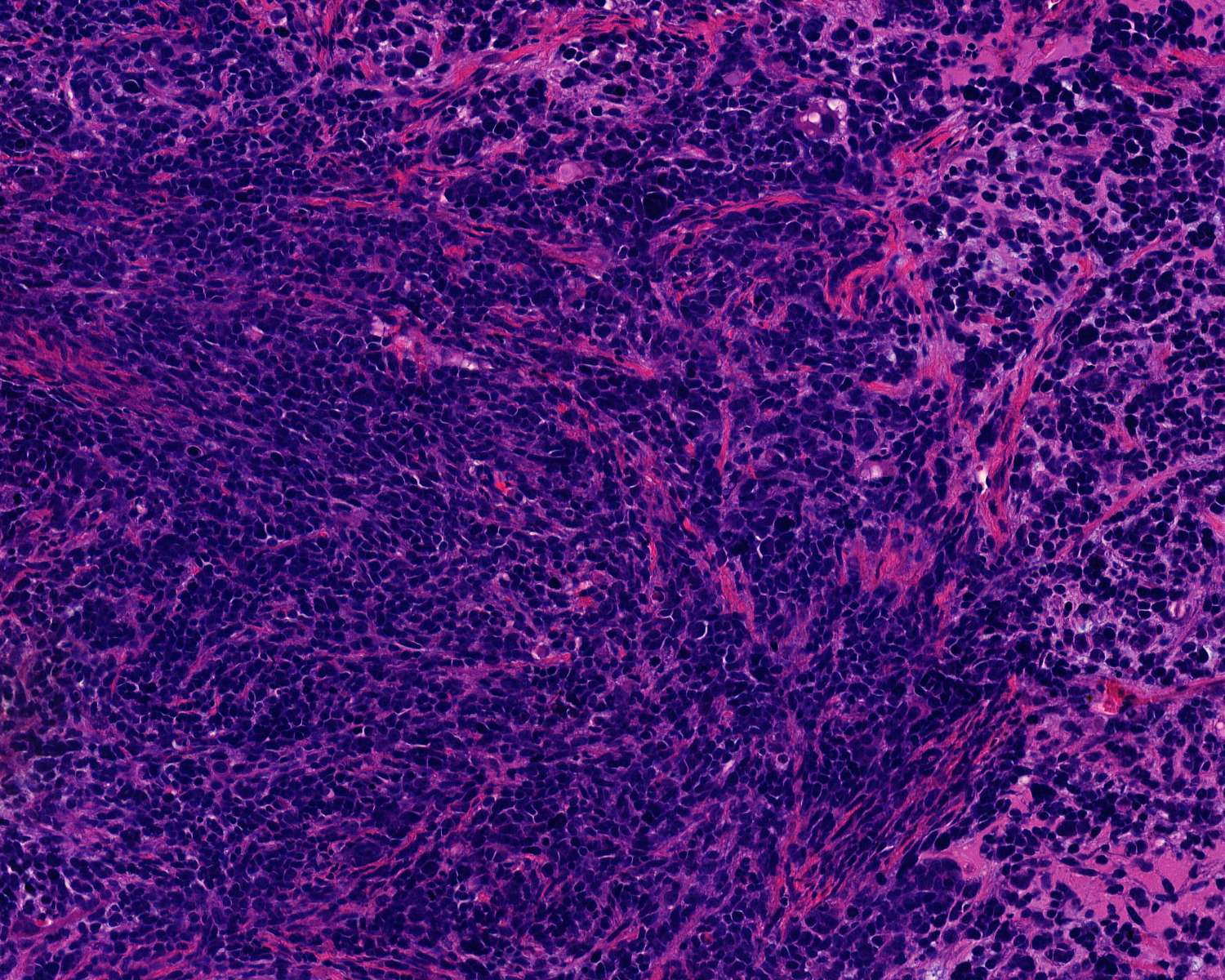

Supplement: Supplementary file 8 — Source data Fig. 3 [file 44321_2025_336_MOESM8_ESM.zip › Figure 3/Fig. 3D, H RD tumor H and E/2354 CTRL.tif]

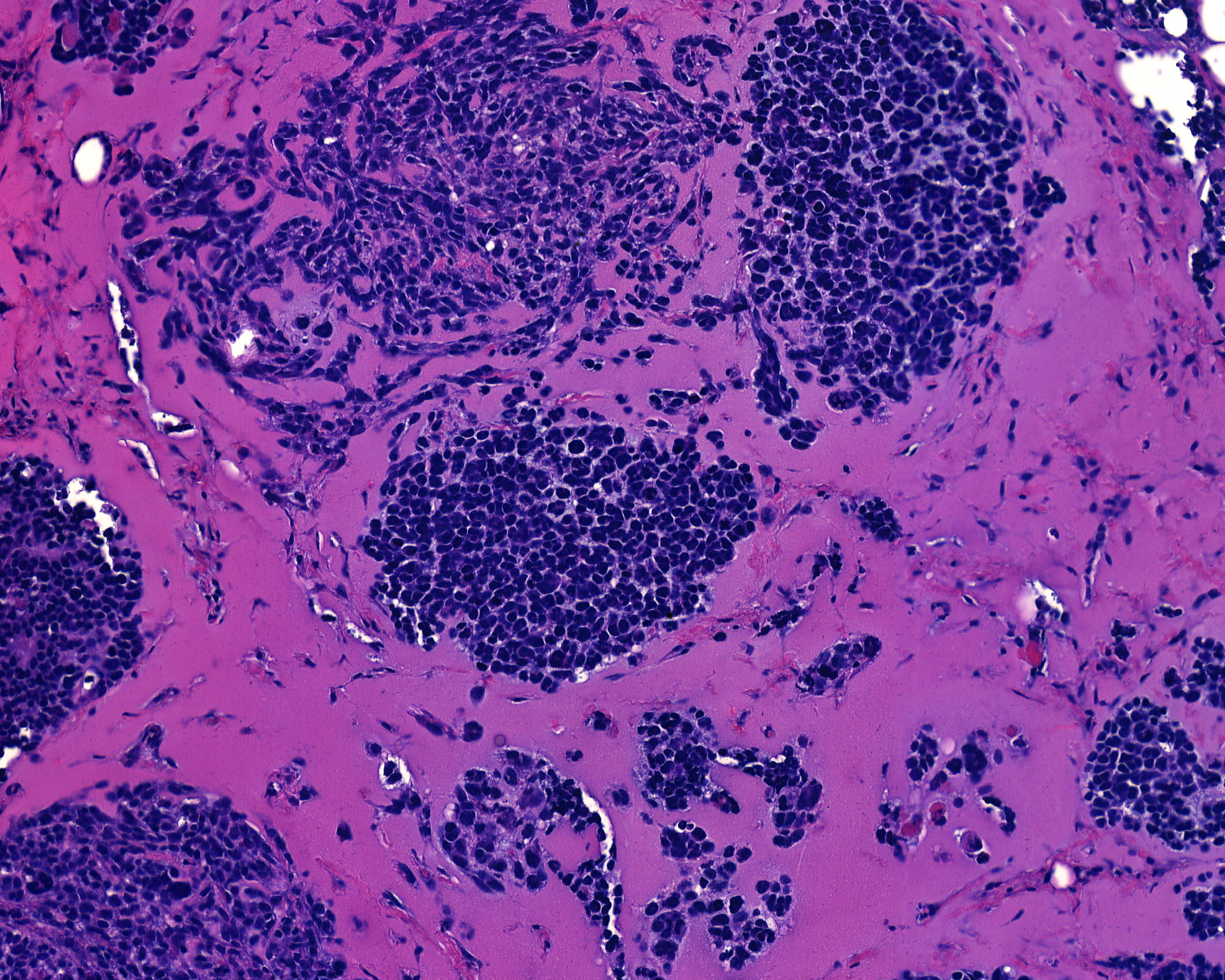

Supplement: Supplementary file 8 — Source data Fig. 3 [file 44321_2025_336_MOESM8_ESM.zip › Figure 3/Fig. 3D, H RD tumor H and E/2369 shDHCR7.tif]

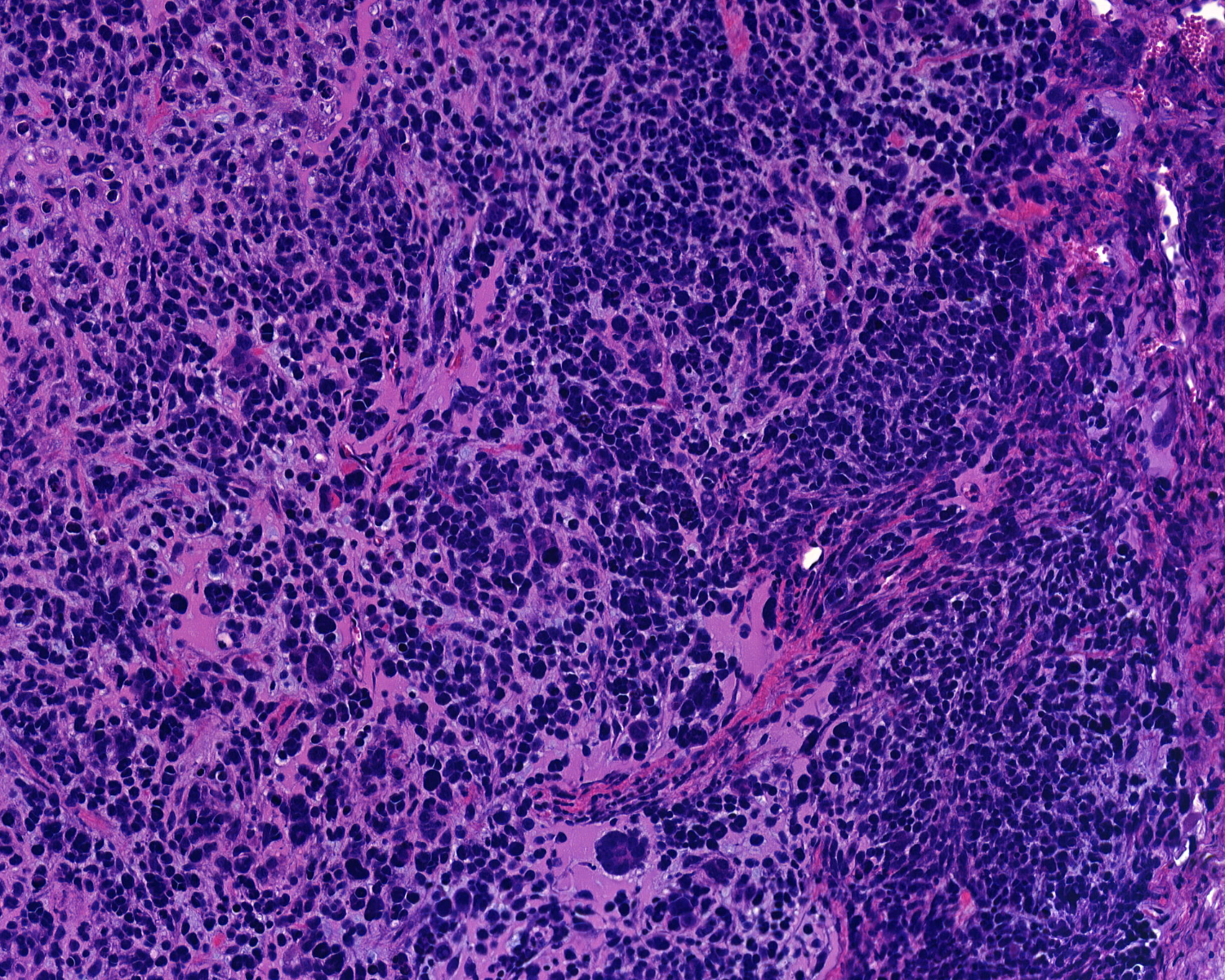

Supplement: Supplementary file 8 — Source data Fig. 3 [file 44321_2025_336_MOESM8_ESM.zip › Figure 3/Fig. 3D, H RD tumor H and E/2352 CTRL.tif]

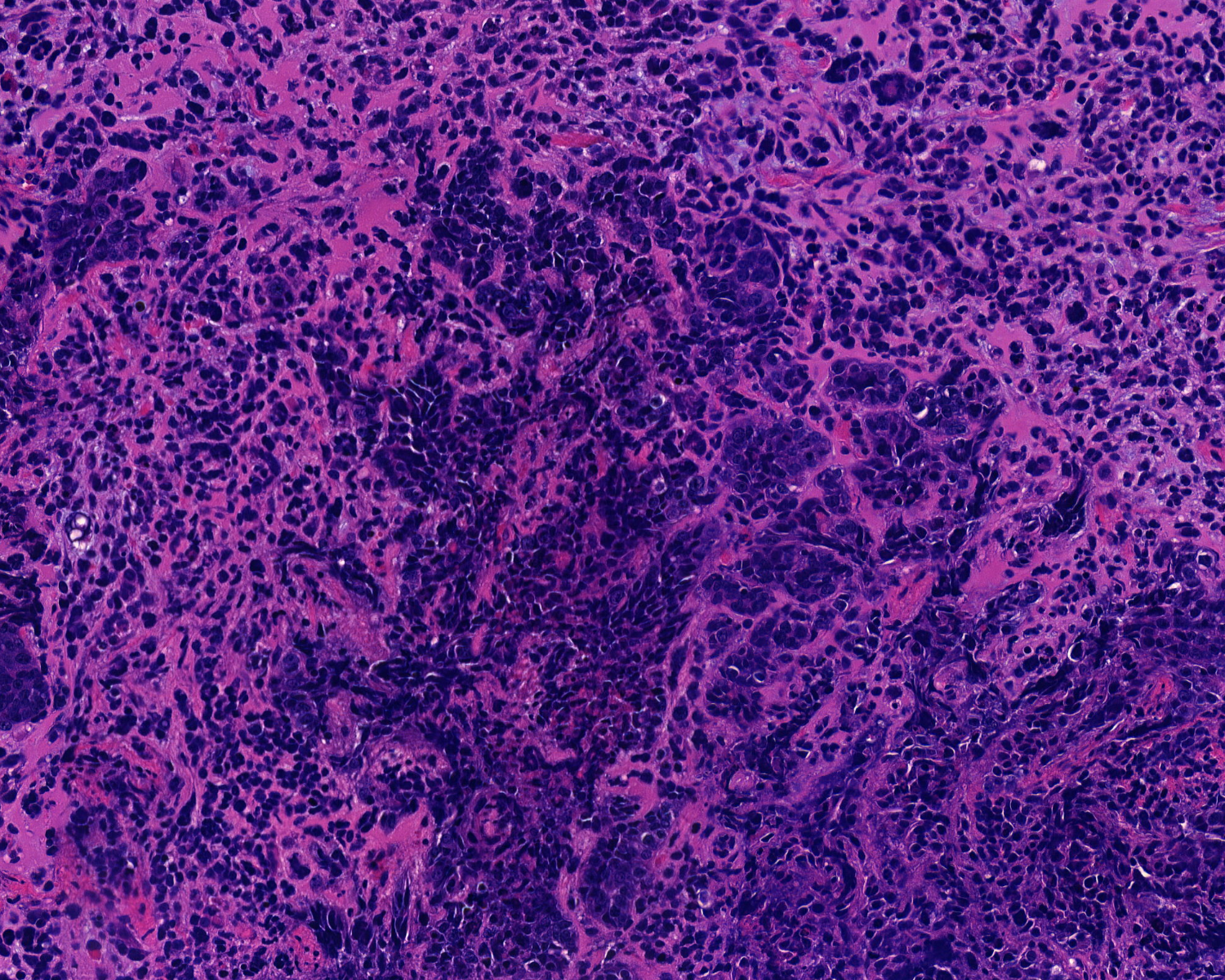

Supplement: Supplementary file 8 — Source data Fig. 3 [file 44321_2025_336_MOESM8_ESM.zip › Figure 3/Fig. 3D, H RD tumor H and E/2353 CTRL.tif]

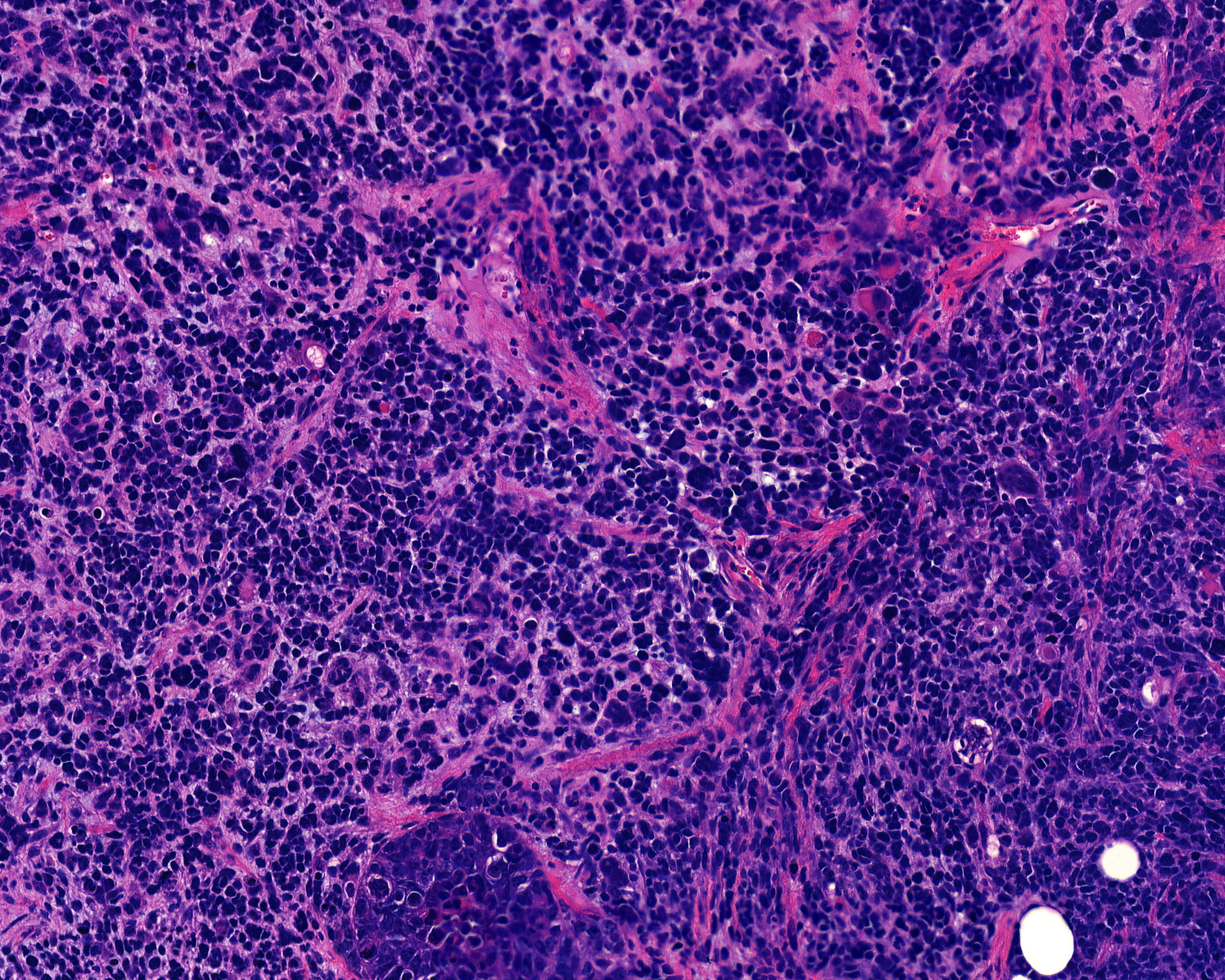

Supplement: Supplementary file 8 — Source data Fig. 3 [file 44321_2025_336_MOESM8_ESM.zip › Figure 3/Fig. 3D, H RD tumor H and E/2358 CTRL.tif]

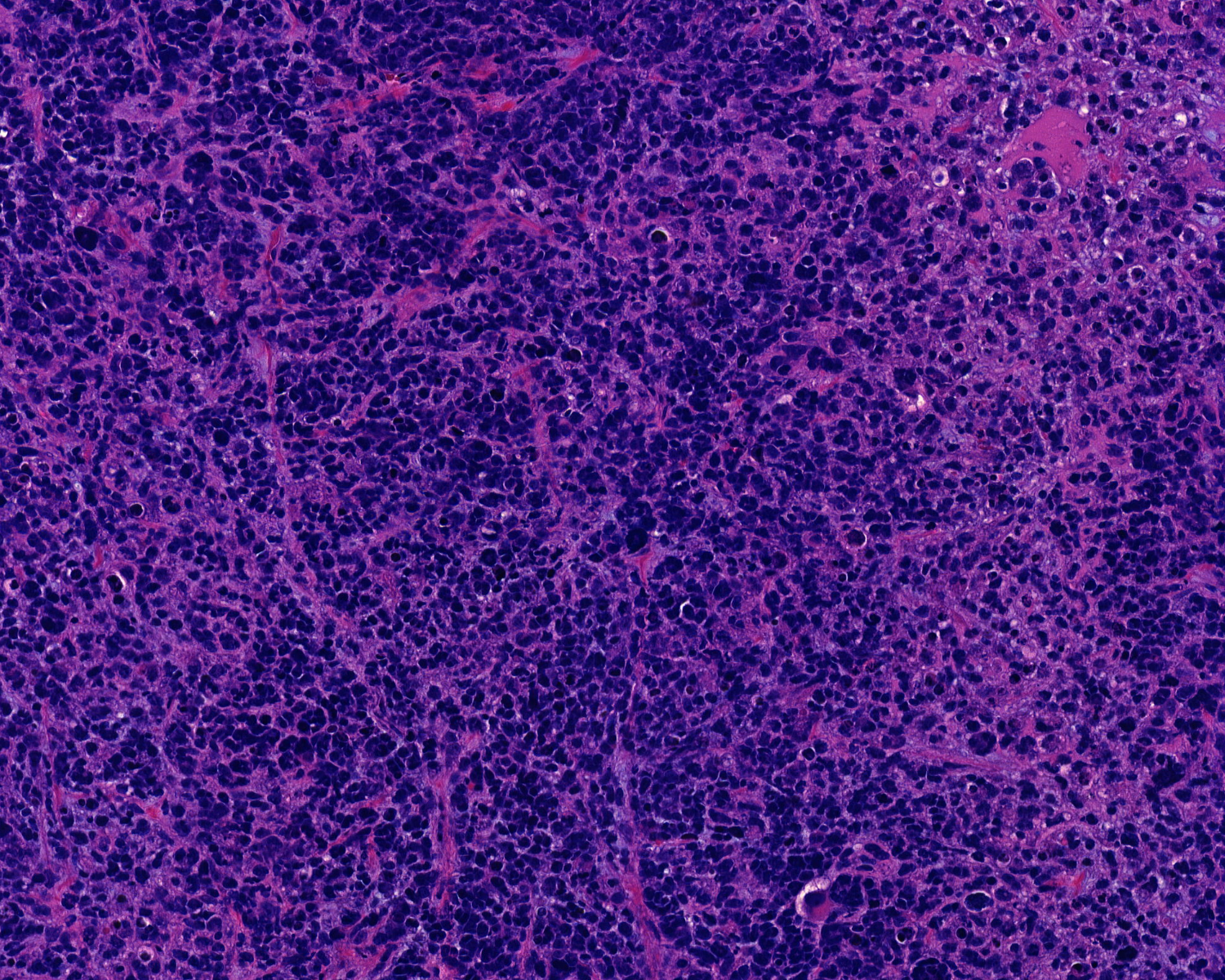

Supplement: Supplementary file 8 — Source data Fig. 3 [file 44321_2025_336_MOESM8_ESM.zip › Figure 3/Fig. 3D, H RD tumor H and E/2359 CTRL.tif]

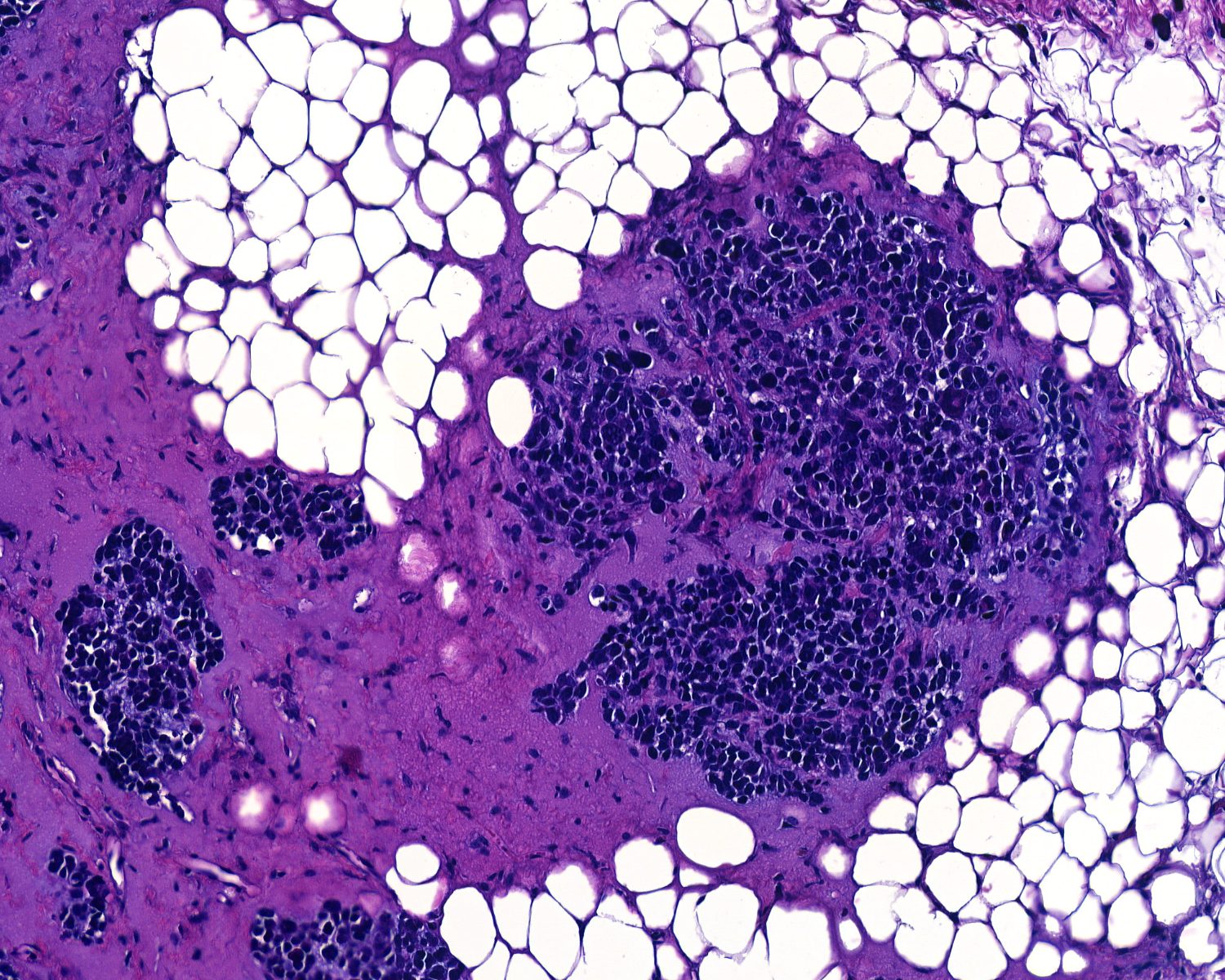

Supplement: Supplementary file 8 — Source data Fig. 3 [file 44321_2025_336_MOESM8_ESM.zip › Figure 3/Fig. 3D, H RD tumor H and E/2358 shDHCR7.tif]

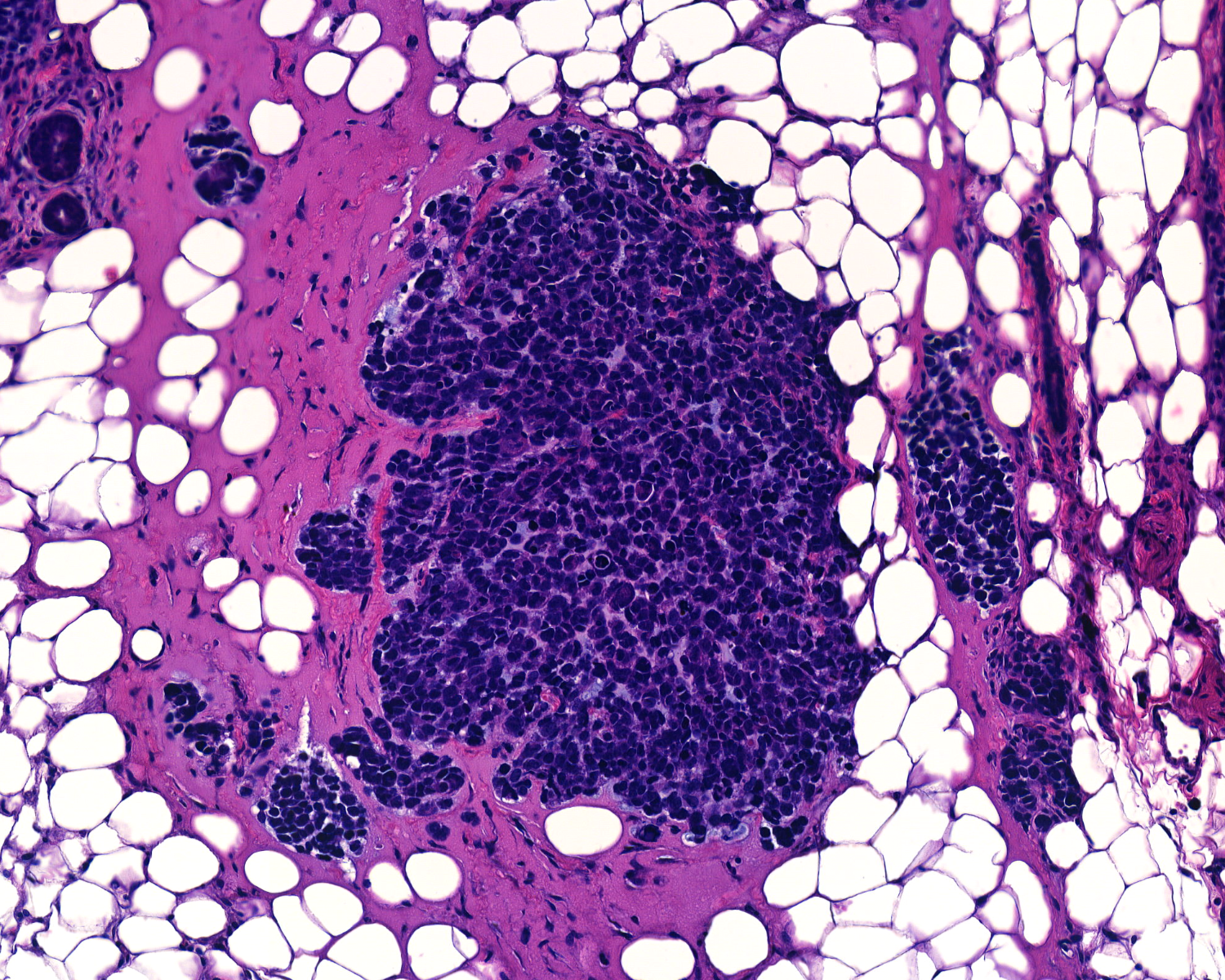

Supplement: Supplementary file 8 — Source data Fig. 3 [file 44321_2025_336_MOESM8_ESM.zip › Figure 3/Fig. 3D, H RD tumor H and E/2354 shDHCR7.tif]

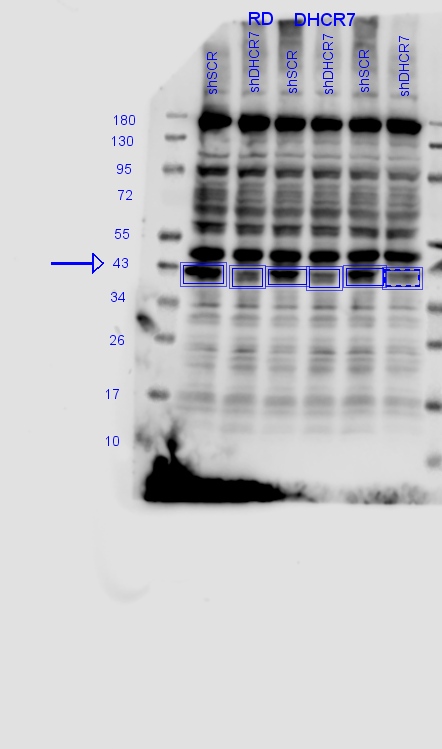

Supplement: Supplementary file 9 — Source data Fig. 4 [file 44321_2025_336_MOESM9_ESM.zip › Figure 4/Fig 4J WB RH30 RD KLHEL1 for source data/RD DHCR7.tif.tif.tif]

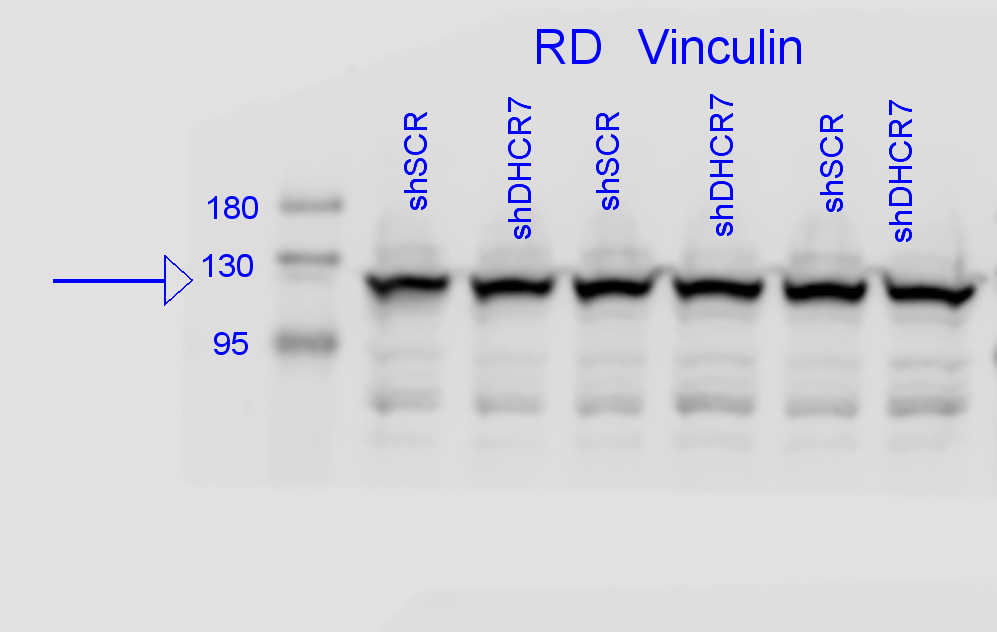

Supplement: Supplementary file 9 — Source data Fig. 4 [file 44321_2025_336_MOESM9_ESM.zip › Figure 4/Fig 4J WB RH30 RD KLHEL1 for source data/RD vincuin.tif.tif]

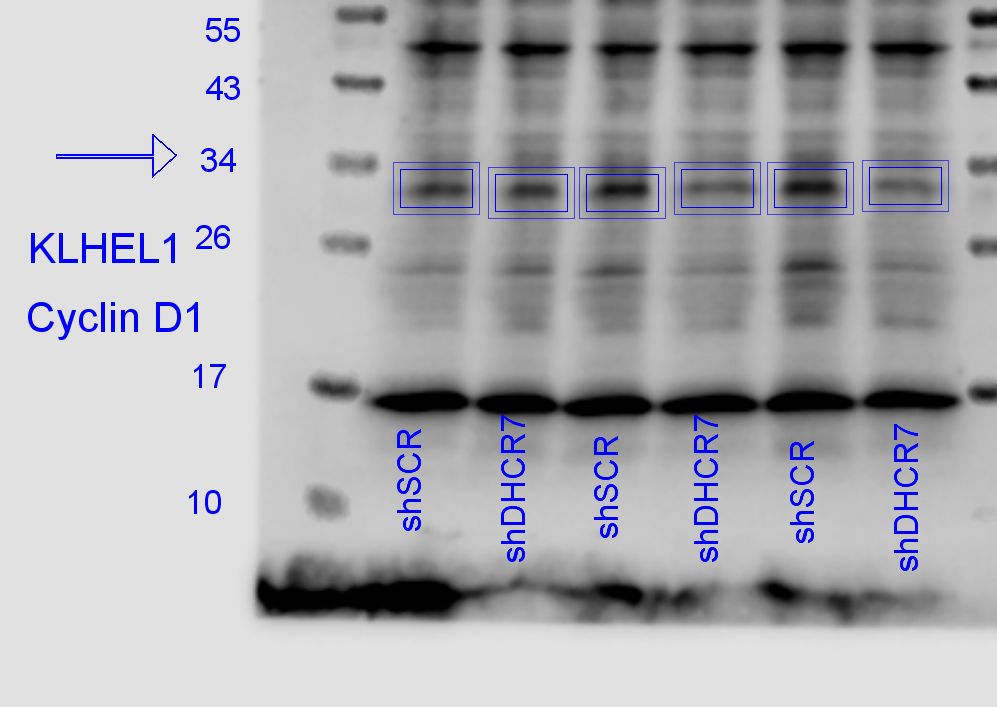

Supplement: Supplementary file 9 — Source data Fig. 4 [file 44321_2025_336_MOESM9_ESM.zip › Figure 4/Fig 4J WB RH30 RD KLHEL1 for source data/KLHEL1 Cyclin D1.tif.tif]

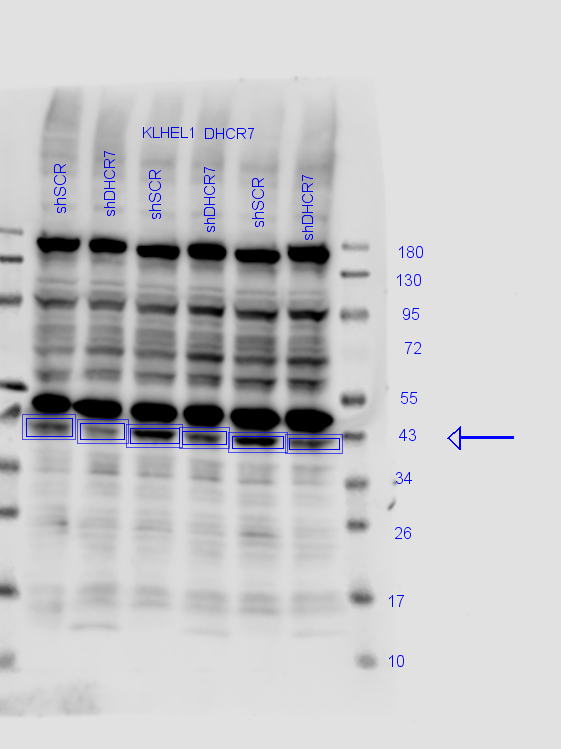

Supplement: Supplementary file 9 — Source data Fig. 4 [file 44321_2025_336_MOESM9_ESM.zip › Figure 4/Fig 4J WB RH30 RD KLHEL1 for source data/KLHEL1 DHCR7.tif]

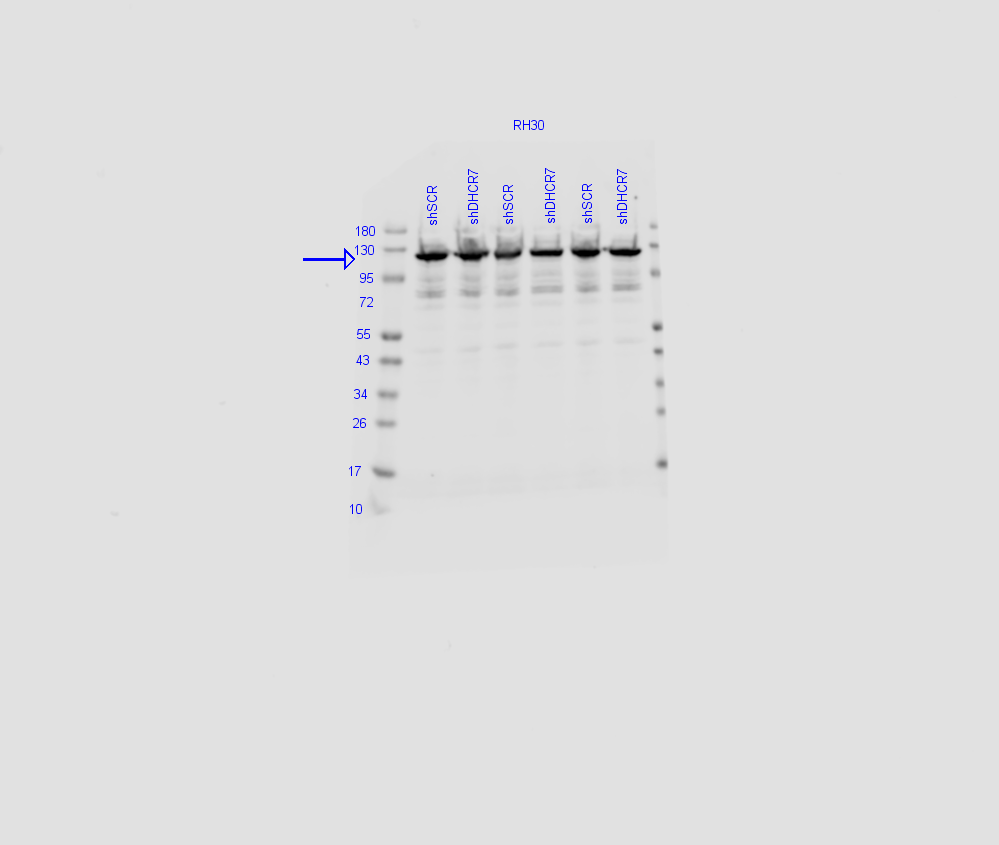

Supplement: Supplementary file 9 — Source data Fig. 4 [file 44321_2025_336_MOESM9_ESM.zip › Figure 4/Fig 4J WB RH30 RD KLHEL1 for source data/RH30 vincuin.tif]

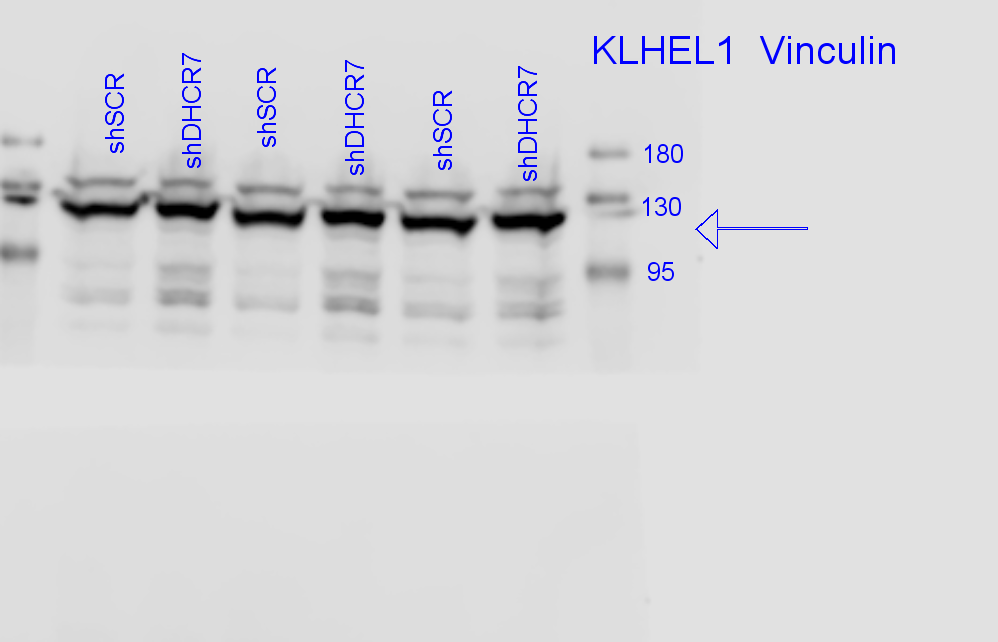

Supplement: Supplementary file 9 — Source data Fig. 4 [file 44321_2025_336_MOESM9_ESM.zip › Figure 4/Fig 4J WB RH30 RD KLHEL1 for source data/KLHEL1 vincuin.tif.tif]

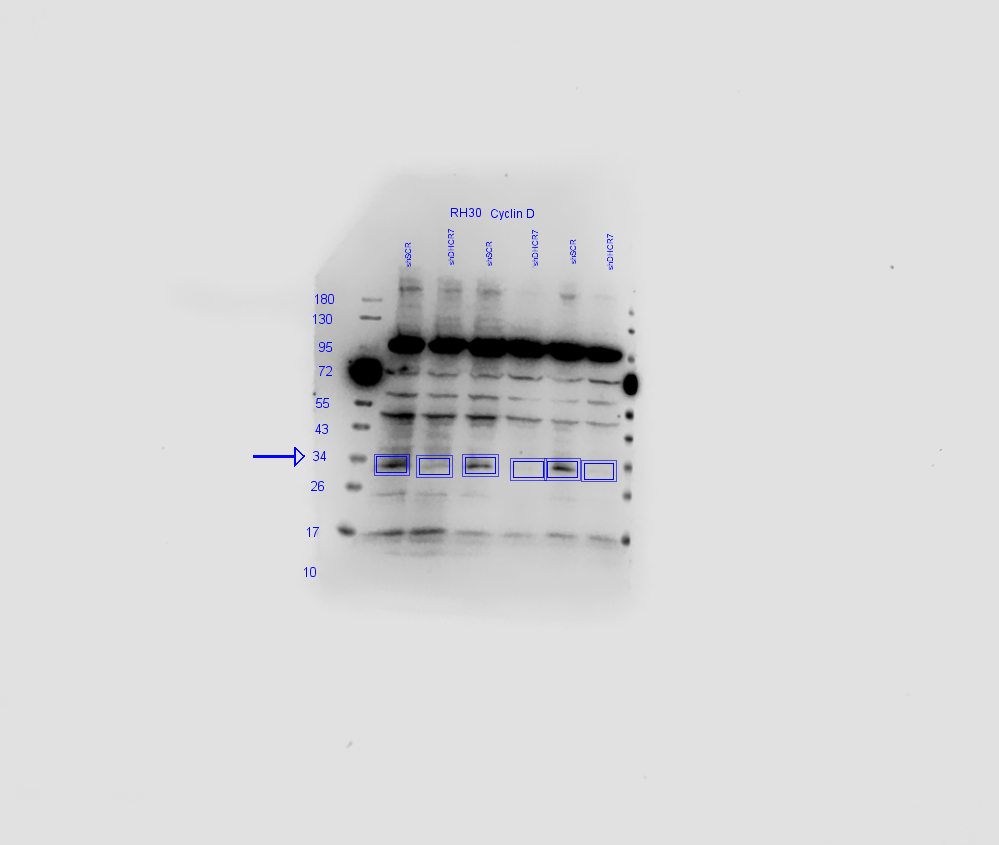

Supplement: Supplementary file 9 — Source data Fig. 4 [file 44321_2025_336_MOESM9_ESM.zip › Figure 4/Fig 4J WB RH30 RD KLHEL1 for source data/RH30 Cyclin D1.tif]

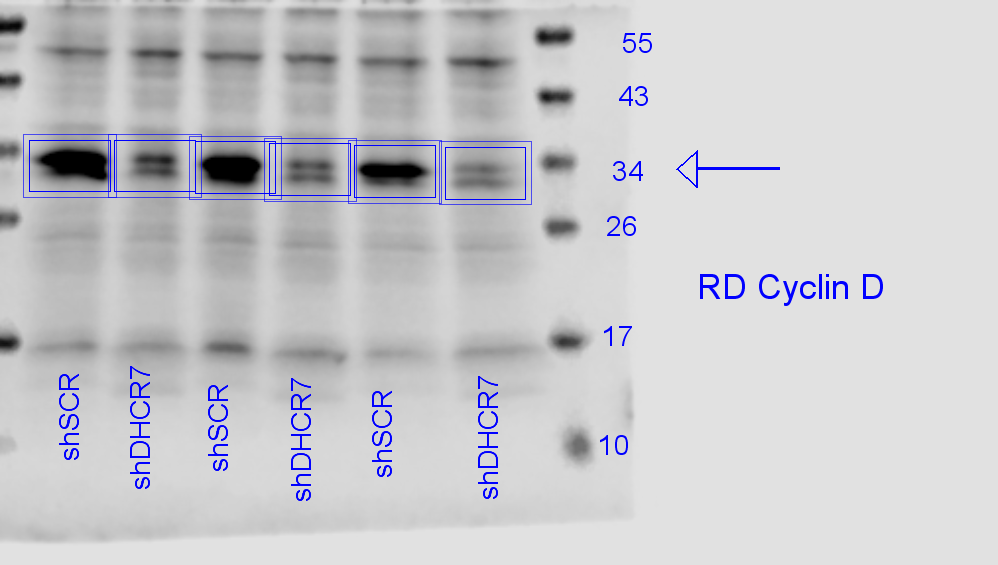

Supplement: Supplementary file 9 — Source data Fig. 4 [file 44321_2025_336_MOESM9_ESM.zip › Figure 4/Fig 4J WB RH30 RD KLHEL1 for source data/RD Cyclin D1.tif.tif]

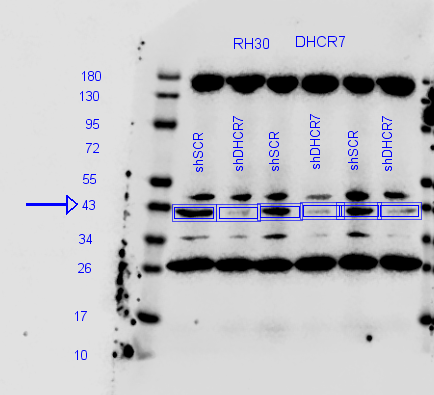

Supplement: Supplementary file 9 — Source data Fig. 4 [file 44321_2025_336_MOESM9_ESM.zip › Figure 4/Fig 4J WB RH30 RD KLHEL1 for source data/RH30 DHCR7.tif]

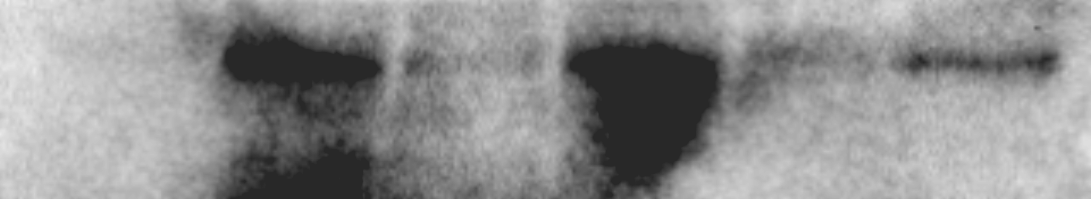

Supplement: Supplementary file 10 — Source data Fig. 5 [file 44321_2025_336_MOESM10_ESM.zip › Figure 5/Fig. 5H KLHEL1 WB UPR/280824 blot2alaviiva CHOPrewash_2.tif]

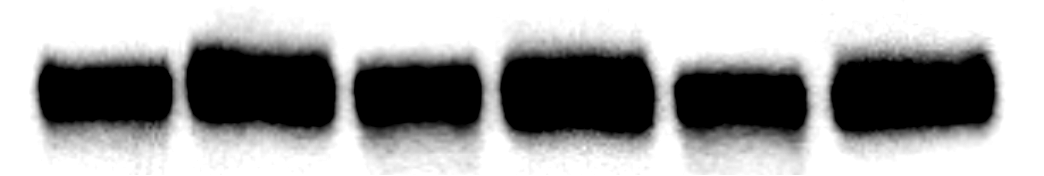

Supplement: Supplementary file 10 — Source data Fig. 5 [file 44321_2025_336_MOESM10_ESM.zip › Figure 5/Fig. 5H KLHEL1 WB UPR/280824 blot1 perk_1.tif]

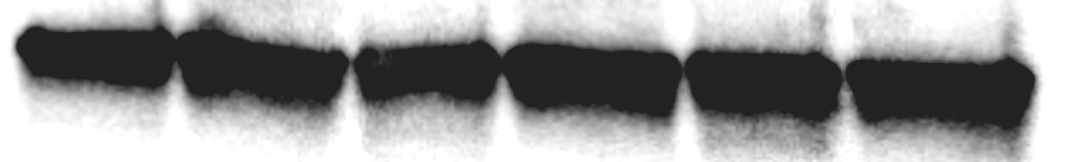

Supplement: Supplementary file 10 — Source data Fig. 5 [file 44321_2025_336_MOESM10_ESM.zip › Figure 5/Fig. 5H KLHEL1 WB UPR/280824 blot1 eif2alpha_6.tif]

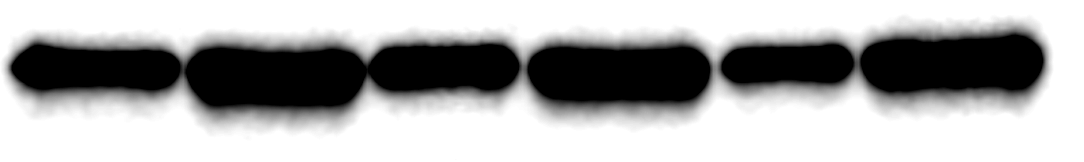

Supplement: Supplementary file 10 — Source data Fig. 5 [file 44321_2025_336_MOESM10_ESM.zip › Figure 5/Fig. 5H KLHEL1 WB UPR/280824 bpot1alaviiva peif2alpha_4.tif]

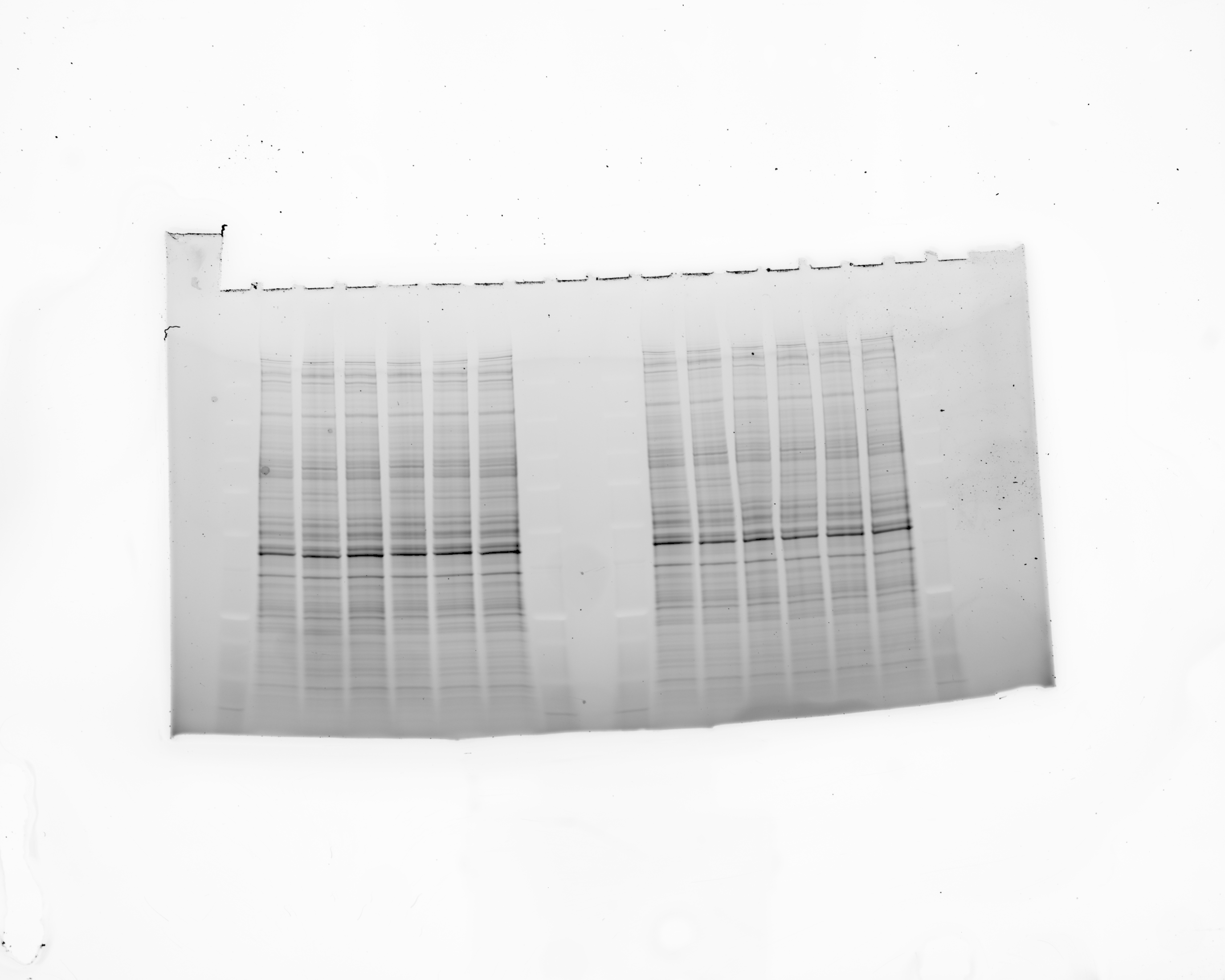

Supplement: Supplementary file 10 — Source data Fig. 5 [file 44321_2025_336_MOESM10_ESM.zip › Figure 5/Fig. 5H KLHEL1 WB UPR/090424gel1 rhabdo(Stain Free Gel).raw16.tif]

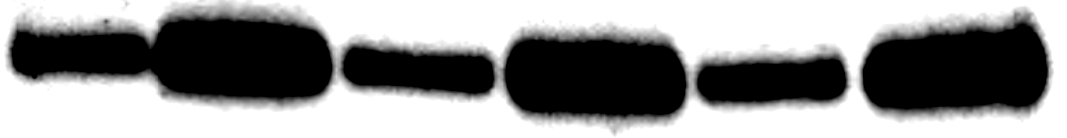

Supplement: Supplementary file 10 — Source data Fig. 5 [file 44321_2025_336_MOESM10_ESM.zip › Figure 5/Fig. 5H KLHEL1 WB UPR/280824 blot2 ATF-4_2.tif]

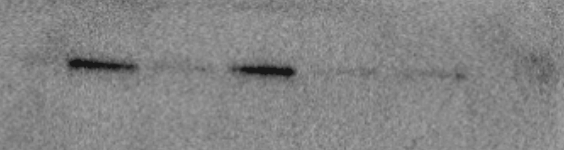

Supplement: Supplementary file 10 — Source data Fig. 5 [file 44321_2025_336_MOESM10_ESM.zip › Figure 5/Fig. 5H RD WB UPR final/100424 blot1 p-eIF2Alpha(Chemiluminescence).tif]

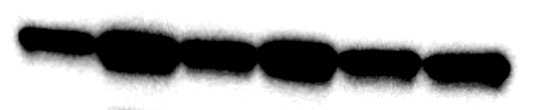

Supplement: Supplementary file 10 — Source data Fig. 5 [file 44321_2025_336_MOESM10_ESM.zip › Figure 5/Fig. 5H RD WB UPR final/090424 blot1 eIF2Alpha_2(Chemiluminescence).tif]

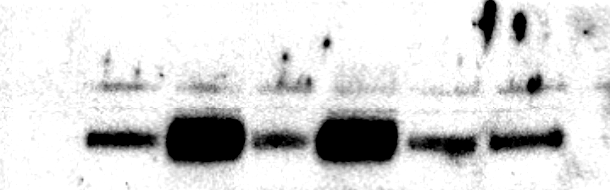

Supplement: Supplementary file 10 — Source data Fig. 5 [file 44321_2025_336_MOESM10_ESM.zip › Figure 5/Fig. 5H RD WB UPR final/090424 blot1 ATF-4_1(Chemiluminescence).tif]

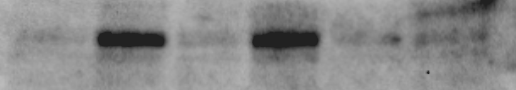

Supplement: Supplementary file 10 — Source data Fig. 5 [file 44321_2025_336_MOESM10_ESM.zip › Figure 5/Fig. 5H RD WB UPR final/090424 blot1 CHOP_2(Chemiluminescence).tif]

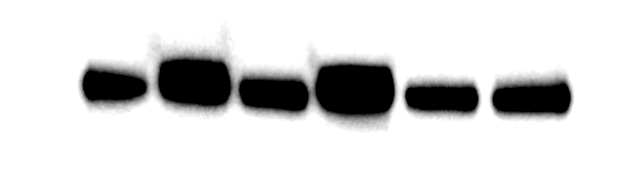

Supplement: Supplementary file 10 — Source data Fig. 5 [file 44321_2025_336_MOESM10_ESM.zip › Figure 5/Fig. 5H RD WB UPR final/090424 blot1 PERK_4(Chemiluminescence).tif]
