# Supplementary material for: Inhibiting cholesterol synthesis halts rhabdomyosarcoma growth via ER stress and cell cycle arrest
Source: EMBO Mol Med. 2025 Nov 17;17(12):3586–606. doi: 10.1038/s44321-025-00336-x (PMC12686467; doi:10.1038/s44321-025-00336-x)
Supplement: Supplementary file 9 — Source data Fig. 4 [file 44321_2025_336_MOESM9_ESM.zip › Figure 4/Fig. 4L RH30 cell cycle shDHCR7 D2.pdf]

Report of Specimen1

Specimen Name: Specimen1 | Run Time: 7/30/2025 2:32 PM

Cytometer: NovoCyte Quanteon 621210411873 | Software: NovoExpress 1.6.2

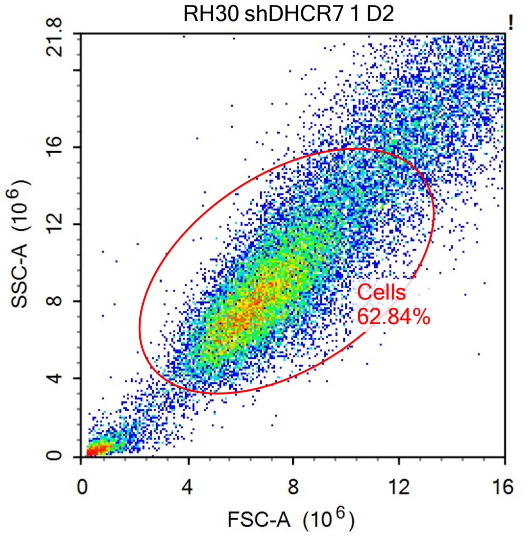

Gate	Count	% All	Median X	Median Y
All	29,153	100.00%	8,160,793	10,186,928
Cells	18,320	62.84%	7,194,767	8,784,592

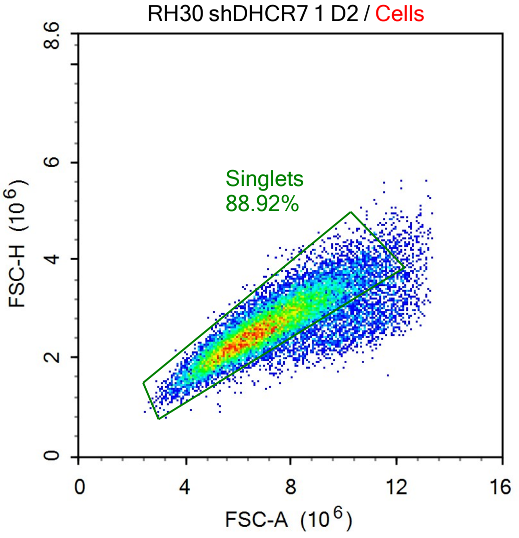

Gate	Count	% Cells	Median X	Median Y
Cells	18,320	100.00%	7,194,767	2,638,662
Singlet	16,290	88.92%	6,931,587	2,618,239

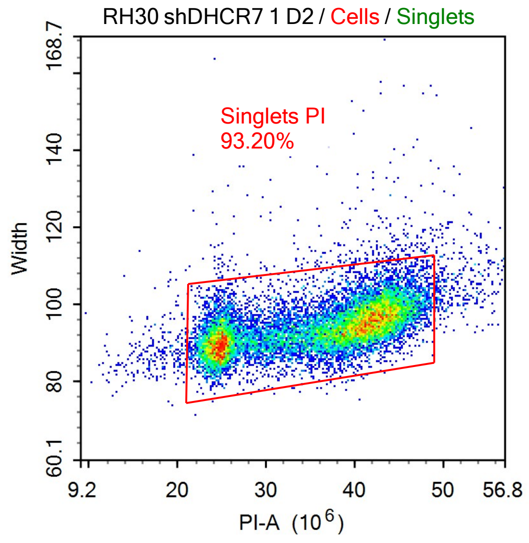

Gate	Count	% Singlets	Median X	Median Y
Singlets	16,290	100.00%	38,130,464	94
Singlets PI	15,182	93.20%	37,858,736	94

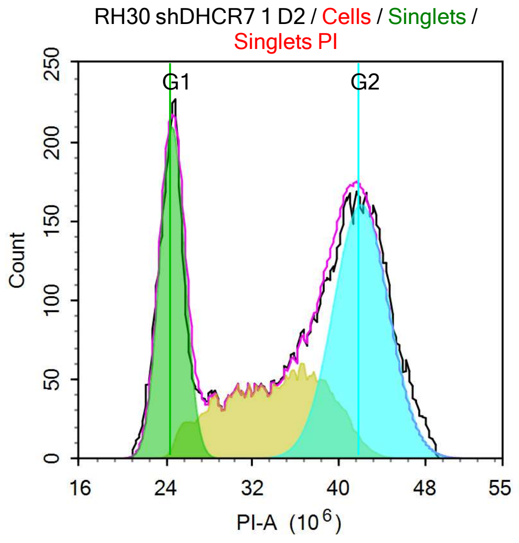

RMS	Freq G1	Freq S	Freq G2	G2/G1	CV G1
5.18	26.30	29.50	44.22	1.71	4.72%

RH30 shDHCR7 1 D2 / Cells / Singlets /
Singlets PI

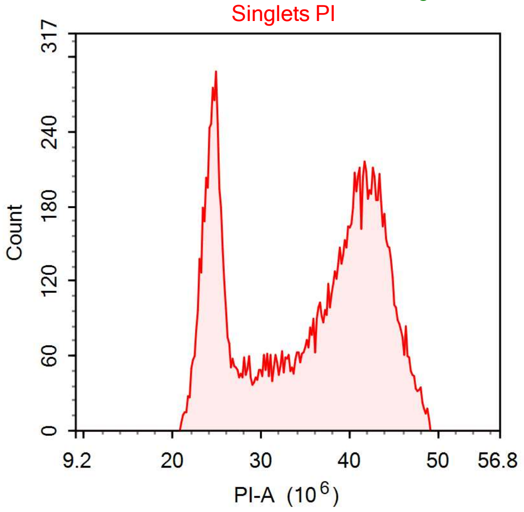

Gate	Count	% Singlets PI	Median X
Singlets PI	15,182	100.00%	37,858,736

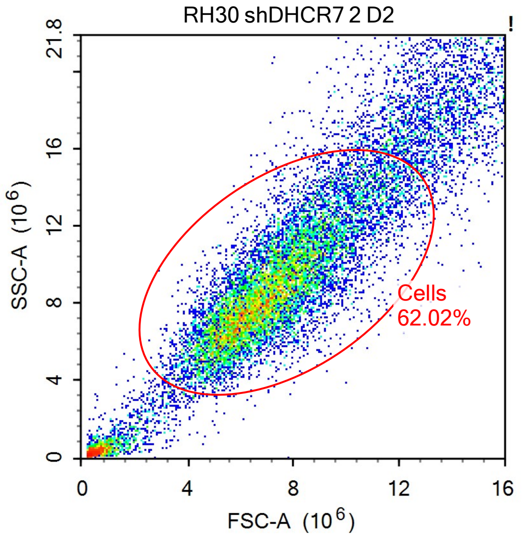

Gate	Count	% All	Median X	Median Y
All	18,544	100.00%	7,960,461	9,815,339
Cells	11,501	62.02%	7,217,689	8,685,506

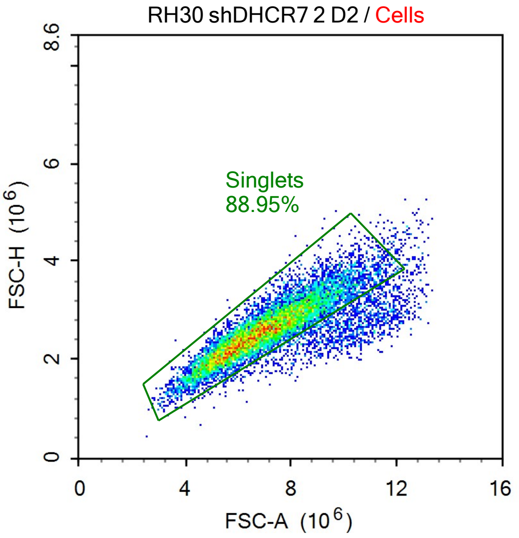

Gate	Count	% Cells	Median X	Median Y
Cells	11,501	100.00%	7,217,689	2,625,753
Singlet	10,230	88.95%	6,960,038	2,606,091

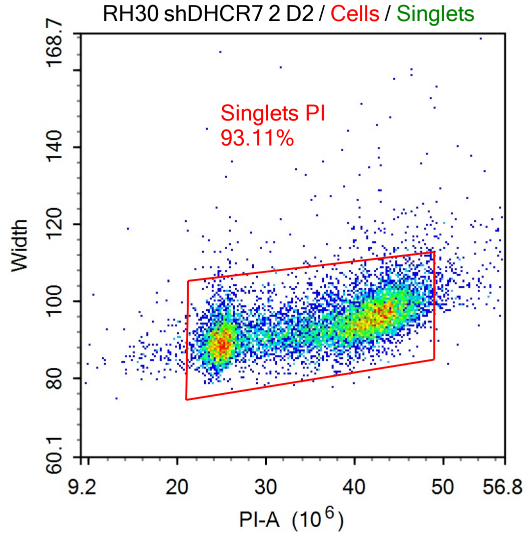

Gate	Count	% Singlets	Median X	Median Y
Singlets	10,230	100.00%	38,248,500	94
Singlets PI	9,525	93.11%	37,913,692	94

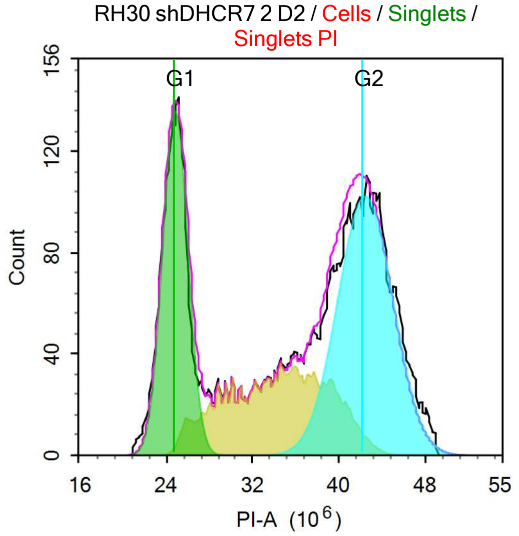

RMS	Freq G1	Freq S	Freq G2	G2/G1	CV G1
3.35	26.96	28.83	44.22	1.71	4.71%

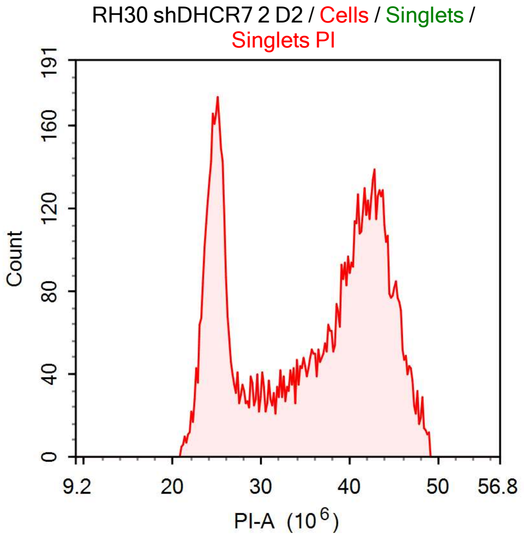

Gate	Count	% Singlets PI	Median X
Singlets PI	9,525	100.00%	37,913,692

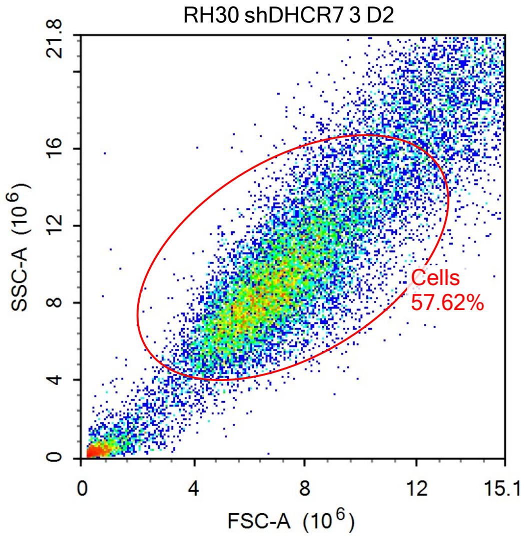

Gate	Count	% All	Median X	Median Y
All	24,602	100.00%	8,092,712	10,712,822
Cells	14,176	57.62%	7,202,614	9,298,842

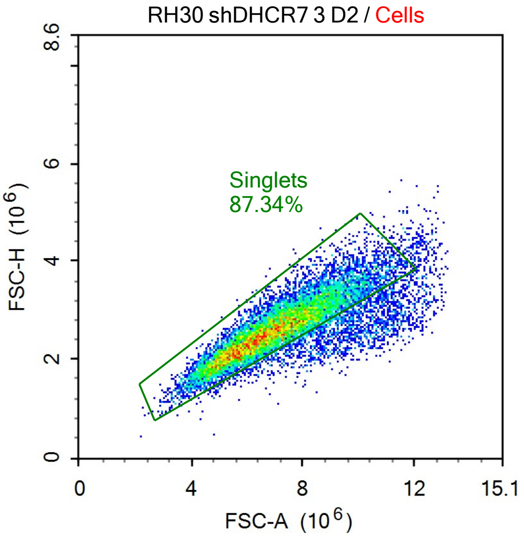

Gate	Count	% Cells	Median X	Median Y
Cells	14,176	100.00%	7,202,614	2,633,147
Singlet	12,382	87.34%	6,922,334	2,617,431

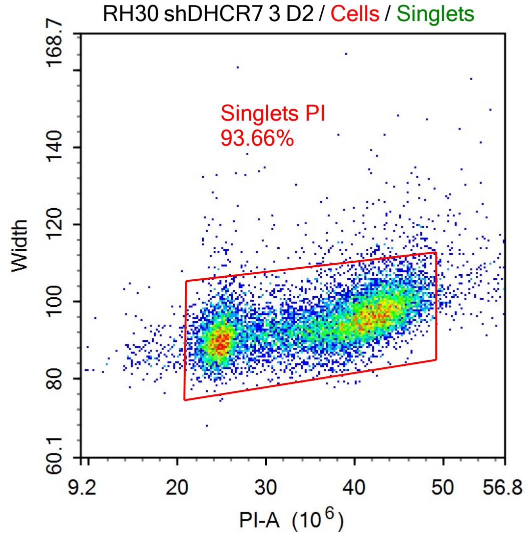

Gate	Count	% Singlets	Median X	Median Y
Singlets	12,382	100.00%	37,962,060	95
Singlets PI	11,597	93.66%	37,742,484	94

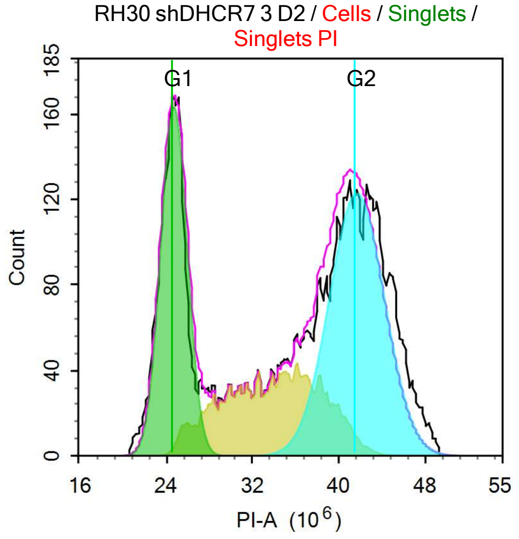

RMS	Freq G1	Freq S	Freq G2	G2/G1	CV G1
6.16	28.47	26.88	44.67	1.69	4.94%

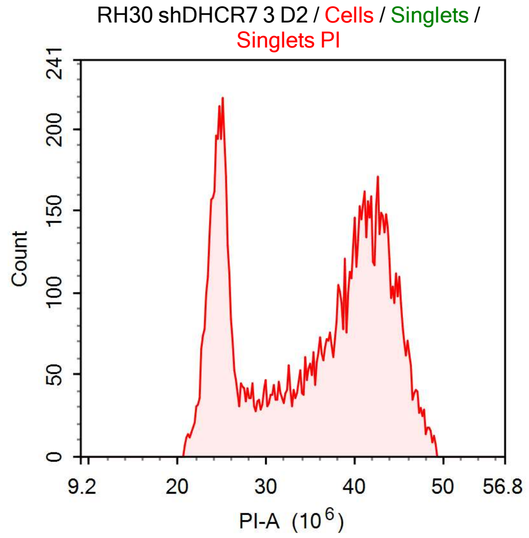

Gate	Count	% Singlets PI	Median X
Singlets PI	11,597	100.00%	37,742,484
