# Supplementary material for: Inhibiting cholesterol synthesis halts rhabdomyosarcoma growth via ER stress and cell cycle arrest
Source: EMBO Mol Med. 2025 Nov 17;17(12):3586–606. doi: 10.1038/s44321-025-00336-x (PMC12686467; doi:10.1038/s44321-025-00336-x)
Supplement: Supplementary file 9 — Source data Fig. 4 [file 44321_2025_336_MOESM9_ESM.zip › Figure 4/Fig. 4L RD shDHCR7 D1.pdf]

Report of shDHCR7-RD shDHCR7 D1

Sample Name: shDHCR7-RD shDHCR7 D1 — Run Time: 7/30/2025 2:05 PM

Cytometer: NovoCyte Quanteon 621210411873 — Software: NovoExpress 1.6.2

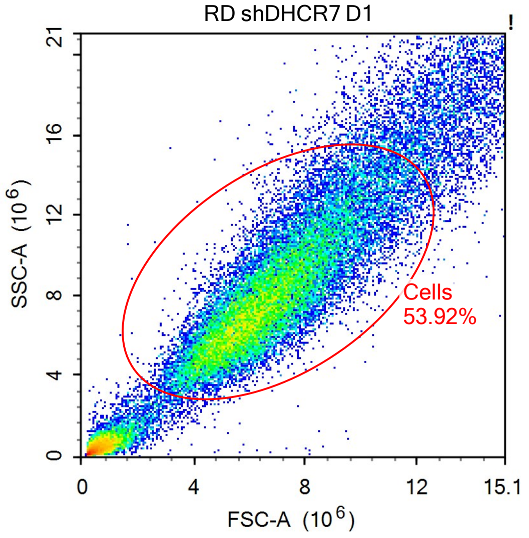

Gate	Count	% All	Median X	Median Y
All	46,010	100.00%	6,776,207	7,981,425
Cells	24,807	53.92%	6,786,985	7,991,753

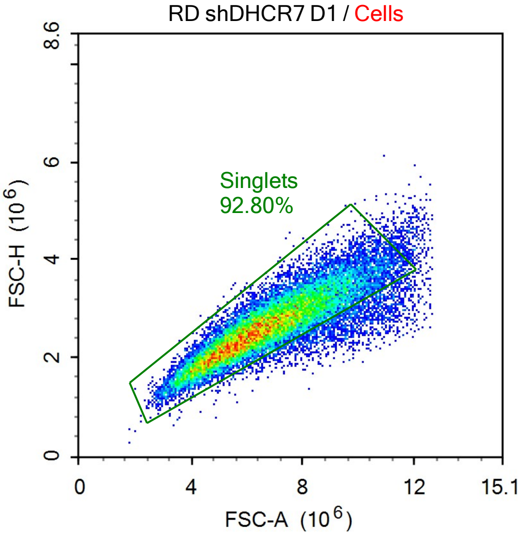

Gate	Count	% Cells	Median X	Median Y
Cells	24,807	100.00%	6,786,985	2,663,372
Singlet[illegible]	23,020	92.80%	6,608,591	2,649,058

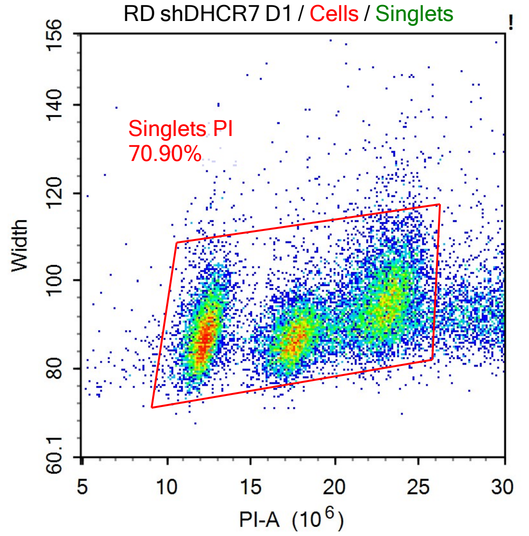

Gate	Count	% Singlets	Median X	Median Y
Singlets	23,020	100.00%	21,497,284	92
Singlets [illegible]	16,321	70.90%	17,928,148	90

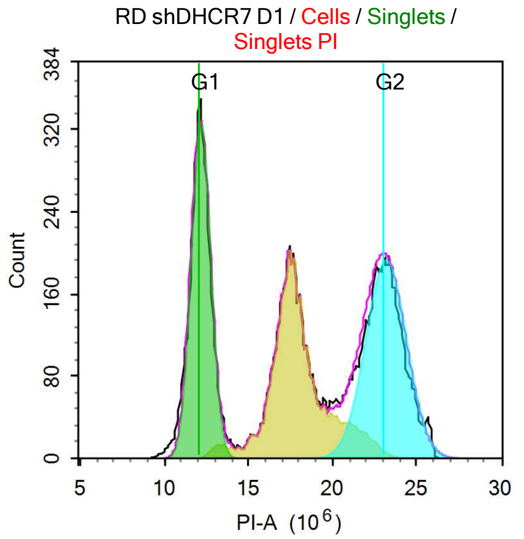

RMS	Freq G1	Freq S	Freq G2	G2/G1	CV G1
4.94	29.07	34.86	35.90	1.90	4.75%

RD shDHCR7 D1 / Cells / Singlets /
Singlets PI

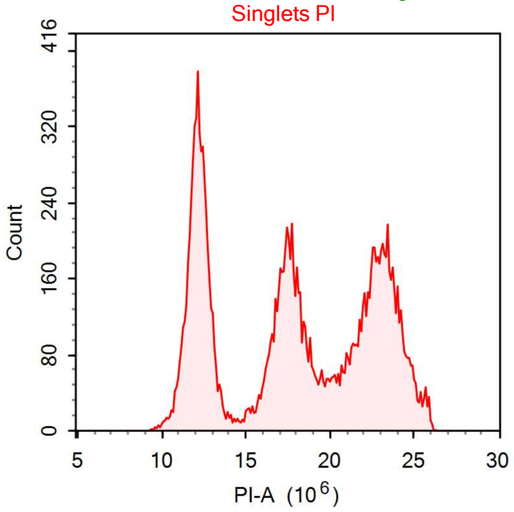

Gate	Count	% Singlets PI	Median X
Singlets [illegible]	16,321	100.00%	17,928,148
