# Supplementary material for: Inhibiting cholesterol synthesis halts rhabdomyosarcoma growth via ER stress and cell cycle arrest
Source: EMBO Mol Med. 2025 Nov 17;17(12):3586–606. doi: 10.1038/s44321-025-00336-x (PMC12686467; doi:10.1038/s44321-025-00336-x)
Supplement: Supplementary file 9 — Source data Fig. 4 [file 44321_2025_336_MOESM9_ESM.zip › Figure 4/Fig. 4L RD shSCR.pdf]

Report of Cell Cycle

Specimen Name: PI staining

Run Time: 9/18/2024 11:20 AM

Cytometer: NovoCyte Quanteon 621210411873

Software: NovoExpress 1.6.2

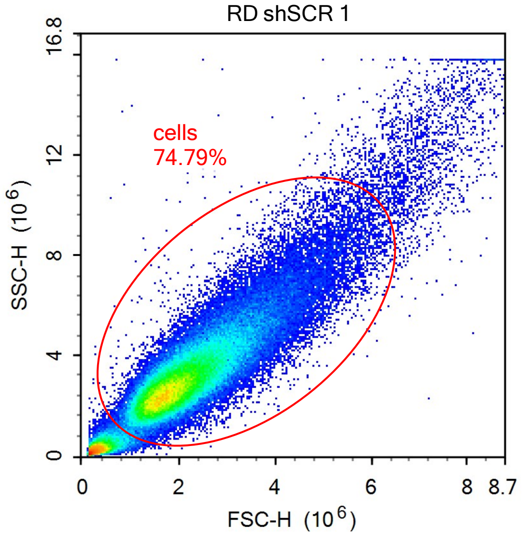

Gate	Count	% All	Median X	Median Y
All	133,706	100.00%	1,792,965	2,576,383
cells	100,000	74.79%	2,062,823	2,977,286

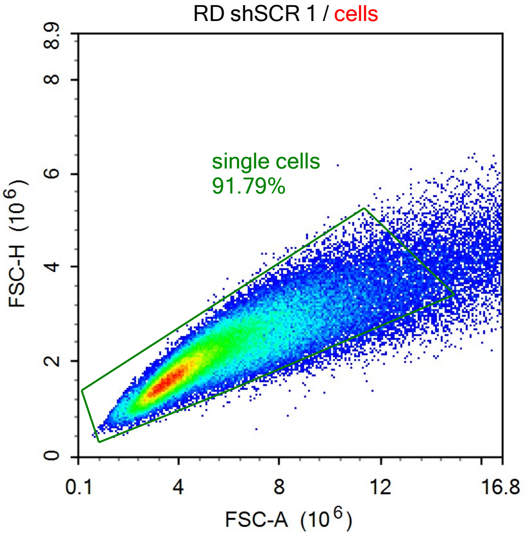

Gate	Count	% cells	Median X	Median Y
cells	100,000	100.00%	4,931,150	2,062,823
single cells	91,792	91.79%	4,673,199	1,985,722

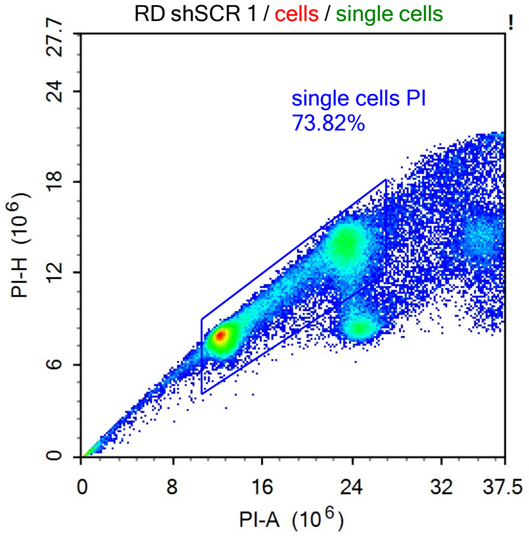

Gate	Count	% single cells	Median X	Median Y
single cells	91,792	100.00%	13,222,062	8,300,717
single cells PI	67,759	73.82%	12,682,464	8,091,018

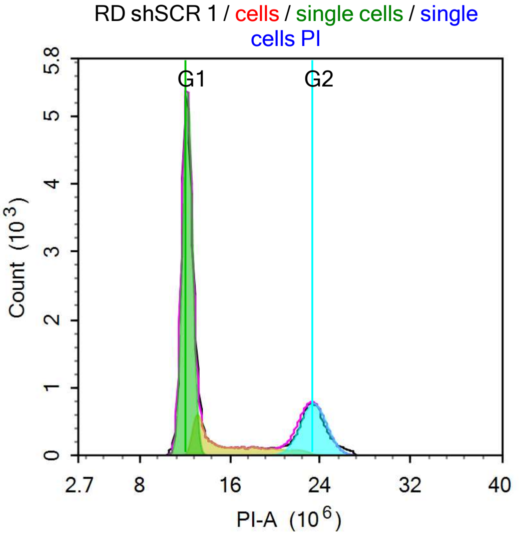

RMS	Freq G1	Freq S	Freq G2	G2/G1	CV G1
31.00	59.91	17.46	22.61	1.91	3.64%

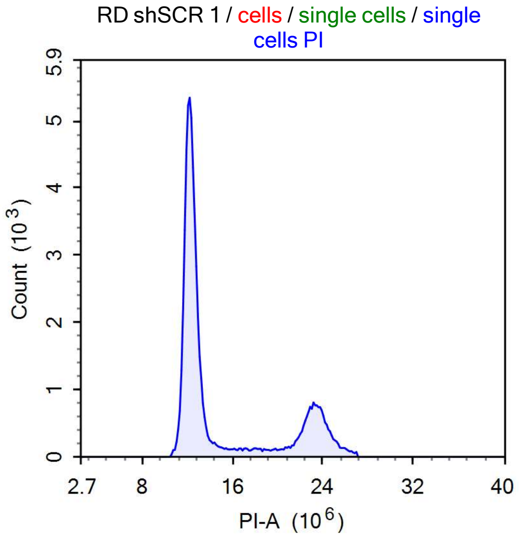

Gate	Count	% single cells PI	Median X
single cells PI	67,759	100.00%	12,682,464

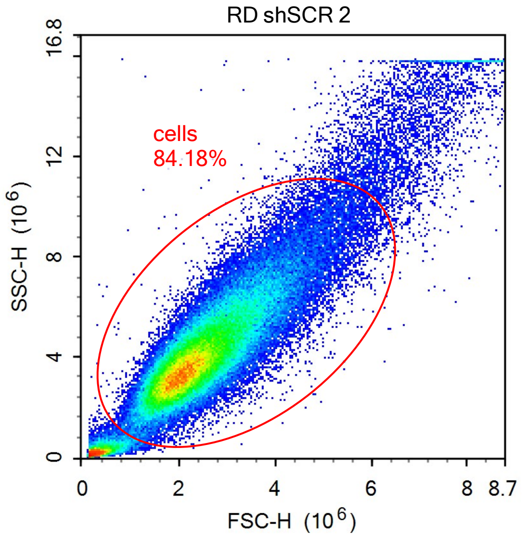

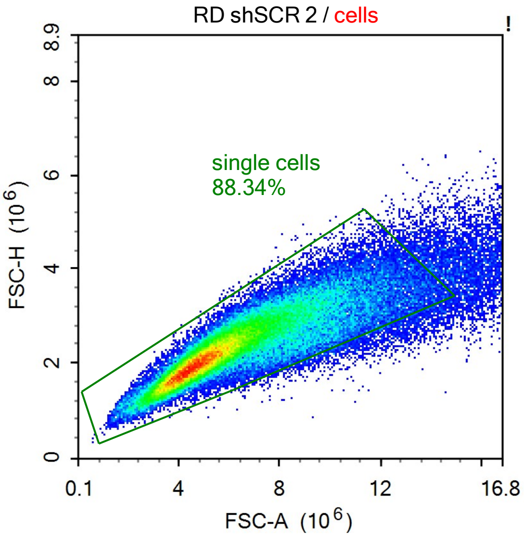

Gate	Count	% All	Median X	Median Y
All	118,791	100.00%	2,301,389	3,766,267
cells	100,000	84.18%	2,400,526	3,943,244

Gate	Count	% cells	Median X	Median Y
cells	100,000	100.00%	6,210,629	2,400,526
single cells	88,342	88.34%	5,787,710	2,293,187

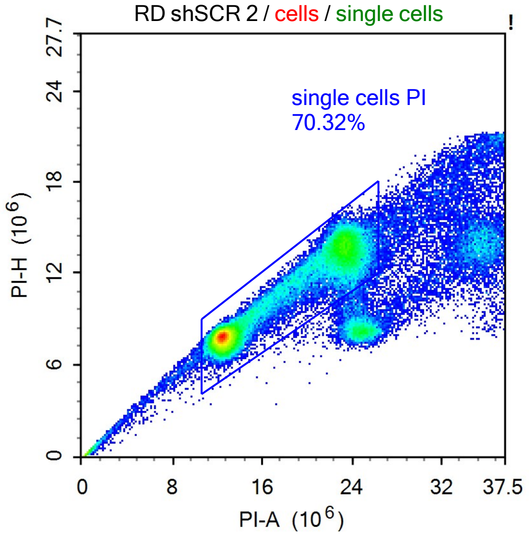

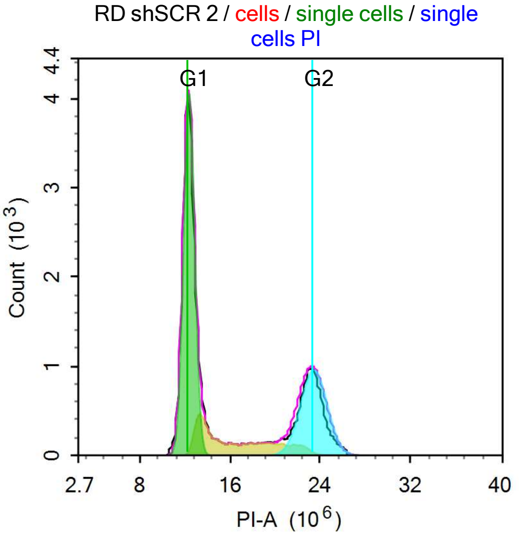

Gate	Count	% single cells	Median X	Median Y
single cells	88,342	100.00%	18,452,736	8,881,382
single cells PI	62,123	70.32%	13,048,115	8,110,331

RMS	Freq G1	Freq S	Freq G2	G2/G1	CV G1
39.77	51.49	19.10	29.40	1.89	3.94%

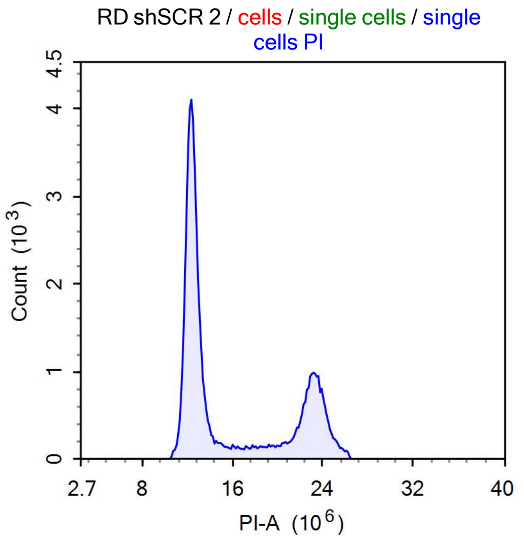

Gate	Count	% single cells PI	Median X
single cells PI	62,123	100.00%	13,048,115

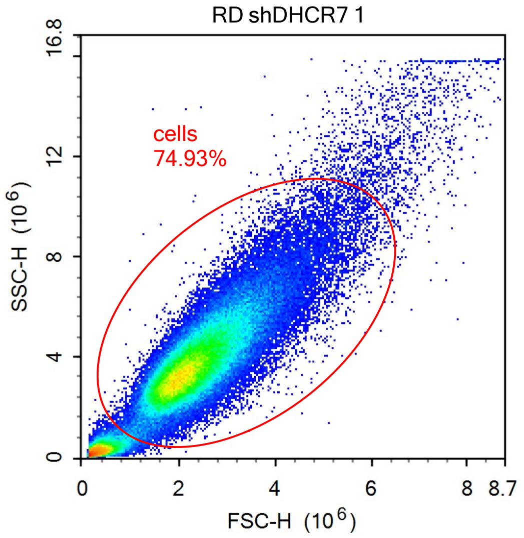

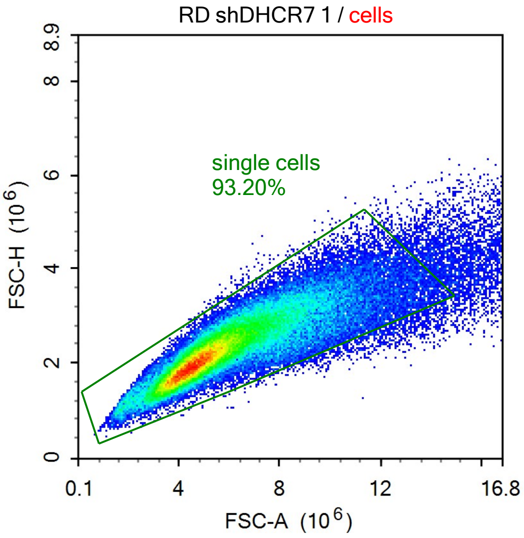

Gate	Count	% All	Median X	Median Y
All	133,464	100.00%	2,059,598	3,211,730
cells	100,000	74.93%	2,317,760	3,685,750

Gate	Count	% cells	Median X	Median Y
cells	100,000	100.00%	5,646,718	2,317,760
single [illegible]	93,195	93.20%	5,451,405	2,260,251

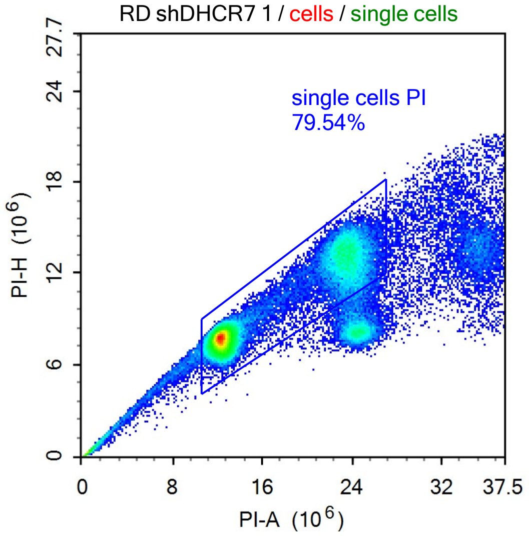

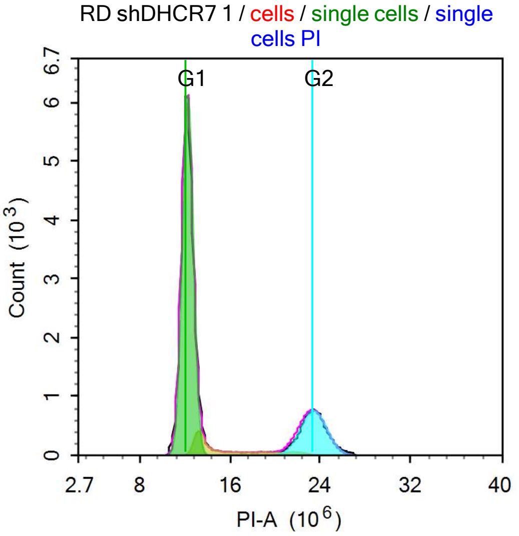

Gate	Count	% single cells	Median X	Median Y
single cells	93,195	100.00%	12,730,948	7,935,304
single cells PI	74,130	79.54%	12,572,372	7,847,610

RMS	Freq G1	Freq S	Freq G2	G2/G1	CV G1
25.28	69.49	9.79	20.73	1.91	4.01%

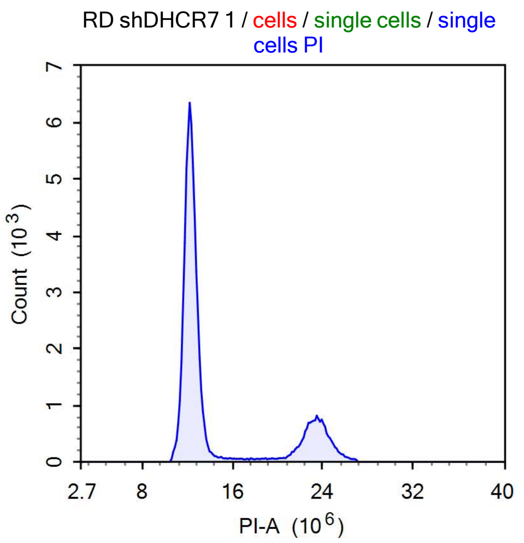

Gate	Count	% single cells PI	Median X
single cells PI	74,130	100.00%	12,572,372

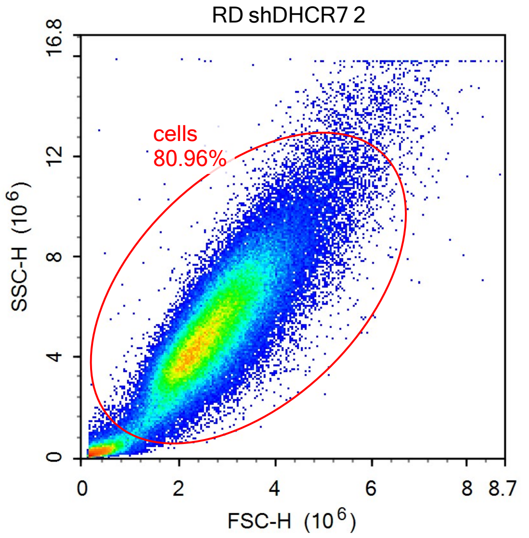

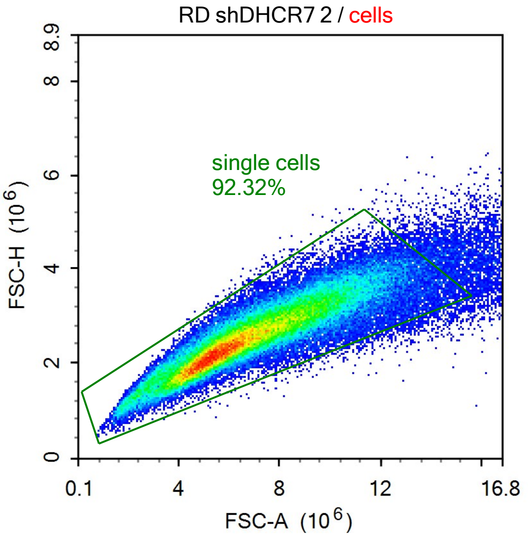

Gate	Count	% All	Median X	Median Y
All	124,678	100.00%	2,424,945	4,418,604
cells	100,936	80.96%	2,648,543	4,943,622

Gate	Count	% cells	Median X	Median Y
cells	100,936	100.00%	7,039,393	2,648,543
single [illegible]	93,189	92.32%	6,725,229	2,572,434

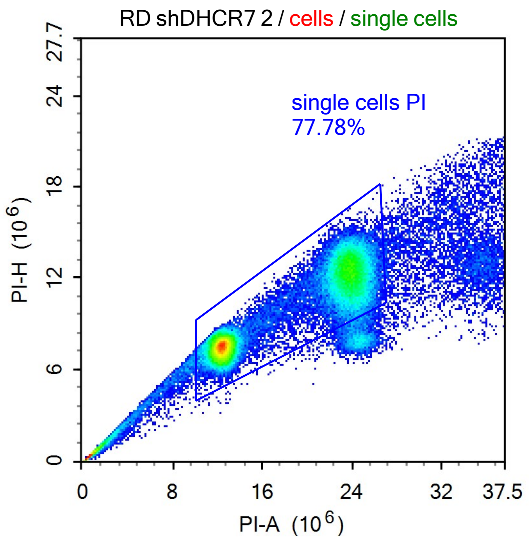

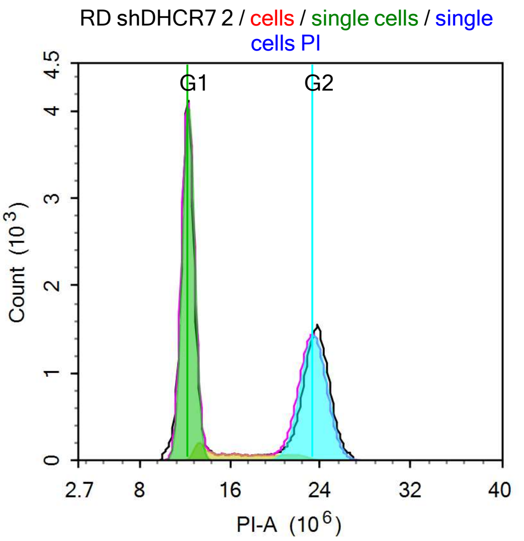

Gate	Count	% single cells	Median X	Median Y
single cells	93,189	100.00%	13,222,610	7,988,631
single cells PI	72,487	77.78%	13,155,869	8,030,039

RMS	Freq G1	Freq S	Freq G2	G2/G1	CV G1
59.24	52.34	7.80	39.64	1.90	4.43%

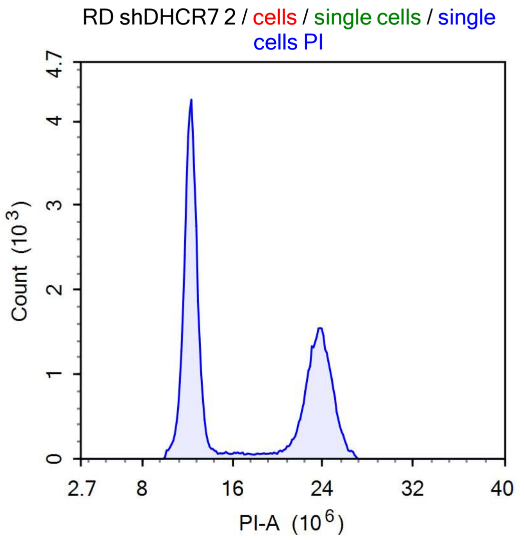

Gate	Count	% single cells PI	Median X
single cells PI	72,487	100.00%	13,155,869
