# Supplementary material for: Inhibiting cholesterol synthesis halts rhabdomyosarcoma growth via ER stress and cell cycle arrest
Source: EMBO Mol Med. 2025 Nov 17;17(12):3586–606. doi: 10.1038/s44321-025-00336-x (PMC12686467; doi:10.1038/s44321-025-00336-x)
Supplement: Supplementary file 9 — Source data Fig. 4 [file 44321_2025_336_MOESM9_ESM.zip › Figure 4/Fig. 4L RD shSCR D2.pdf]

Report of shSCR-RD shSCR D2

Sample Name: shSCR-RD shSCR D2 — Run Time: 7/30/2025 1:43 PM

Cytometer: NovoCyte Quanteon 621210411873 — Software: NovoExpress 1.6.2

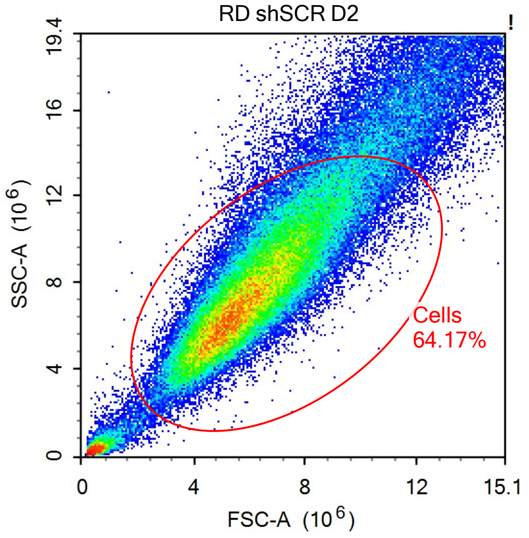

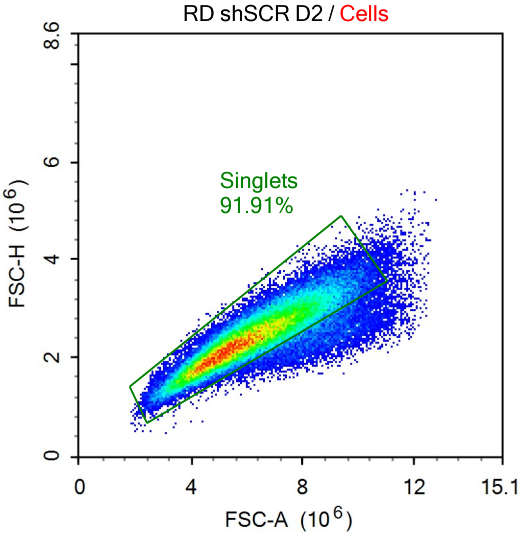

Gate	Count	% All	Median X	Median Y
All	119,637	100.00%	7,097,047	9,121,128
Cells	76,769	64.17%	6,231,053	7,776,014

Gate	Count	% Cells	Median X	Median Y
Cells	76,769	100.00%	6,231,053	2,419,154
Singlet	70,559	91.91%	6,044,621	2,406,697

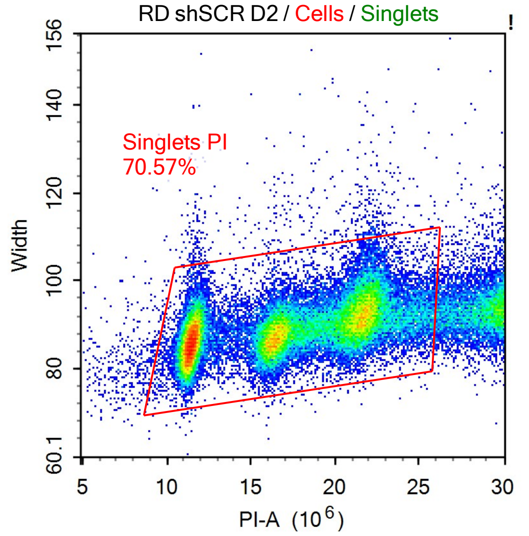

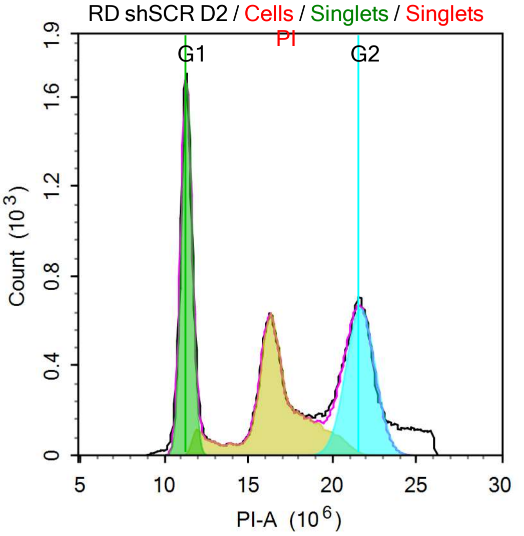

Gate	Count	% Singlets	Median X	Median Y
Singlets	70,559	100.00%	20,788,372	91
Singlets PI	49,791	70.57%	16,970,040	89

RMS	Freq G1	Freq S	Freq G2	G2/G1	CV G1
19.65	29.05	36.56	30.97	1.91	2.83%

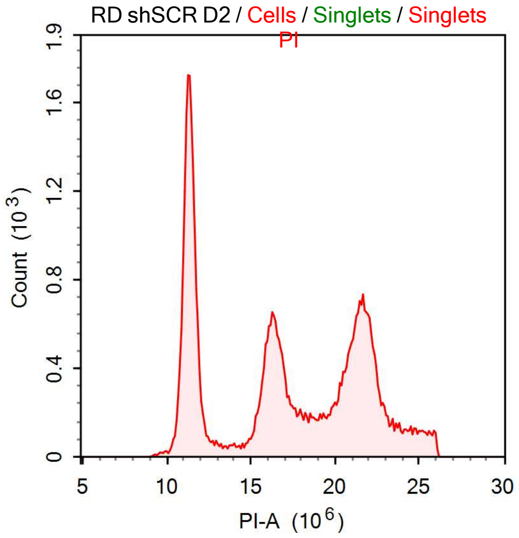

Gate	Count	% Singlets PI	Median X
Singlets PI	49,791	100.00%	16,970,040
