# Supplementary material for: Inhibiting cholesterol synthesis halts rhabdomyosarcoma growth via ER stress and cell cycle arrest
Source: EMBO Mol Med. 2025 Nov 17;17(12):3586–606. doi: 10.1038/s44321-025-00336-x (PMC12686467; doi:10.1038/s44321-025-00336-x)
Supplement: Supplementary file 9 — Source data Fig. 4 [file 44321_2025_336_MOESM9_ESM.zip › Figure 4/Fig. 4L RH30 cell cycle shSCR1 D2.pdf]

Report of Specimen5

Specimen Name: Specimen5 | Run Time: 7/30/2025 1:48 PM
Cytometer: NovoCyte Quanteon 621210411873 | Software: NovoExpress 1.6.2

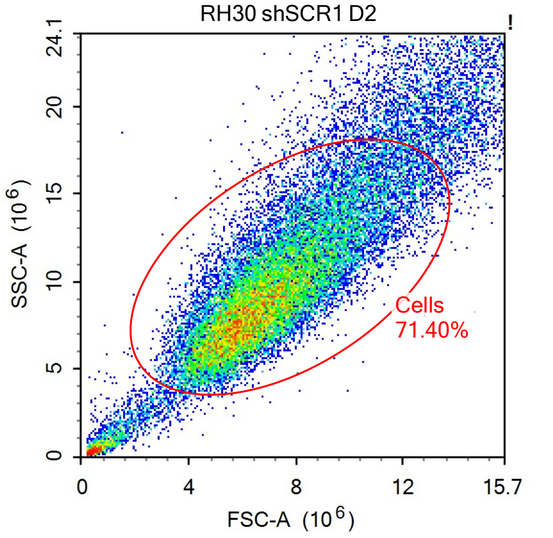

Gate	Count	% All	Median X	Median Y
All	31,262	100.00%	7,655,161	10,477,650
Cells	22,320	71.40%	7,016,971	9,439,171

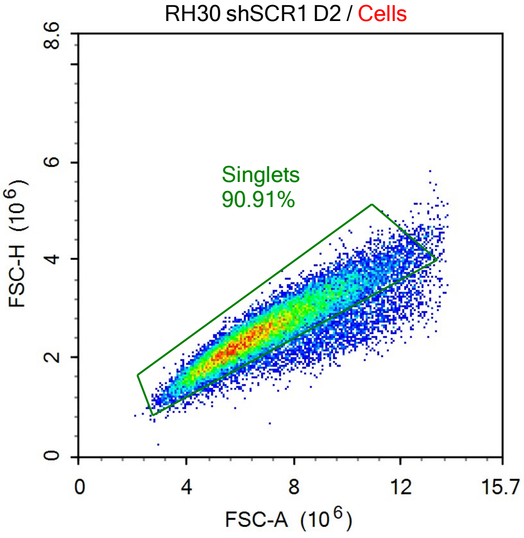

Gate	Count	% Cells	Median X	Median Y
Cells	22,320	100.00%	7,016,971	2,546,354
Singlet	20,292	90.91%	6,801,408	2,545,600

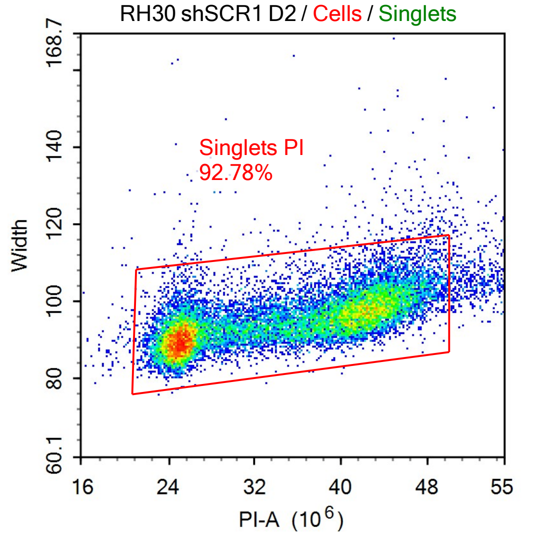

Gate	Count	% Singlets	Median X	Median Y
Singlets	20,292	100.00%	35,652,816	95
Singlets PI	18,827	92.78%	34,586,536	95

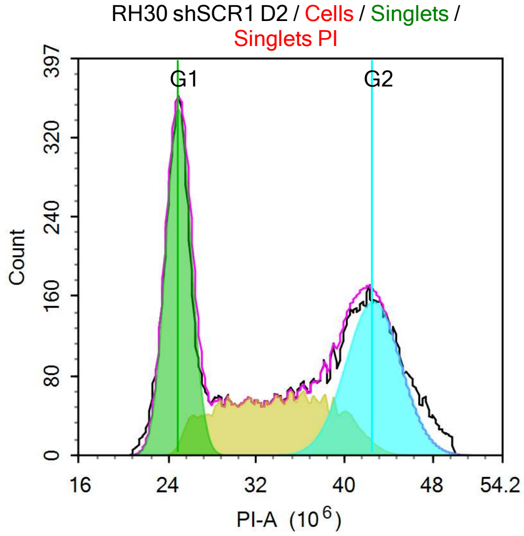

RMS	Freq G1	Freq S	Freq G2	G2/G1	CV G1
7.18	35.53	30.30	34.18	1.71	4.59%

RH30 shSCR1 D2 / Cells / Singlets / Singlets PI

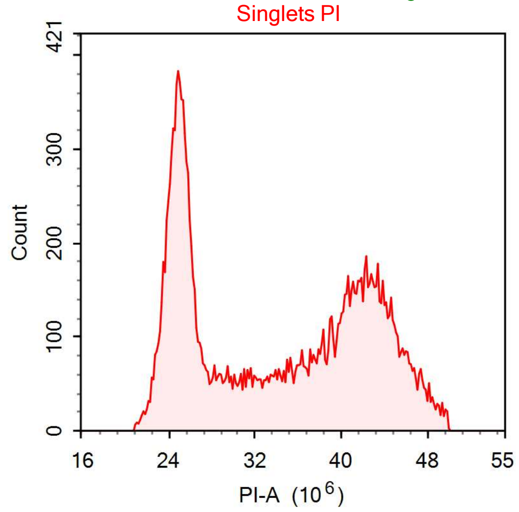

Gate	Count	% Singlets PI	Median X
Singlets PI	18,827	100.00%	34,586,536

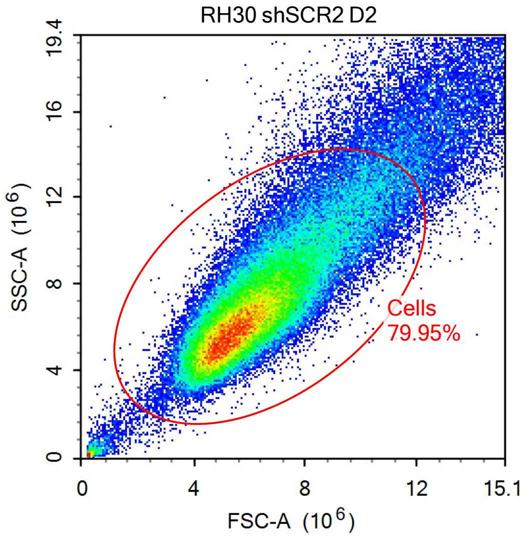

Gate	Count	% All	Median X	Median Y
All	108,745	100.00%	6,623,969	7,463,956
Cells	86,939	79.95%	6,109,068	6,798,624

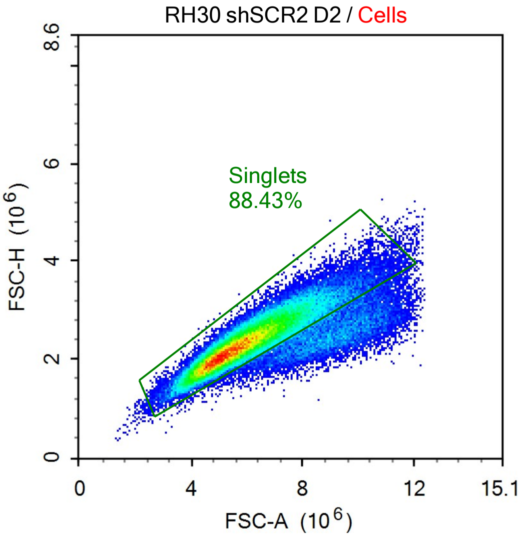

Gate	Count	% Cells	Median X	Median Y
Cells	86,939	100.00%	6,109,068	2,362,680
Singlet	76,882	88.43%	5,869,966	2,337,430

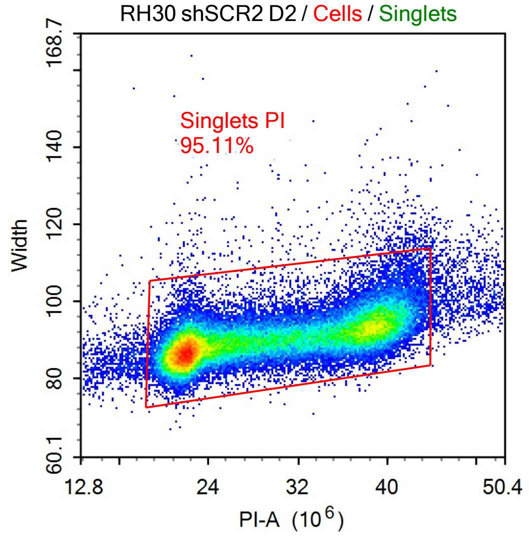

Gate	Count	% Singlets	Median X	Median Y
Singlets	76,882	100.00%	27,021,706	90
Singlets PI	73,124	95.11%	26,830,688	90

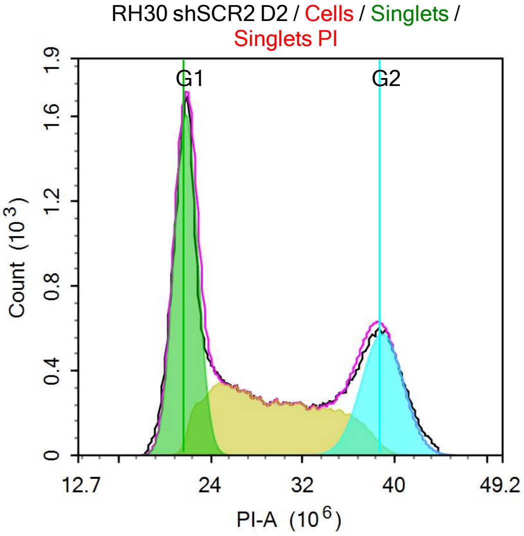

RMS	Freq G1	Freq S	Freq G2	G2/G1	CV G1
32.21	38.09	37.81	24.11	1.78	4.66%

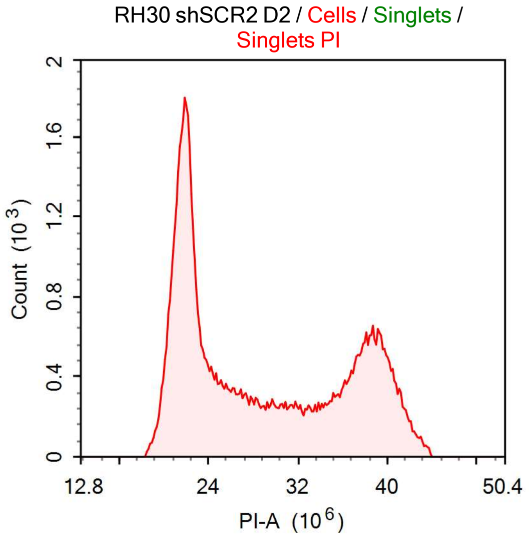

Gate	Count	% Singlets PI	Median X
Singlets PI	73,124	100.00%	26,830,688

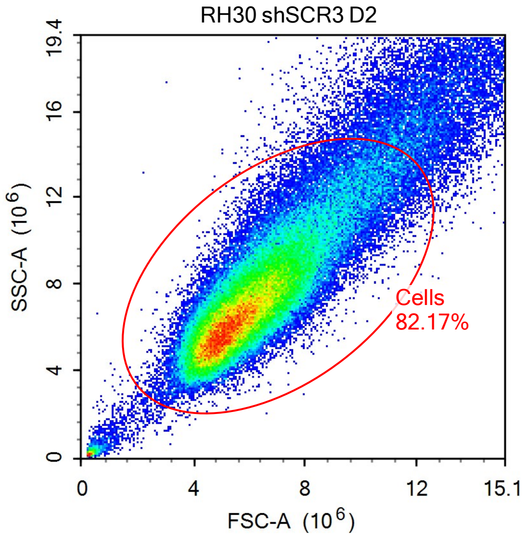

Gate	Count	% All	Median X	Median Y
All	121,702	100.00%	6,457,548	7,688,108
Cells	100,000	82.17%	6,022,069	7,109,078

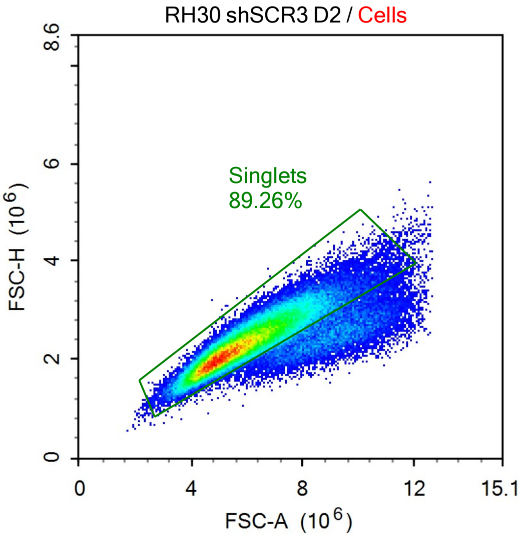

Gate	Count	% Cells	Median X	Median Y
Cells	100,000	100.00%	6,022,069	2,331,673
Singlet	89,256	89.26%	5,791,998	2,304,551

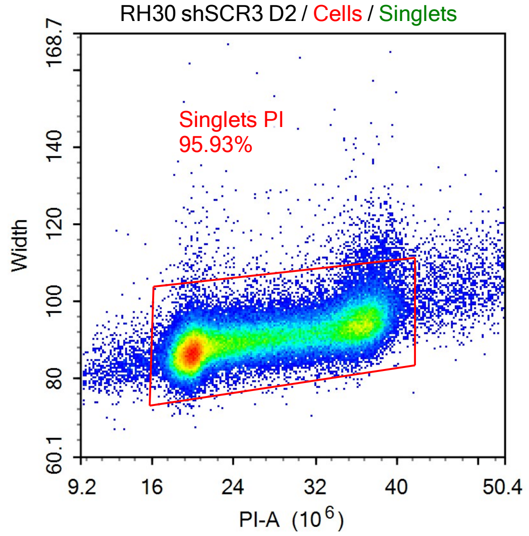

Gate	Count	% Singlets	Median X	Median Y
Singlets	89,256	100.00%	24,135,200	90
Singlets PI	85,622	95.93%	23,956,332	90

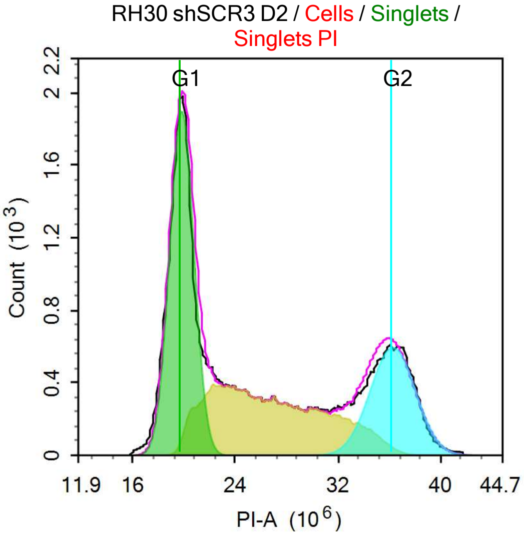

RMS	Freq G1	Freq S	Freq G2	G2/G1	CV G1
36.85	39.24	37.69	22.94	1.83	4.76%

RH30 shSCR3 D2 / Cells / Singlets /
Singlets PI

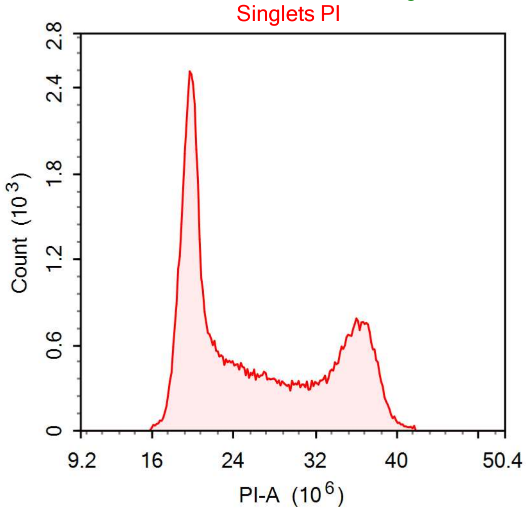

Gate	Count	% Singlets PI	Median X
Singlets [illegible]	85,622	100.00%	23,956,332
