# Supplementary material for: Inhibiting cholesterol synthesis halts rhabdomyosarcoma growth via ER stress and cell cycle arrest
Source: EMBO Mol Med. 2025 Nov 17;17(12):3586–606. doi: 10.1038/s44321-025-00336-x (PMC12686467; doi:10.1038/s44321-025-00336-x)
Supplement: Supplementary file 9 — Source data Fig. 4 [file 44321_2025_336_MOESM9_ESM.zip › Figure 4/Fig. 4L RD shSCR and shDHCR7 cycle cycle.pdf]

Report of Cell Cycle

Specimen Name: PI staining
Run Time: 8/8/2024 10:50 AM
Cytometer: NovoCyte Quanteon 621210411873
Software: NovoExpress 1.6.2

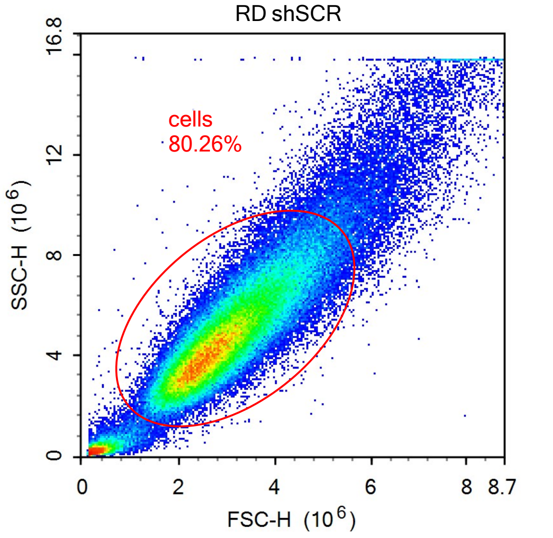

Gate	Count	% All	Median X	Median Y
All	124,981	100.00%	2,881,914	4,555,593
cells	100,304	80.26%	2,932,593	4,650,854

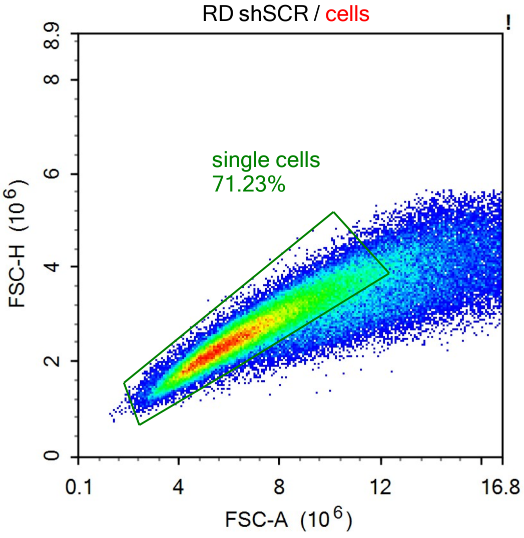

Gate	Count	% cells	Median X	Median Y
cells	100,304	100.00%	8,107,333	2,932,593
single [illegible]	71,448	71.23%	6,817,341	2,652,423

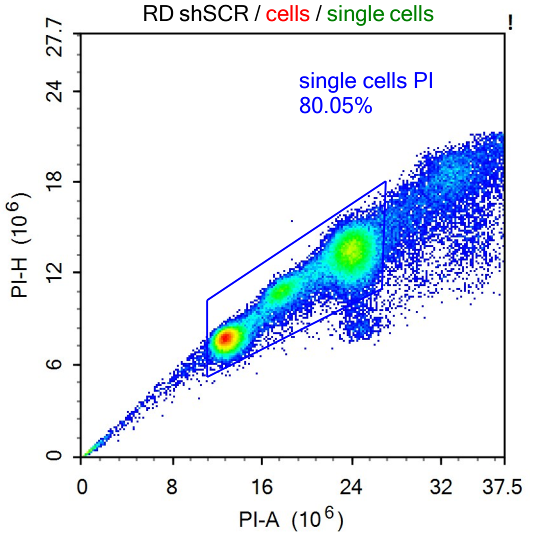

Gate	Count	% single cells	Median X	Median Y
single cells	71,448	100.00%	21,206,872	11,483,380
single cells PI	57,197	80.05%	17,128,348	10,257,898

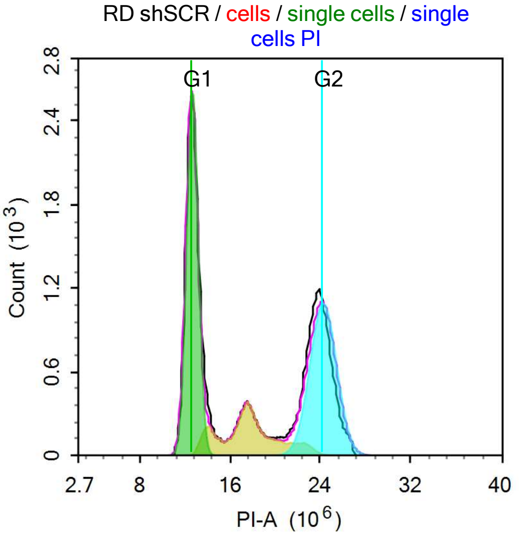

RMS	Freq G1	Freq S	Freq G2	G2/G1	CV G1
36.84	40.95	20.03	39.03	1.91	4.07%

RD shSCR / cells / single cells / single cells PI

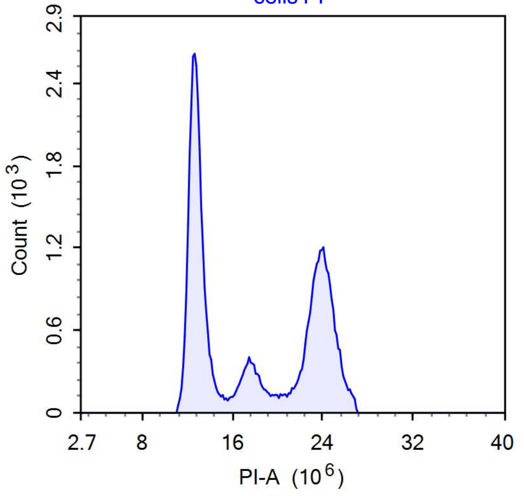

Gate	Count	% single cells PI	Median X
single cells PI	57,197	100.00%	17,128,348

RD shSCR / cells / single cells / single cells PI

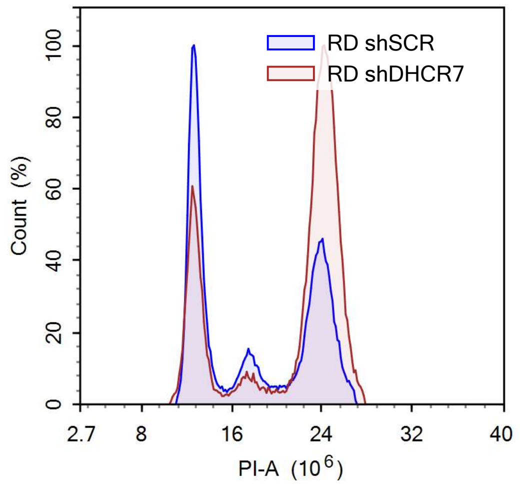

#	Sample	Gate	Count	% single cells PI	Median X
1	RD shSCR	single cells PI	57,197	100.00%	17,128,348
2	RD [illegible]	single cells PI*	57,471	100.00%	23,652,536

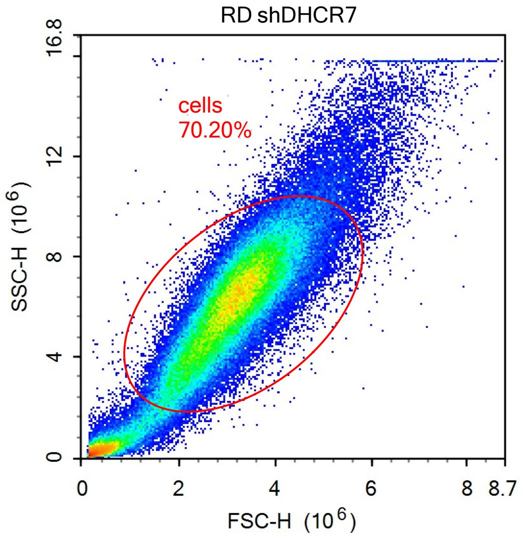

Gate	Count	% All	Median X	Median Y
All	124,697	100.00%	2,869,641	5,522,598
cells	87,536	70.20%	3,164,863	6,275,536

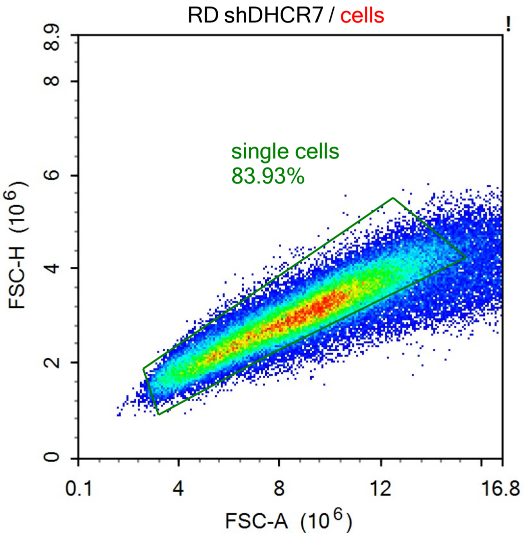

Gate	Count	% cells	Median X	Median Y
cells	87,536	100.00%	9,471,888	3,164,863
single [illegible]	73,469	83.93%	8,872,358	3,055,637

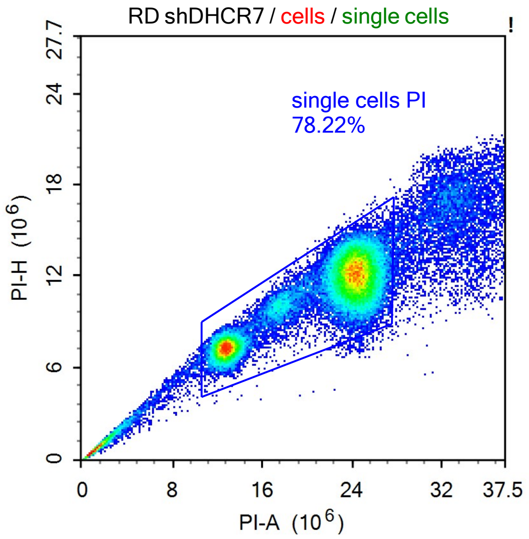

Gate	Count	% single cells	Median X	Median Y
single cells	73,469	100.00%	23,837,422	11,610,041
single cells PI	57,471	78.22%	23,652,536	11,448,293

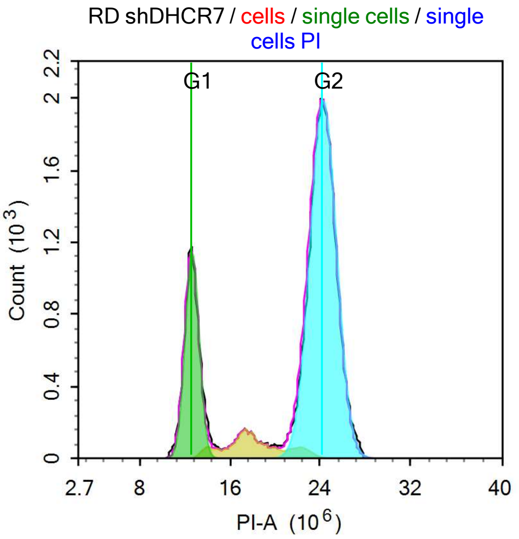

RMS	Freq G1	Freq S	Freq G2	G2/G1	CV G1
16.46	21.22	9.08	69.71	1.92	4.93%

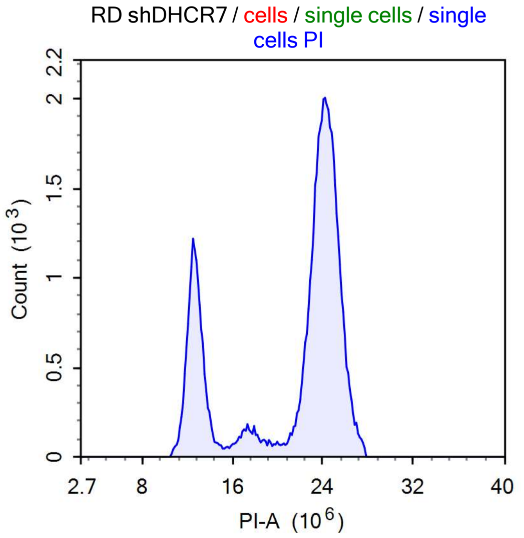

Gate	Count	% single cells PI	Median X
single cells PI	57,471	100.00%	23,652,536
