# Supplementary material for: Inhibiting cholesterol synthesis halts rhabdomyosarcoma growth via ER stress and cell cycle arrest
Source: EMBO Mol Med. 2025 Nov 17;17(12):3586–606. doi: 10.1038/s44321-025-00336-x (PMC12686467; doi:10.1038/s44321-025-00336-x)
Supplement: Supplementary file 9 — Source data Fig. 4 [file 44321_2025_336_MOESM9_ESM.zip › Figure 4/Fig. 4L RD shSCR D1.pdf]

Report of shSCR-RD shSCR D1

Sample Name: shSCR-RD shSCR D1 — Run Time: 7/30/2025 1:08 PM

Cytometer: NovoCyte Quanteon 621210411873 — Software: NovoExpress 1.6.2

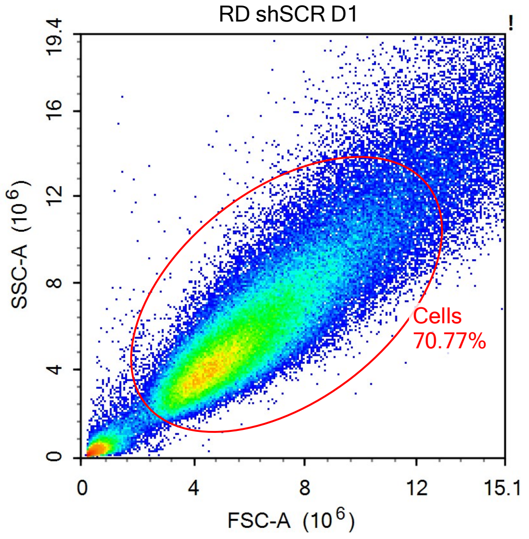

Gate	Count	% All	Median X	Median Y
All	141,726	100.00%	5,338,638	4,974,514
Cells	100,298	70.77%	5,628,934	5,295,496

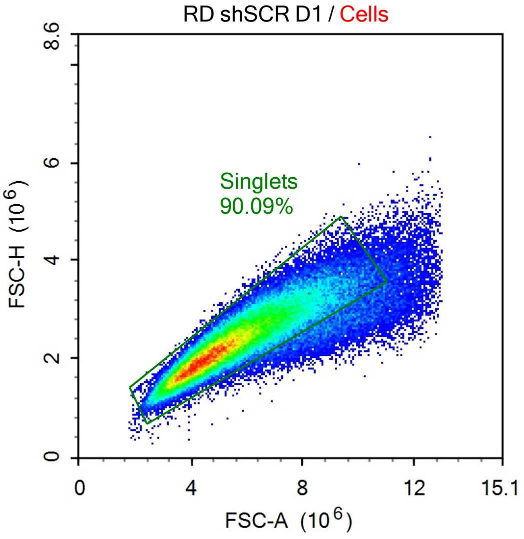

Gate	Count	% Cells	Median X	Median Y
Cells	100,298	100.00%	5,628,934	2,378,268
Singlet[illegible]	90,358	90.09%	5,395,780	2,321,742

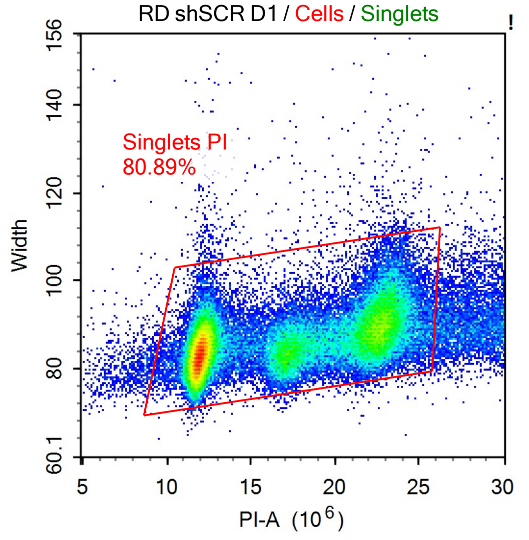

Gate	Count	% Singlets	Median X	Median Y
Singlets	90,358	100.00%	17,444,048	86
Singlets [illegible]	73,095	80.89%	14,020,798	85

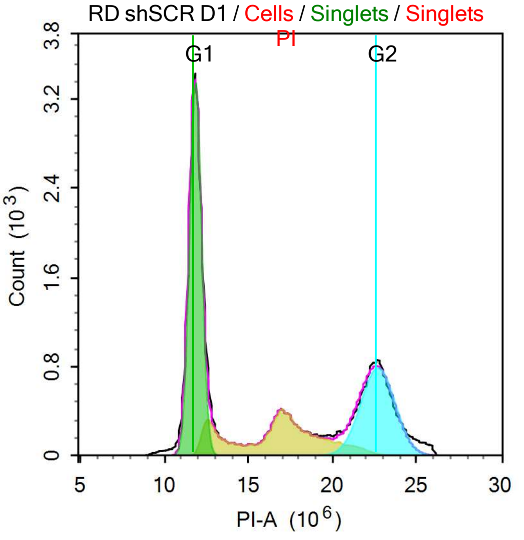

RMS	Freq G1	Freq S	Freq G2	G2/G1	CV G1
20.04	43.47	26.68	29.03	1.91	3.00%

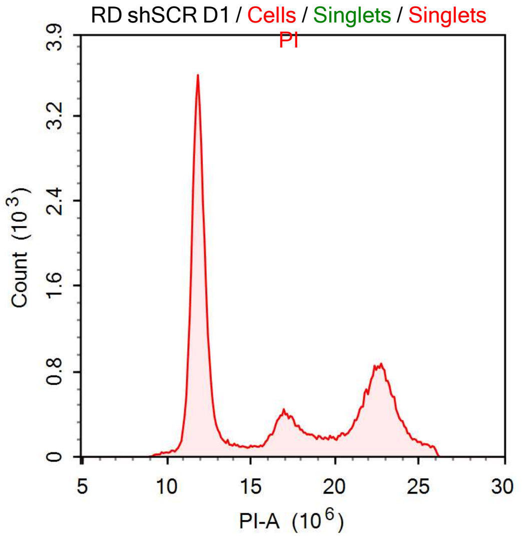

Gate	Count	% Singlets PI	Median X
Singlets PI	73,095	100.00%	14,020,798
