# Supplementary material for: Inhibiting cholesterol synthesis halts rhabdomyosarcoma growth via ER stress and cell cycle arrest
Source: EMBO Mol Med. 2025 Nov 17;17(12):3586–606. doi: 10.1038/s44321-025-00336-x (PMC12686467; doi:10.1038/s44321-025-00336-x)
Supplement: Supplementary file 11 — Expanded View Figures [file 44321_2025_336_MOESM11_ESM.pdf]

Expanded View Figures

Figure EV1. Inhibition of cholesterol biosynthesis impairs rhabdomyosarcoma (RMS) cell growth.

(**A**) Quantitative PCR analysis showing HMGCR mRNA expression levels in RD cells transduced with shSCR (control) or two independent shHMGCR shRNA constructs. Data were presented as mean ± SEM; $N=3$ biological replicates. Statistical analysis: one-way ANOVA analysis followed by Dunnett's multiple comparison test. Significance: $^{**}P < 0.01$, $^{***}P < 0.001$. (**B**) Cell growth was monitored via IncuCyte live cell imaging for RD cells stably expressing shSCR or shHMGCR across two silencing constructs. Data were presented as mean ± SEM; $N=3$ biological replicates. Statistical analysis: one-way ANOVA analysis followed by Dunnett's multiple comparison test. Significance: $^{***}P < 0.001$, $^{****}P < 0.0001$. (**C**, **D**) Colony formation assay in RD cells, along with its quantification. Data were presented as mean ± SEM; $N=3$ biological replicates. Statistical analysis: one-way ANOVA analysis followed by Dunnett's multiple comparison test. Significance: $^{****}P < 0.0001$. (**E**) qPCR analysis of HMGCR mRNA levels in KLHEL1 cells following transduction with shSCR (control) or two independent shHMGCR constructs. Data were presented as mean ± SEM; $N=3$ biological replicates. Statistical analysis: one-way ANOVA analysis followed by Dunnett's multiple comparison test. Significance: $^{**}P < 0.001$, $^{****}P < 0.0001$. (**F**) Cell growth in KLHEL1 cells expressing shSCR or shHMGCR was tracked using IncuCyte live imaging. Data were presented as mean ± SEM; $N=3$ biological replicates. Statistical analysis: one-way ANOVA analysis followed by Dunnett's multiple comparison test. Significance: $^{**}P < 0.001$, $^{****}P < 0.0001$ (**G**, **H**) Colony formation assay and its corresponding quantification in KLHEL1 cells. Data were presented as mean ± SEM; $N=3$ biological replicates. Statistical analysis: one-way ANOVA analysis followed by Dunnett's multiple comparison test. Significance: $^{**}P < 0.001$, $^{****}P < 0.0001$. (**I**, **J**) Growth curves of RD (**I**) and KLHEL1 (**J**) cells treated with different concentrations of lovastatin, analyzed through live cell imaging. Data were presented as mean ± SEM; $N=3$ biological replicates. Statistical analysis: one-way ANOVA analysis followed by Dunnett's multiple comparison test. Significance: $^{****}p < 0.0001$. Exact p values are reported in Table EV2.

© The Author(s)

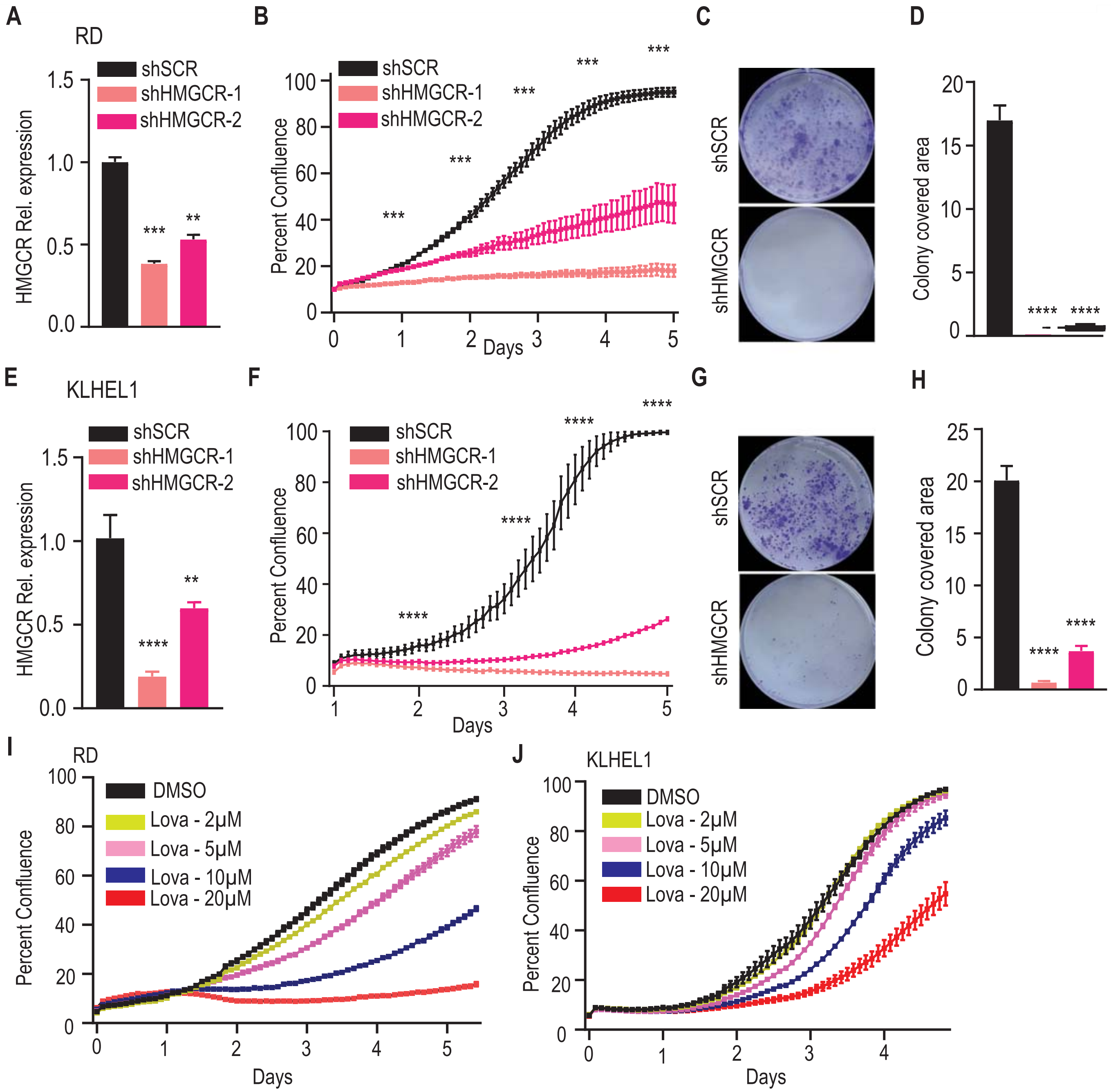
A
RD
shSCR
shHMGCR-1
shHMGCR-2
HMGCR Rel. expression
1.5
1.0
0.5
0.0
***
**
B
shSCR
shHMGCR-1
shHMGCR-2
Percent Confluence
100
80
60
40
20
0
Days
0 1 2 3 4 5
***
C
shSCR
shHMGCR
D
Colony covered area
20
15
10
5
0
****
E
KLHEL1
shSCR
shHMGCR-1
shHMGCR-2
HMGCR Rel. expression
1.5
1.0
0.5
0.0
****
**
F
shSCR
shHMGCR-1
shHMGCR-2
Percent Confluence
Days
1 2 3 4 5
****
G
shSCR
shHMGCR
H
Colony covered area
25
20
15
10
5
0
****
I
RD
DMSO
Lova - 2μM
Lova - 5μM
Lova - 10μM
Lova - 20μM
Percent Confluence
Days
0 1 2 3 4 5
J
KLHEL1
DMSO
Lova - 2μM
Lova - 5μM
Lova - 10μM
Lova - 20μM
Percent Confluence
Days
0 1 2 3 4

 © The Author(s)

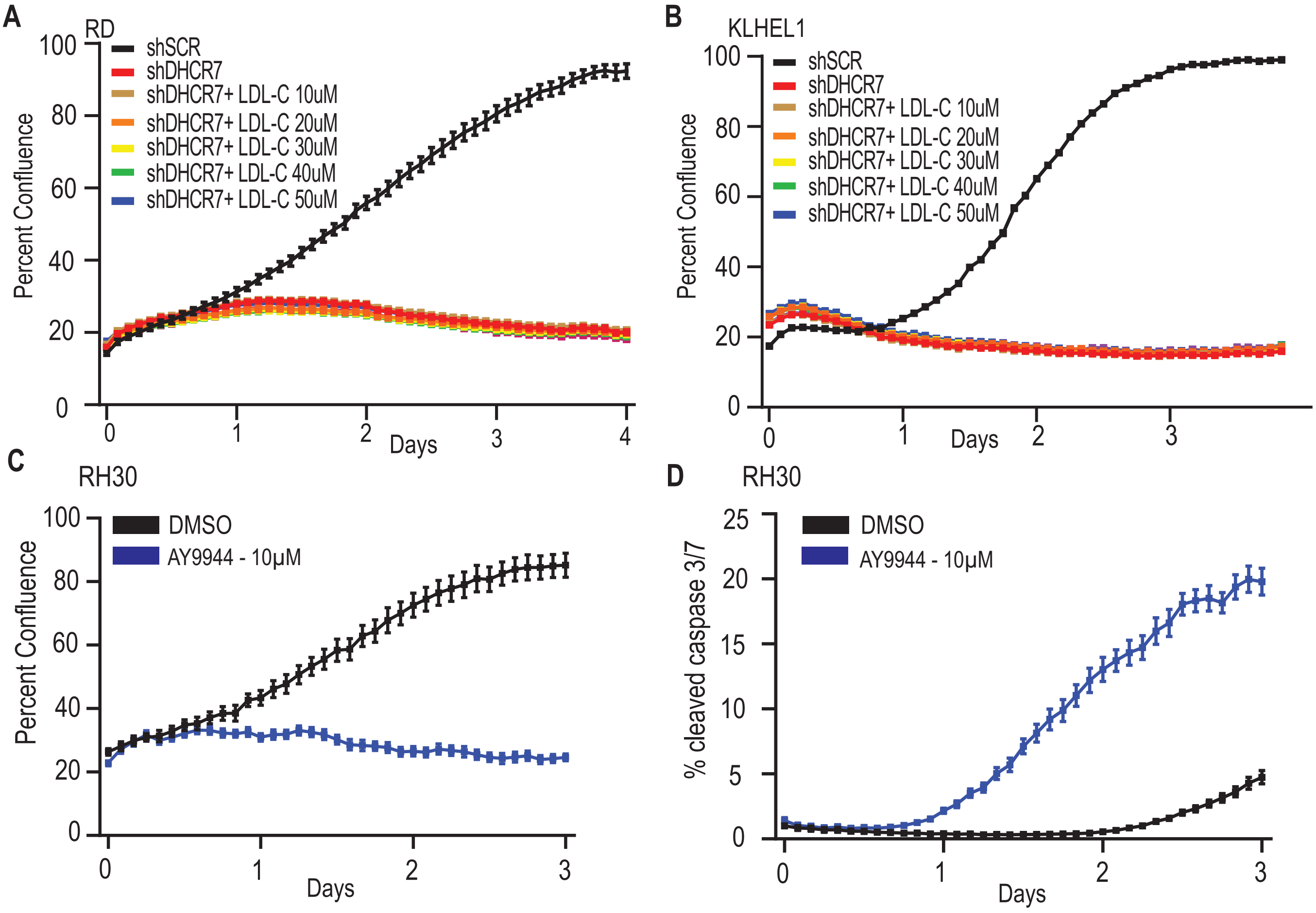

Figure EV2. De novo cholesterol biosynthesis is essential for RMS growth and survival.

(**A**, **B**) Supplementation with varying concentrations of LDL cholesterol (LDL-c) could not reverse the antiproliferative effects of cholesterol biosynthesis inhibition with shDHCR7 in RD (**A**) and KLHEL1 (**B**) cells. (**C**) Growth curves of RH30 cells treated with different concentrations of DHCR7 inhibitor (AY9944) and analyzed by live cell imaging. (**D**) Caspase 3/7 activity in dimethyl sulfoxide (DMSO) and DHCR7 inhibitor-treated RH30 cells. Data are presented as mean ± SEM. ****$P < 0.0001$. Statistical significance was determined using an unpaired two-tailed t-test ($n = 3$ per group). Exact p values are reported in Table EV2.

© The Author(s)

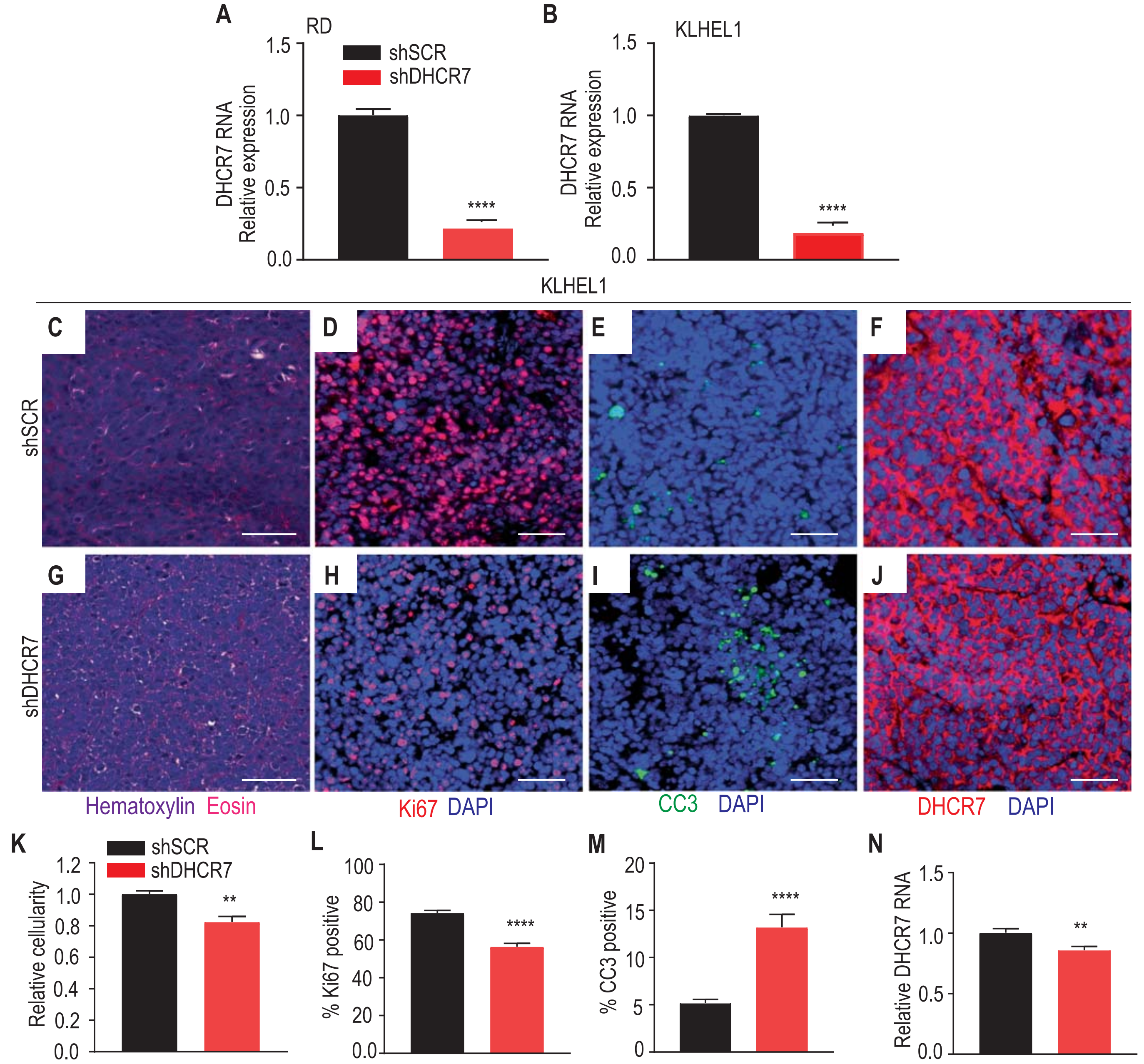

Figure EV3. Cholesterol biosynthesis is essential for RMS tumor xenograft growth.

(**A**, **B**) qPCR analysis of DHCR7 mRNA expression in shDHCR7 and shSCR-transduced KLHEL1 and RD cells prior to tumor implantation. Data were presented as mean ± SEM; $N = 3$ biological replicates. Statistical analysis: unpaired *t*-test (two-tailed). Significance: ****$P < 0.0001$. (**C**, **J**) Histological analysis of KLHEL1 xenograft tumors derived from cells transduced with shSCR (**A–D**) or shDHCR7 (**G–J**). Representative images of H&E-stained tumor sections (**C**, **G**), Ki-67 immunostaining (red) for proliferation and DAPI (blue) (**D**, **H**), cleaved caspase 3 (CC3) immunostaining (green) and DAPI (blue) for apoptosis detection (**E**, **I**) and DHCR7 (red) and DAPI (blue) (**F**, **J**). (**K**) Quantification of nuclei count per tumor area. Data were presented as mean ± SEM; $N = 10$ mice. Statistical analysis: unpaired *t*-test (two-tailed). Significance: **$P < 0.01$. (**L**) Percentage of Ki67-positive proliferating cells within the tumors. Data were presented as mean ± SEM; $N = 10$ mice. Statistical analysis: unpaired *t*-test (two-tailed). Significance: ****$P < 0.0001$. (**M**) Percentage of activated caspase 3 (CC3)-positive cells, indicating apoptosis. Data were presented as mean ± SEM; $N = 10$ mice. Statistical analysis: unpaired *t*-test (two-tailed). Significance: ****$P < 0.0001$. (**N**) qPCR analysis of DHCR7 mRNA expression in tumor tissue. Data were presented as mean ± SEM; $N = 10$ mice. Statistical analysis: unpaired *t*-test (two-tailed). Significance: **$P < 0.01$. Exact *p* values are reported in Table EV2. Scale bar: 100 μm.

 © The Author(s)

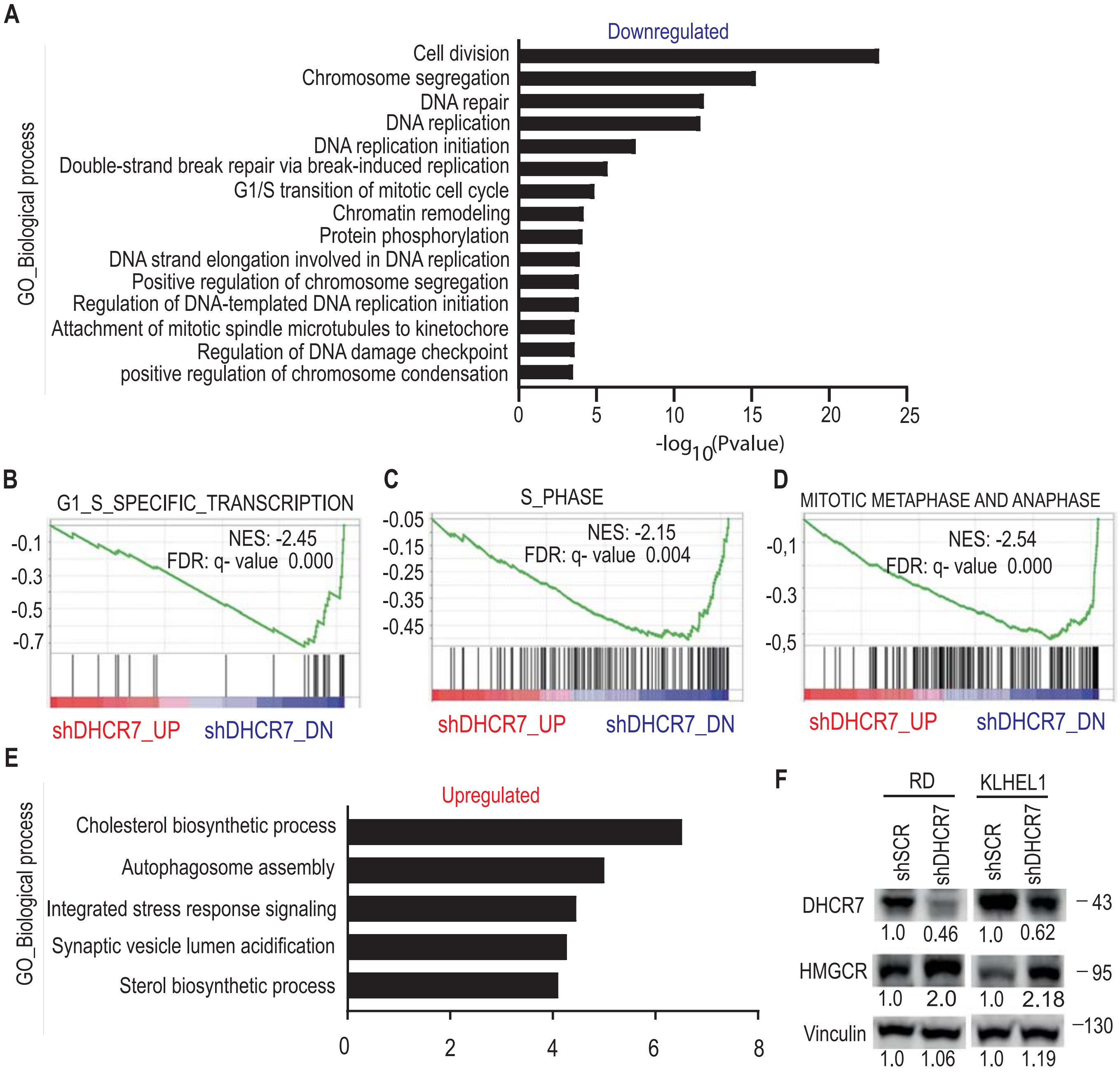

Figure EV4. Cholesterol biosynthesis inhibition disrupts cell cycle progression and induces an integrated stress response.

(**A**) Gene Ontology (GO) analysis showing the most significantly enriched functional categories among the downregulated genes in DHCR7-silenced cells. Statistical analysis: modified Fisher's test. (**B–D**) Gene Set Enrichment Analysis (GSEA) plots illustrating the top functional categories significantly affected in DHCR7-silenced RD cells. Normalized enrichment score (NES) and False Discovery Rate (FDR) values are shown for each pathway. (**E**) GO analysis showing the most significantly enriched functional categories among the upregulated genes in DHCR7-silenced cells. (**F**) Western blot analysis demonstrating that DHCR7 silencing induces compensatory upregulation of HMGCR in RD and KLHEL1 cells, reflecting their reliance on de novo cholesterol biosynthesis. Numbers indicate relative expression levels of DHCR7 and HMGCR normalized to Vinculin. Representative blots from three independent experiments are shown.

© The Author(s)
